# Effect of diagnostic testing on medicines used by febrile children less than five years in 12 malaria-endemic African countries: a mixed-methods study

**DOI:** 10.1186/s12936-015-0709-0

**Published:** 2015-05-10

**Authors:** Emily White Johansson, Peter W Gething, Helena Hildenwall, Bonnie Mappin, Max Petzold, Stefan Swartling Peterson, Katarina Ekholm Selling

**Affiliations:** Department of Women’s and Children’s Health, International Maternal and Child Health, Uppsala University, SE-751 85 Uppsala, Sweden; Spatial Ecology and Epidemiology Group, Department of Zoology, University of Oxford, South Parks Road, OX1 3PS Oxford, UK; Global Health - Health Systems and Policy, Department of Public Health Sciences, Karolinska Institutet, SE-171 77 Stockholm, Sweden; University of Gothenburg, The Sahlgrenska Academy, Health Metrics, Box 414, SE-405 30 Gothenburg, Sweden; School of Public Health, Faculty of Health Sciences, University of the Witwatersrand, Johannesburg, South Africa; Makerere University School of Public Health, College of Health Sciences, PO Box 7072, Kampala, Uganda

**Keywords:** Malaria, Diagnosis, Child health, Fever case management, Population-based surveys, Case studies, Mixed-methods

## Abstract

**Background:**

In 2010, WHO revised guidelines to recommend testing all suspected malaria cases prior to treatment. Yet, evidence to assess programmes is largely derived from limited facility settings in a limited number of countries. National surveys from 12 sub-Saharan African countries were used to examine the effect of diagnostic testing on medicines used by febrile children under five years at the population level, including stratification by malaria risk, transmission season, source of care, symptoms, and age.

**Methods:**

Data were compiled from 12 Demographic and Health Surveys in 2010–2012 that reported fever prevalence, diagnostic test and medicine use, and socio-economic covariates (n = 16,323 febrile under-fives taken to care). Mixed-effects logistic regression models quantified the influence of diagnostic testing on three outcomes (artemisinin combination therapy (ACT), any anti-malarial or any antibiotic use) after adjusting for data clustering and confounding covariates. For each outcome, interactions between diagnostic testing and the following covariates were separately tested: malaria risk, season, source of care, symptoms, and age. A multiple case study design was used to understand varying results across selected countries and sub-national groups, which drew on programme documents, published research and expert consultations. A descriptive typology of plausible explanations for quantitative results was derived from a cross-case synthesis.

**Results:**

Significant variability was found in the effect of diagnostic testing on ACT use across countries (e.g., Uganda OR: 0.84, 95% CI: 0.66-1.06; Mozambique OR: 3.54, 95% CI: 2.33-5.39). Four main themes emerged to explain results: available diagnostics and medicines; quality of care; care-seeking behaviour; and, malaria epidemiology.

**Conclusions:**

Significant country variation was found in the effect of diagnostic testing on paediatric fever treatment at the population level, and qualitative results suggest the impact of diagnostic scale-up on treatment practices may not be straightforward in routine conditions given contextual factors (e.g., access to care, treatment-seeking behaviour or supply stock-outs). Despite limitations, quantitative results could help identify countries (e.g., Mozambique) or issues (e.g., malaria risk) where facility-based research or programme attention may be warranted. The mixed-methods approach triangulates different evidence to potentially provide a standard framework to assess routine programmes across countries or over time to fill critical evidence gaps.

**Electronic supplementary material:**

The online version of this article (doi:10.1186/s12936-015-0709-0) contains supplementary material, which is available to authorized users.

## Background

In 2010, the World Health Organization (WHO) revised guidelines to recommend diagnosis of all suspected malaria cases and treatment based on test results [1], which could greatly improve malaria surveillance, rational drug use and quality fever management [2]. National malaria control programmes are now investing in wide-scale provision of malaria rapid diagnostic tests (RDT) in order to achieve these desired outcomes [3].

Yet, evidence to date shows mixed programme success [4]. While most studies generally indicate a reduction in anti-malarial treatment after RDT introduction [5-8], several also indicate frequent anti-malarial prescriptions despite a negative test result [9-12]. In studies where first-line malaria treatment (artemisinin-based combination therapy (ACT)) was largely restricted to positive cases [13-16], some research shows widespread prescriptions of other anti-malarial [14,17] or antibiotic [13,15] drugs to test-negative patients and not according to established guidelines [18].

However, research has largely been derived from limited health facility settings in a limited number of countries within well-established public health research centres, notably Kenya, Malawi, Tanzania, Uganda, and Zambia [4]. Evidence is limited for most other countries despite, in many cases, comparable RDT investments [19]. While a few countries have conducted national facility studies to examine case management practices across different sub-national contexts [9,20,21], there remains limited understanding of how these practices may differ across key sub-national groups, notably by malaria risk [6,7,12].

Moreover, facility-based studies by their nature do not provide evidence from community settings where paediatric fevers are commonly treated and, perhaps in the future, will be increasingly tested as well [22,23]. There is also limited evidence from routine conditions compared to controlled study trial contexts [24], and how broader programme contexts could influence test-based case management practices (e.g., care-seeking behaviour) [25].

National, population-based, cross-sectional surveys are routinely implemented in sub-Saharan African countries and could be further analysed to provide additional evidence for programmes. Since 2010, these surveys have collected comparable data on malaria diagnostic test use by febrile children under five years [26], although caregivers are not asked about their child’s test result. It is therefore not possible to examine (in)appropriate test-based treatment using these data.

Nevertheless, it is reasonable to expect diagnostic testing to reduce overall ACT use for paediatric fevers at the population level since only a sub-set of tested patients (e.g., test-positive cases) should receive malaria treatment compared to presumptively treating untested cases. Similarly, diagnostic testing could also reduce any anti-malarial use, although reductions may be less marked if second-line treatment is prescribed to test-negative cases [14,17] or caregivers self-treat with other anti-malarial drugs in community settings [25]. It is also plausible that lowered anti-malarial use among tested paediatric fevers could be met with higher antibiotic treatment as an alternative therapy [13,15]. Finally, such drug use changes could be more pronounced among populations with lower fractions of malaria-attributable fevers or where there may be different financial barriers for treatment (e.g., public/private sectors) [22].

In this paper, the effect of diagnostic testing on anti-malarial and antibiotic use among febrile children less than five years was examined in 12 sub-Saharan African countries in 2010–2012, including stratification by malaria risk, transmission season, source of care, child’s age, and symptoms. Given unexpected results, a *post-hoc* analysis using a multiple case study design was employed to understand the complex phenomena driving results in select countries. Such mixed-methods approaches are valuable in health services research to evaluate interventions or answer complex questions [27].

## Methods

This study uses a mixed-methods approach to analyse the effect of diagnostic testing on paediatric fever treatment at the population level across multiple countries, and to plausibly explain findings in select countries.

### Data sources

**Figure 1:**
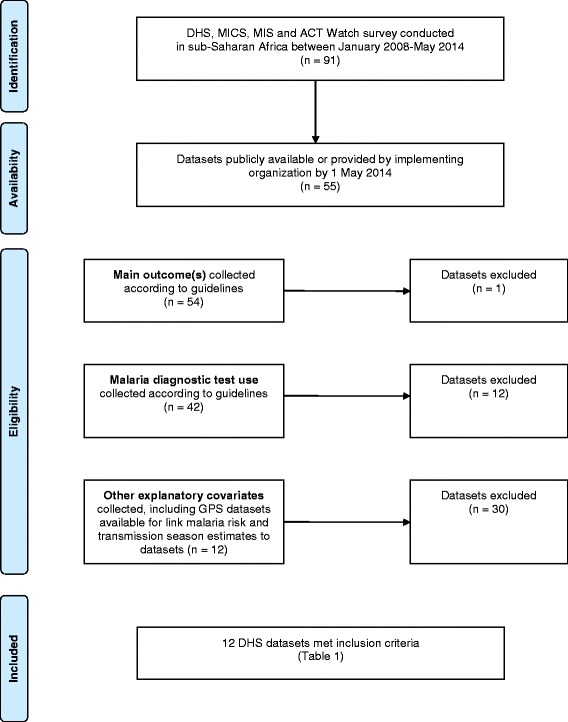
Flow chart for inclusion criteria of country datasets.

National, population-based, cross-sectional surveys conducted in sub-Saharan Africa between 1 January, 2008 and 1 May, 2014 were systematically reviewed for inclusion in this study (Figure 1). All datasets were included if they measured outcome and explanatory covariates as described below. Twelve Demographic and Health Surveys (DHS) in 2010–2012 met inclusion criteria (Table 1). Survey methods are described elsewhere, including procedures for obtaining ethical approval and written informed consent from participants [28].

**Table 1:** **Descriptive statistics for 12 sub-Saharan African countries in 2010-2012**

**Survey**		**n Febrile under-fives taken to any care** ^**a**^	**% receiving a diagnostic test** ^**b**^ **(95% CI)**	**% receiving any anti-malarial drug** ^**c**^ **(95% CI)**	**% receiving ACT** ^**d**^ **(95% CI)**	**% receiving any antibiotic drug** ^**e**^ **(95% CI)**	**Year of national policy change** ^**f**^
Benin DHS 2011-2012	Total	620	20.0 (16.1-24.0)	48.0 (43.2-52.9)	17.5 (14.0-21.0)	28.6 (24.5-32.6)	2011
	*Tested*	*124*	*-*	*59.9 (48.3-71.5)*	*22.1 (14.0-30.3)*	*30.8 (21.3-40.2)*	
	*Not tested*	*492*	*-*	*44.8 (39.8-49.7)*	*16.0 (12.3-19.7)*	*27.9 (23.2-32.5)*	
Burkina Faso DHS 2010-2011	Total	1,823	7.7 (6.1-9.2)	48.0 (44.8-51.1)	12.5 (10.6-14.4)	44.0 (40.9-47.1)	2009
	*Tested*	*140*	*-*	*61.1 (51.9-70.3)*	*16.9 (10.5-23.3)*	*44.2 (35.0-53.4)*	
	*Not tested*	*1,671*	*-*	*46.8 (43.6-50.1)*	*12.1 (10.1-14.1)*	*43.8 (40.6-47.0)*	
Burundi DHS 2010-2011	Total	1,432	36.9 (33.4-40.4)	25.9 (22.8-29.0)	18.0 (15.2-20.9)	54.6 (50.7-58.5)	2007
	*Tested*	*528*	*-*	*38.3 (32.5-44.0)*	*24.9 (19.3-30.6)*	*48.8 (42.7-54.9)*	
	*Not tested*	*895*	*-*	*18.8 (15.7-21.9)*	*14.1 (11.1-17.0)*	*58.1 (53.9-62.2)*	
Cote d’Ivoire DHS 2011-2012	Total	965	13.6 (10.7-16.4)	22.7 (18.8-26.7)	3.3 (1.7-4.9)	34.6 (30.3-38.9)	-
	*Tested*	*131*	*-*	*42.4 (32.1-52.7)*	*9.2 (3.3-15.1)*	*45.9 (35.9-56.0)*	
	*Not tested*	*821*	*-*	*19.2 (15.3-23.2)*	*2.4 (0.7-4.1)*	*33.0 (28.4-37.6)*	
Gabon DHS 2012	Total	738	16.8 (11.3-22.3)	26.2 (20.0-32.4)	10.6 (6.9-14.4)	59.0 (51.5-66.5)	2009
	*Tested*	*124*	*-*	*32.9 (20.5-45.3)*	*8.6 (3.6-13.5)*	*51.2 (37.6-64.8)*	
	*Not tested*	*596*	*-*	*25.0 (17.6-32.3)*	*10.8 (5.9-15.6)*	*60.9 (53.2-68.6)*	
Guinea DHS 2012	Total	931	12.7 (9.8-15.6)	44.3 (39.9-48.7)	2.2 (1.0-3.4)	40.6 (35.9-45.2)	2010
	*Tested*	*118*	*-*	*53.0 (40.9-65.0)*	*4.1 (0.7-7.5)*	*54.6 (43.7-65.4)*	
	*Not tested*	*808*	*-*	*43.0 (38.4-47.7)*	*1.9 (0.6-3.2)*	*38.3 (33.5-43.1)*	
Malawi DHS 2010	Total	4,337	21.4 (19.2-23.6)	55.0 (52.5-57.4)	46.5 (44.0-49.1)	29.6 (27.6-31.7)	2011
	*Tested*	*928*	*-*	*64.2 (59.1-69.3)*	*50.6 (45.3-55.9)*	*33.8 (29.2-38.5)*	
	*Not tested*	*3,363*	*-*	*52.6 (50.1-55.1)*	*45.6 (43.0-48.1)*	*28.5 (26.3-30.7)*	
Mozambique DHS 2011	Total	888	43.4 (38.8-48.1)	41.8 (37.1-46.5)	25.3 (20.7-29.9)	12.0 (9.6-14.5)	2009
	*Tested*	*386*	*-*	*55.5 (48.9-62.0)*	*39.7 (32.5-46.9)*	*12.8 (9.0-16.6)*	
	*Not tested*	*502*	*-*	*31.3 (25.9-36.8)*	*14.3 (10.2-18.3)*	*11.4 (8.0-14.8)*	
Rwanda DHS 2010-2011	Total	657	36.6 (32.6-40.6)	20.4 (17.3-23.5)	19.6 (16.5-22.7)	49.1 (45.0-53.2)	2009
	*Tested*	*240*	*-*	*20.4 (15.5-25.3)*	*19.9 (15.1-24.7)*	*65.4 (59.2-71.6)*	
	*Not tested*	*413*	*-*	*20.6 (16.5-24.7)*	*19.6 (15.6-23.6)*	*39.5 (34.4-44.5)*	
Senegal DHS 2010-2011	Total	1,275	14.9 (11.9-17.9)	14.1 (10.5-17.7)	5.5 (3.1-8.0)	43.0 (37.6-48.3)	2007
	*Tested*	*190*	*-*	*20.4 (11.2-29.6)*	*8.8 (3.6-13.9)*	*51.9 (40.1-63.6)*	
	*Not tested*	*1,078*	*-*	*13.0 (9.4-16.7)*	*4.9 (2.3-7.6)*	*41.5 (36.2-46.8)*	
Uganda DHS 2011	Total	2,440	28.6 (25.5-31.6)	69.9 (67.0-72.7)	47.5 (43.8-51.1)	35.3 (32.3-38.2)	1997
	*Tested*	*697*	*-*	*76.4 (71.9-80.8)*	*52.7 (47.2-58.1)*	*40.9 (35.4-46.5)*	
	*Not tested*	*1,726*	*-*	*67.6 (64.1-71.1)*	*45.5 (41.3-49.7)*	*33.1 (29.7-36.5)*	
Zimbabwe DHS 2010-2011	Total	217	12.6 (7.8-17.5)	5.0 (1.3-8.7)	2.6 (0.2-5.0)	38.8 (31.9-45.8)	2008
	*Tested*	*27*	*-*	*20.5 (3.8-37.1)*	*10.9 (−2.0-23.8)*	*30.2 (12.1-48.3)*	
	*Not tested*	*189*	*-*	*2.8 (0.3-5.2)*	*1.4 (−0.6-3.5)*	*40.1 (32.7-47.5)*	

National point estimates were tabulated using sample weights pre-specified in datasets. Standard error estimation accounted for data clustering in survey designs.

^a^Children under five years old reportedly having fever in the two weeks prior to the interview and taken to any source of care.

^b^Children under five years old with fever in the previous two weeks taken to any care and reportedly receiving a finger or heel stick for testing.

^c^Children under five years old with fever in the previous two weeks taken to any care and reportedly receiving any anti-malarial drug of any type.

^d^Children under five years old with fever in the previous two weeks taken to any care and reportedly receiving ACT.

^e^Children under five years old with fever in the previous two weeks taken to any care and reportedly receiving any antibiotic drug of any type.

^f^[60] Refers to year national policy changed to recommend parasitological diagnosis in patients of all ages prior to treatment.

### Outcomes

Paediatric fever treatment was measured by asking caregivers of children under five with reported fever in the previous two weeks if “At any time during the illness did (name) take any drugs, and if so, what drugs did (name) take?” Response categories included anti-malarial drugs (by type), antibiotic drugs (pill/syrup or injection) or other medicines. Multiple responses were allowed and sick children receiving dual treatment were categorized as having positive outcomes for both responses. Anti-malarial medicines reported include ACT, chloroquine, sulphadoxine-pyrimethamine (SP)/Fansidar, quinine and other country-specific brands. Any anti-malarial use included all anti-malarial drugs reportedly used to treat the fever illness while ACT use referred to that treatment alone. Any antibiotic use referred to either pill/syrup or injection antibiotic drugs, and was not further disaggregated by type in response categories.

### Main explanatory predictor

Malaria diagnostic test use was measured by asking caregivers of febrile children if “At any time during the illness did (name) have blood taken from his/her finger or heel for testing?” This question was assumed to refer to either microscopy or RDT. The questionnaire did not explicitly record where testing and treatment occurred, nor if these interventions were received together. 812 (5%) children across 12 countries taken to multiple sources were excluded in order to assume that both interventions were provided at the same source.

### Other explanatory covariates

The model included other covariates associated with both diagnosis and treatment, which were grouped into individual, household and community factors [29-31]. Individual factors included child’s sex and age (0–5, 6–11, 12–23, 24–35, 36–47, 48–59 months), maternal age (15–24, 25–29, 30–34, 35–39, 40–49 years) and education (none, primary or at least secondary), and symptoms (fever alone, fever with cough, and fever with cough and rapid breaths). The latter covariate was also used to proxy illness severity that was not directly measured in surveys, and multiple symptoms were assumed to reflect more severe cases [32].

Household factors included wealth and size, care-seeking behaviour, and access to testing and care. A wealth index was pre-specified in datasets and described elsewhere [33]. Household size was categorized as one to four, five to eight, nine to 12, and 13 or more household members [34]. Care-seeking behaviour was based on caregiver reports of where care was sought for the sick child, and was separately coded by level of care (hospital, non-hospital formal medical, community health worker (CHW), pharmacy, and other) and sector (public, private) [35,36]. Access to testing and care was based on caregivers’ perceptions that money or distance is a “big problem” or “not a big problem” to seeking medical advice or treatment. These two covariates, along with child health card possession, were used to attempt to proxy attendance at a facility stocked with both drugs and diagnostic tests, which is known to influence case management decisions but is not directly measured in surveys.

Community factors included residence (urban/rural), malaria risk and transmission season. Malaria Atlas Project malaria endemicity estimates were linked to datasets through geocoded primary sampling units (PSUs) [37]. All individual observations were assigned their PSU-level malaria risk value and categorized as malaria-free, unstable, low (*Pf*PR_2–10_ < 5%), moderate (*Pf*PR_2–10_ 5%-40%), and high (*Pf*PR_2–10_ > 40%) stable endemic transmission. Each observation was also classified as occurring during or outside the peak malaria transmission season by comparing each observation’s PSU location and interview date with seasonality maps produced by the Mapping Malaria Risk in Africa (MARA) project [38].

Among 16,323 surveyed febrile children under five taken to care in 12 countries, 17 had missing values for the outcomes, 24 for diagnostic test use, 309 for malaria endemicity and transmission season, seven for health card and one for maternal education. List-wise deletion was used to exclude these observations.

### Statistical analysis

Mixed-effects logistic regression models quantified the influence of diagnostic testing on paediatric fever treatment among children taken to care in each country dataset. The binary outcomes analysed were: (1) ACT use; (2) any anti-malarial use; and, (3) any antibiotic use. All covariates were included as categorical fixed effects (first-level) nested within PSUs (second-level), and normal distribution of the random effects was assumed. Crude odds ratios for the main covariate were initially estimated for its effect on each outcome, and were subsequently adjusted for the effect of all covariates. For each outcome, interactions between diagnostic testing and the following covariates were separately tested: malaria risk, season, source of care, age, and symptoms. If there was evidence of an interaction, final models were stratified accordingly to explore results. The level of statistical significance was set to 0.05. National point estimates were tabulated using sample weights to account for unequal probabilities of selection in order to generate nationally representative weighted percentages. Standard error estimation accounted for data clustering in the complex survey design. Stata 12 (STATA Corp, College Station, TX) was used for all analyses.

### Case study methods

A multiple case study design was employed to help understand results in selected countries and drew on published research, programme documents and expert consultations [27]. Country selection was based on the following criteria: (1) contrasting quantitative results; (2) high ACT coverage; and, (3) available research or programme documents. Benin, Burundi, Malawi, Mozambique, Rwanda and Uganda were selected for case studies.

A comprehensive literature review identified published articles on malaria diagnosis and treatment practices in these countries. Benin and Malawi had national facility studies conducted around the same time to help explain results [9,20], while Uganda, Malawi and Mozambique had relevant research to support case studies [12,16,39,40]. National malaria strategic plans for Malawi, Mozambique and Uganda were made available for this study [41-43], and all six countries had US President’s Malaria Initiative operational plans [44]. These materials were reviewed to identify potential explanations for quantitative findings, inform the topic guide used in expert consultations, and cross-reference interview information to confirm conclusions.

For expert consultations, seven respondents were purposively selected based on their country programme knowledge and advanced research training. Five informants were identified and contacted by study authors (EWJ, SP) while two others were introduced by initial respondents using snowballing and convenience sampling techniques. Participants included university researchers, paediatricians and epidemiologists with expert knowledge of national malaria control programmes. Prior to involvement, respondents were given detailed information about the study’s objectives, methods and full quantitative results. Respondents were also invited to review case studies as well as the final manuscript.

Interviews were based on a semi-structured topic guide that focused on the plausibility of results, programme explanations and perceived value of findings as additional programme evidence. Specific themes included: RDT scale-up status; availability of diagnostics and medicines; stock-outs; case management practices; health system structure; care-seeking behaviour; and, malaria epidemiology.

The lead author (EWJ) conducted seven interviews in English via Skype or in person during July-September 2014 (one for each country; two for Benin) each lasting about one hour. Extensive written notes were taken during interviews and transcribed after discussions. Explanation building leading to a cross-case synthesis was the overall analytic strategy [45]. This approach emphasizes defining and testing rival explanations as part of the design, and compiling data from multiple sources to triangulate evidence and evaluate rival interpretations [27]. Thematic analysis identified dominant themes within each case [46]. All transcripts were read multiple times by the lead author to establish preliminary codes and create categories to describe response patterns. Matrices helped to visually examine codes in order to generate within-case themes, and to subsequently compare and revise themes across countries. This led to a typology of plausible explanations for quantitative results for the six countries.

## Results

### Quantitative results

16,323 children under five years had fever in the previous two weeks and were taken to any care across 12 countries (Table 1). 3,633 of these children received a diagnostic test with national coverage ranging between 8% and 43%. Across the 12 countries, 7,154 children received any anti-malarial drug, 4,332 received ACT, and 6,115 received any antibiotic drug according to caregiver reports.

Table 2 presents the association between the main predictor and the three outcomes (ACT use, any anti-malarial use and any antibiotic use) in each of the 12 countries. Results for other covariates included in the final country models are provided in additional files (Additional file 1 and Additional file 2). These results indicate that no studied country had significantly reduced odds of malaria treatment for tested paediatric fevers compared to untested, which is the opposite of the stated hypothesis. However, there was variability in the effect of diagnostic testing on paediatric fever treatment across countries.

**Table 2:** **Effect of diagnostic testing on paediatric fever treatment in 12 studied countries in 2010-2012**

**Country**	***n*** **Febrile under-fives taken to any care**	***n*** **any anti-malarial drug use**	**Any anti-malarial use**			***n*** **ACT use**	**ACT use**			***n any*** **antibiotic drug use**	**Any antibiotic use**		
			**COR (95% CI)**	**AOR (95% CI)**	**p value**		**COR (95% CI)**	**AOR (95% CI)**	**p value**		**COR (95% CI)**	**AOR (95% CI)**	**p value**
Benin	620	298	2.61 (1.51-4.51)	1.65 (0.92-2.98)	0.096	109	2.37 (1.20-4.70)	1.96 (0.91-4.19)	0.084	177	1.83 (1.06-3.17)	1.15 (0.64-2.08)	0.636
Burkina Faso	1,823	875	2.08 (1.39-3.11)	1.32 (0.84-2.05)	0.225	228	1.64 (0.99-2.71)	1.45 (0.84-2.52)	0.180	803	1.22 (0.82-1.81)	0.89 (0.57-1.40)	0.616
Burundi	1,432	371	3.62 (2.64-4.96)	3.71 (2.63-5.25)	<0.001	258	2.62 (1.79-3.83)	2.78 (1.81-4.27)	<0.001	782	0.62 (0.47-0.81)	0.53 (0.40-0.72)	<0.001
Cote d’Ivoire*	965	220	3.29 (2.05-5.25)	1.89 (1.14-3.13)	0.013	32	7.09 (2.45-20.54)	16.83 (1.03-276.13)	0.048	334	1.99 (1.31-3.01)	1.08 (0.68-1.74)	0.737
Gabon	738	194	2.25 (1.40-3.61)	2.00 (1.16-3.44)	0.013	78	2.74 (1.45-5.16)	2.45 (1.13-5.33)	0.024	436	0.88 (0.58-1.35)	0.84 (0.52-1.35)	0.467
Guinea*	931	412	1.70 (1.08-2.67)	1.28 (0.78-2.11)	0.330	20	4.29 (1.25-14.68)	2.42 (0.43-13.68)	0.319	378	1.76 (1.11-2.78)	1.05 (0.63-1.75)	0.862
Malawi	4,337	2,384	1.65 (1.40-1.94)	1.34 (1.11-1.61)	0.002	2,019	1.26 (1.07-1.48)	1.12 (0.94-1.34)	0.206	1,285	1.12 (0.94-1.33)	1.00 (0.82-1.22)	1.000
Mozambique	888	371	2.85 (2.02-4.02)	2.79 (1.92-4.05)	<0.001	225	3.65 (2.45-5.42)	3.54 (2.33-5.39)	<0.001	107	1.04 (0.67-1.61)	1.01 (0.64-1.59)	0.966
Rwanda*	657	134	0.93 (0.57-1.52)	0.83 (0.48-1.44)	0.506	129	0.96 (0.59-1.56)	0.88 (0.51-1.51)	0.633	322	3.70 (2.38-5.74)	2.95 (1.82-4.79)	<0.001
Senegal	1,275	180	1.75 (1.11-2.75)	1.69 (1.04-2.76)	0.036	70	2.54 (1.24-5.19)	2.99 (1.32-6.79)	0.009	547	1.90 (1.27-2.85)	1.50 (0.97-2.31)	0.070
Uganda	2,440	1,704	1.50 (1.19-1.89)	1.24 (0.96-1.61)	0.097	1,158	1.13 (0.92-1.39)	0.84 (0.66-1.06)	0.133	860	1.45 (1.18-1.78)	1.37 (1.09-1.72)	0.007
Zimbabwe*	217	11	13.23 (1.56-112.52)	170.9 (0.30-98480.04)	0.113	6	12.18 (1.94-76.45)	25.55 (1.69-385.68)	0.019	84	0.62 (0.24-1.60)	0.55 (0.20-1.51)	0.244

CI = confidence interval. AOR = adjusted odds ratio. COR = crude odds ratio. AORs based on mixed-effects logistic regression models in individual country datasets adjusted for data clustering and confounding covariates (malaria endemicity; transmission season; public/private source; level of care; child’s age and sex; maternal age and education; residence; household wealth and size; health care access (money); health care access (distance); symptoms; health card).

*Rwanda’s model does not include the ‘level of care’ covariate due to multi-collinearity with the public/private source covariate. Guinea’s model does not include ‘money or distance as problems accessing care’ covariates. Some results should be interpreted with caution due to few observations and few positive outcomes (e.g., Cote d’Ivoire, Guinea, Zimbabwe).

### ACT use

In six countries, tested paediatric fevers had significantly higher ACT use odds compared to untested cases according to caregiver reports (Burundi, Côte d’Ivoire, Gabon, Mozambique, Senegal, Zimbabwe), although Zimbabwe and Côte d’Ivoire results should be interpreted with caution due to few observations and positive outcomes. Burundi and Mozambique were among the countries with highest ACT use odds for tested paediatric fevers compared to untested ones (Burundi OR: 2.78, 95% CI: 1.81-4.27; Mozambique OR: 3.54, 95% CI: 2.33-5.39). In contrast, Rwanda and Uganda had relatively lower odds of ACT use associated with testing (Rwanda OR: 0.88, 95% CI: 0.51-1.51; Uganda OR: 0.84, 95% CI: 0.66-1.06).

### Any anti-malarial use

Six countries demonstrated significantly higher anti-malarial use odds associated with reported testing (Burundi, Côte d’Ivoire, Gabon, Malawi, Mozambique, Senegal). There was also variability in results across countries as exemplified by Burundi (OR: 3.71, 95% CI: 2.63-5.25) and Mozambique (OR: 2.79, 95% CI: 1.92-4.05) compared to Rwanda (OR: 0.83, 95% CI: 0.48-1.44) and Uganda (1.24, 95% CI: 0.96-1.61).

### Any antibiotic use

Only Rwanda and Uganda had significantly higher antibiotic use odds associated with diagnostic testing (Rwanda OR: 2.95, 95% CI: 1.82-4.79; Uganda OR: 1.37, 95% CI: 1.09-1.72) while Burundi had significantly lower antibiotic use odds for tested paediatric fevers compared to untested cases (OR: 0.53, 95% CI: 0.40-0.72).

### Sub-national results

Table 3 suggests differences in the effect of diagnostic testing on paediatric fever treatment within some countries by malaria risk, source of care and symptoms. There was no evidence of interactions for other investigated variables (season, age) due in part to insufficient power to detect such differences.

**Table 3:** **Sub-national differences in the effect of diagnostic testing on paediatric fever treatment in studied countries**

**Country**	**Outcome**	**Strata**	**n febrile under fives taken to any care**	**% with outcome (95% CI)**	**% with a diagnostic test (95% CI)**	**AOR (95% CI)**	**p value**
**Malaria risk**							
Benin	Any anti-malarial use	High risk	403	50.8 (45.3-56.3)	20.0 (16.1-24.4)	2.30 (1.10-4.82)	0.028
		Moderate risk	212	41.4 (32.9-50.5)	18.7 (11.9-28.1)	0.32 (0.08-1.29)	0.108
Burundi*	Any antibiotic use	Moderate risk	1,239	54.0 (49.8-58.2)	37.7 (33.8-41.6)	0.44 (0.32-0.60)	<0.001
		Low risk	125	57.3 (42.7-71.8)	37.1 (27.2-47.0)	6.75 (1.30-35.00)	0.023
Malawi	Any anti-malarial use	High risk	1,870	56.9 (53.8-60.1)	19.5 (16.8-22.2)	1.63 (1.26-2.11)	<0.001
		Moderate risk	2,361	53.2 (49.5-56.9)	22.8 (19.3-26.3)	1.12 (0.85-1.47)	0.410
Uganda	ACT use	High risk	1,730	46.1 (41.5-50.9)	26.9 (23.3-30.8)	0.95 (0.70-1.28)	0.736
		Moderate risk	625	51.6 (46.2-57.1)	34.7 (28.9-41.1)	0.67 (0.46-0.98)	0.040
	Any antibiotic use	High risk	1,730	36.6 (32.9-40.4)	26.9 (23.3-30.8)	1.24 (0.93-1.65)	0.146
		Moderate risk	625	33.0 (28.3-38.1)	34.7 (28.9-41.1)	1.68 (1.12-2.52)	0.012
**Source of care**							
Malawi	Any antibiotic use	Public	2,978	27.6 (25.2-30.0)	19.3 (17.2-21.4)	0.85 (0.67-1.09)	0.199
		Private	1,358	34.0 (30.2-37.8)	26.0 (21.7-30.4)	1.36 (0.95-1.96)	0.097
Rwanda*	Any anti-malarial use	Public	522	24.4 (20.8-28.4)	40.3 (35.8-44.8)	0.59 (0.31-1.14)	0.119
		Private	134	5.0 (2.3-10.8)	22.4 (15.6-31.1)	29.38 (2.25-383.63)	0.010
**Symptoms**							
Benin*	Any anti-malarial use	Fever alone	380	46.6 (40.9-52.5)	15.5 (11.8-20.1)	0.94 (0.39-2.29)	0.893
		Fever, cough, rapid breaths	239	50.5 (43.1-57.8)	27.2 (21.0-34.5)	5.48 (1.27-23.63)	0.022
Burkina Faso	Any anti-malarial use	Fever alone	1,253	48.5 (44.9-52.2)	6.9 (5.5-8.6)	2.03 (1.13-3.66)	0.018
		Fever, cough, rapid breaths	570	46.7 (42.0-51.5)	9.5 (6.9-12.8)	0.69 (0.34-1.41)	0.304
Malawi	ACT use	Fever alone	1,980	51.7 (48.4-54.9)	21.9 (19.3-24.5)	0.87 (0.66-1.13)	0.298
		Fever, cough, rapid breaths	2,355	42.2 (39.1-45.4)	21.0 (18.1-23.9)	1.40 (1.09-1.79)	0.008
	Any antibiotic use	Fever alone	1,980	18.2 (16.0-20.4)	21.9 (19.3-24.5)	1.46 (1.07-1.99)	0.017
		Fever, cough, rapid breaths	2,355	39.2 (36.2-42.2)	21.0 (18.1-23.9)	0.77 (0.60-1.00)	0.052
Senegal*	ACT use	Fever alone	527	5.6 (3.3-9.1)	15.1 (11.5-19.6)	75.42 (1.09-5212.78)	0.045
		Fever, cough, rapid breaths	747	5.5 (3.2-9.3)	14.8 (11.1-19.4)	1.69 (0.50-5.73)	0.400

If evidence of an interaction was found (0.05 level) between diagnostic testing and investigated variables (malaria risk, transmission season, age, symptoms, source of care) within a country, the final model was stratified accordingly and results presented above. CI = confidence interval; AOR = adjusted odds ratio. AORs are based on mixed-effects logistic regression models for specified strata in each country dataset adjusted for data clustering and confounding covariates (malaria endemicity; transmission season; public/private source; level of care; child’s age and sex; maternal age and education; residence; household wealth and size; health care access (money); health care access (distance); symptoms; health card).

*Some results should be interpreted with caution due to few observations and few positive outcomes.

### Malaria risk

In four countries (Benin, Burundi, Malawi, Uganda), data suggest higher-risk areas had higher malaria treatment odds associated with diagnostic testing compared to lower-risk areas, and the opposite for antibiotic use. In Uganda, moderate-risk areas had significantly reduced ACT use odds for tested compared to untested cases (OR: 0.67, 95% CI: 0.46-0.98) and significantly higher antibiotic use odds associated with testing (OR: 1.68, 95% CI: 1.12-2.52), while there was a negligible difference in high-risk areas. In Benin and Malawi, tested paediatric fevers in high-risk areas had significantly higher anti-malarial use odds compared to untested cases (Benin OR: 2.30, 95% CI: 1.10-4.82; Malawi OR: 1.63, 95% CI: 1.26-2.11), while this difference was negligible in moderate-risk areas. In Burundi, antibiotic treatment odds was significantly higher in low-risk areas for tested compared to untested cases (OR: 6.75, 95% CI: 1.30-35.00), although this strong effect should be interpreted with caution. Conversely, there was significantly lower antibiotic use odds associated with testing in moderate-risk settings (OR: 0.44, 95% CI: 0.32-0.60).

### Source of care

Attending private sources in Rwanda was associated with significantly higher anti-malarial treatment odds for tested paediatric fevers compared to untested ones (OR: 29.38, 95% CI: 2.25-383.63), although this strong effect should be interpreted with caution. Malawi also had slightly higher antibiotic treatment odds associated with testing in the private sector, but this result was non-significant.

### Symptoms

There was less consistency in sub-national results by reported symptoms. In Benin, tested children with fever and respiratory symptoms had significantly higher anti-malarial treatment odds compared to untested cases (OR: 5.48, 95% CI: 1.27-23.63), while there was no difference if fever alone was reported. In Malawi, reporting fever and respiratory symptoms was associated with 1.40 (95% CI: 1.09-1.79) times higher ACT use odds for tested cases compared to untested ones, with a negligible difference for children with fever alone. Conversely, in Burkina Faso, tested children having fever alone had significantly higher malaria treatment odds compared to untested cases (OR: 2.03, 95% CI: 1.13-3.66), while there was no difference if fever and respiratory symptoms were reported.

### Case studies

Case study summaries focus on describing relevant programme features or contextual factors derived from interviews and document reviews that could help understand quantitative results. Case studies for the six countries are presented in additional files (Additional file 3) and were used to inform the cross-case synthesis to identify common themes across countries and sub-national groups highlighted below (Table 4).

**Table 4:** **Descriptive typology of plausible explanations for quantitative results in six countries**

		**Rwanda**	**Uganda**	**Malawi**	**Benin**	**Mozambique**	**Burundi**
		**DHS 2010-2011**	**DHS 2011**	**DHS 2010**	**DHS 2011-2012**	**DHS 2011**	**DHS 2010-2011**
**Outcomes (see Table** **2** **)**	AOR (any anti-malarial use)	0.83 (0.48-1.44)	1.24 (0.96-1.61)	1.34 (1.11-1.61)	1.65 (0.92-2.98)	2.79 (1.92-4.05)	3.71 (2.63-5.25)
	AOR (ACT use)	0.88 (0.51-1.51)	0.84 (0.66-1.06)	1.12 (0.94-1.34)	1.96 (0.91-4.19)	3.54 (2.33-5.39)	2.78 (1.81-4.27)
	AOR (any antibiotic use)	2.95 (1.82-4.79)	1.37 (1.09-1.72)	1.00 (0.82-1.22)	1.15 (0.64-2.08)	1.01 (0.64-1.59)	0.53 (0.40-0.72)
**Available diagnostics and medicines**	National ACT scale-up initiated	Yes	Yes	Yes	Yes	Yes	Yes
	National RDT scale-up initiated	Yes	No	No	Yes	Yes	No
	Reported inconsistent RDT supplies	No	N/A	N/A	Mixed reports [9,44]	Yes	N/A
	Reported inconsistent ACT supplies	No	Yes	No [20]	Yes	Yes	Yes
	Diagnostics at community-level	Yes [61]	No	No	No	No	No
	Diagnostics at peripheral facilities	Yes	Yes*	No	Yes**	Yes**	No
	Diagnostics at hospitals	Yes	Yes	Yes	Yes	Yes	Yes
**Quality of care**	Diagnostic test adherence (% test-negative patients prescribed malaria treatment)	Perceived good	Poor [30%;48%] [12,39]	Poor [20%] [20]	Poor [38%] [9]	Perceived poor	Perceived poor
**Care-seeking behaviour**	Extensive use of informal private sector	No	Yes [62]	No	Yes	No	No
**Malaria epidemiology**	Malaria endemicity in 2010 [37]	Malaria-free to moderate-risk	Malaria-free to high-risk	Moderate to high-risk	Moderate to high-risk	Moderate to high-risk	Malaria-free to high-risk

Information summarizes case study discussions and references [41-44] unless otherwise noted. Reported percentages of test-negative patients prescribed malaria treatment refers to all patients and is plausibly higher for young children and in routine program conditions. Benin and Malawi results based on national-level facility surveys.

*In Uganda, microscopy is available at HC-III and higher-level facilities.

**RDT stock-outs will reduce availability of diagnostics at peripheral clinics.

**Available diagnostics and medicines** was emphasized by all respondents as a central issue affecting results.

*“Children getting tested are probably at locations that also have medicines, and those not tested likely have worse access to ACT. That’s a key issue.” (Benin case study).*

Only Rwanda, Benin and Mozambique had initiated wide-scale RDT deployment prior to surveys, and in Rwanda, CHWs used RDT. Data for remaining countries largely reflects testing by microscopy concentrated at referral hospitals, except Uganda where health centres have laboratory services. Weak supply systems could also reduce availability of diagnostics or medicines, which further concentrates supplies at hospitals even if RDT scale-up was previously initiated. In Uganda, microscopy services were offered at health centres but with inconsistent ACT supplies at these facilities around this time. Rwanda, in contrast, was described as having a strong logistics system for medicines and diagnostics.

**Quality of care** may also influence findings if there is poor test-based case management at sources with both diagnostics and medicines. Contemporaneous research in Benin, Malawi and Uganda indicated that about 20-50% of test-negative patients were prescribed malaria treatment [9,12,20,39] with plausibly worse adherence for young children [20] and in routine conditions [24]. It should be noted that only Benin and Malawi results are based on national-level facility surveys. In Mozambique and Burundi, test adherence practices were perceived as poor.

*“(In Mozambique) there was little experience with testing at peripheral facilities at this time (with the lack of widely available RDT stocks), and poor case management practices in general.” (Mozambique case study)*.

In Rwanda, adherence practices were seen as comparatively good although effect differences across public/private sources were attributed to poor practices at the latter (Table 3).

*“The private sector is small and more developed in Kigali. Clients attending these facilities often expect or demand certain medicines and some health providers want to maintain client satisfaction.” (Rwanda case study).*

**Care-seeking behaviour** was also described as influencing quantitative results, particularly in countries where drug shops are commonly used to treat sick children or where there is difficult access to formal care.

*“In Benin care-seeking often goes: child has fever, taken to shop, gets medicines, doesn’t get better, goes to facility (where finally tested).” (Benin case study).*

Caregivers may self-treat sick children at home or in communities either before or after visiting a facility where diagnostic testing occurs. This could result in over-treatment associated with diagnostic testing at the population level that is unrelated to the quality of care provided at facilities although attempts were made to account for this issue in the analysis (see Methods). In addition, this practice may delay visiting formal providers such that children are more severely ill once they reach facilities with diagnostic services, which further increases treatment likelihood. Illness severity was also put forward to potentially explain effect differences found by child symptoms in Malawi and Benin (Table 3). Delayed care-seeking practices are also an important issue for countries where there is simply difficult access to formal care, particularly if supplies are concentrated at hospitals.

“*(In Burundi) microscopy is basically in hospitals and healthcare access is difficult - remote, hilly communities. These results really show poor access to care.” (Burundi case study).*

**Malaria epidemiology** was also noted to affect results since the likelihood of malaria infection plausibly influences diagnosis and treatment practices. Four studied countries showed effect differences across malaria strata, which may further support this theory (Table 3).

*“There are three main reasons I can think of that could explain (Mozambique) results: (1) high malaria prevalence in certain areas even in the dry season (2) poor case management practices (3) access to testing and care (rural, bad infrastructure, hard to get tested and treated).” (Mozambique case study).*

## Discussion

Overall, findings indicate variability in the effect of diagnostic testing on paediatric fever treatment at the population level across studied countries in 2010–2012, and no country demonstrated significant ACT use reductions associated with testing as hypothesized. Four common themes emerged to explain varying results: available diagnostics and medicines; quality of care; care-seeking behaviour; and, malaria epidemiology.

Indeed, the study hypothesis is implicitly grounded in an assumption that all febrile children have similar access to medicines irrespective of testing status, and that untested paediatric fevers are presumptively given malaria treatment while only a sub-set of tested ones are treated (e.g., positive cases). There are numerous reasons why this hypothesis may not hold in routine programme conditions, particularly at the outset of new guidelines, and that significantly different results may be observed across countries.

One important issue is that countries included in this analysis had relatively low diagnostic test coverage at the population level given the early assessment. This low coverage plausibly indicates that diagnostic services remained concentrated at higher-level facilities that generally have better medicine stocks and more severely ill patients, both of which increase treatment likelihood. Indeed, *available diagnostics and medicines* was a main theme identified in this study to explain varying results (see Table 4). This is consistent with other research suggesting that access to care is the greatest contributor to reduced systems effectiveness for malaria case management in Zambia [47].

This theme was cited as a main explanation for results across all case study countries. In Mozambique and Burundi, for example, diagnostic services were concentrated at hospitals at the time of survey fieldwork given the lack of RDT scale-up (e.g., Burundi) or widespread RDT stock-outs (e.g., Mozambique). At the same time, both of these countries experienced ACT shortages at peripheral clinics such that febrile children attending locations with diagnostic tests probably had better access to medicines. General poor access to formal care and low use of informal providers (e.g., drug shops) in these countries may have further compounded the vast under-treatment of untested paediatric fevers. Yet, in Benin and Malawi, diagnostic services seemed less a marker of access to medicines. In Benin, there were mixed reports about RDT and ACT availability at peripheral facilities around this time [9,44], and drug shops without diagnostic services are widely used to obtain medicines. In Malawi, a national facility survey indicated ACT and other medicines were commonly available at peripheral clinics that lacked diagnostic services [20]. In Uganda and Rwanda, in contrast, diagnostic services were more widely available at this time with microscopy at Ugandan health centres and RDT at the community level in Rwanda [44]. In Uganda, however, reported ACT stock-outs at health centres with microscopy services could also have impacted its results.

As countries move towards universal test coverage, it is, therefore, reasonable to expect that expanded access to testing and care could reduce the strong association between diagnosis and malaria treatment found in this analysis. Yet, other factors may still influence this relationship at the population level even if universal test coverage is achieved. Other main themes identified in this study included *quality of care*, *care-seeking behaviour* and *malaria epidemiology*, which have also been described elsewhere [6,7,25,48,49]. Finally, Rwanda and Uganda results also suggest that reductions in malaria treatment associated with testing may be met with increased antibiotic use, which has been documented in facility-based research from Tanzania [13,15]. Given strong concerns about irrational antibiotic prescription habits [50], this finding merits investigation in future facility-based adherence studies as well.

From a programme perspective, countries could potentially improve population-level results, as exemplified by Rwanda, largely by expanding access to testing and care and improving quality case management. Findings also suggest the impact of diagnostic-scale up on treatment decisions may not be straightforward in routine programme conditions given such issues as access to formal care, treatment-seeking behaviour or supply stock-outs. For example, in settings where there is poor access to formal care or where the informal sector is widely used, diagnostic services may need to be extended through integrated community case management approaches [51]. This could not only improve access to testing and care, but also help ensure that non-severe febrile illnesses are at least as likely to get tested as severe cases. Indeed, diagnosis of less severe cases is arguably more informative given the overlap of initial malaria symptoms with other illnesses [52], the critical need to reduce delays in appropriate fever management [53], and the plausible better test adherence for less severe patients as hypothesized in case studies.

This study is the first to use routine national surveys to examine the effect of diagnostic testing on paediatric fever treatment at the population level in a standardized manner across multiple countries and among key sub-national groups. As part of a mixed-methods approach, a typology to describe the complex phenomena that could drive results at the population level is presented, which draws on similar evaluation methods in health services research [54]. The inclusion of multiple countries with contrasting results and common explanatory themes lends support to the external validity of conclusions (known as theoretical replication) and the potential application of this typology to other contexts [27]. In fact, future quantitative research using these datasets could potentially employ these themes to predict the relationship between testing and treatment at the population level in other countries or over time as RDT scale-up continues. This typology could also serve as a standard framework to assess programmes in routine conditions, and may be particularly informative for countries without more robust evidence. Another benefit of DHS analyses is to help identify countries (e.g., Mozambique) or issues (e.g., malaria risk) where further facility-based research or programme attention may be warranted.

There are notable data limitations. First, data in this analysis are from 2010–2012 and many studied countries began wide-scale RDT deployment after these surveys were conducted [44]. Recent research shows improved test-based treatment practices over time [5,8] and new methods hold promise to further improve case management [55,56]. Countries could show different results in future analyses, and this assessment could be repeated once new datasets become available. Second, surveys do not record a child’s test result and analysis of (in)appropriate case management is not possible using these data. Without the test result, quantitative findings alone are quite difficult to interpret. The mixed-methods approach was developed to help understand results but provides only a set of plausible explanations as a basis for further quantitative research. Third, analyses of observational data are prone to residual confounding issues and two key variables are not directly measured in surveys that confound the exposure-outcome relationship: illness severity and ‘well-stocked’ facility attendance. This analysis was limited to DHS datasets that contained specific proxy variables for these issues, but the relationship likely remains confounded as highlighted in case studies. Finally, caregiver recall of medicines given to sick children has shown mixed results in studies [57,58]. There may be worse recall among poor, rural or less educated mothers [59], which could over-estimate effect differences across countries if there are systematic differences in populations with access to testing and treatment.

## Conclusions

This paper is the first to quantify the influence of diagnostic testing on paediatric fever treatment at the population level in a standardized manner across multiple countries, and is presented as part of a mixed-methods approach to explain country results. Significant country variation was found in the effect of diagnostic testing on paediatric fever treatment at the population-level, and qualitative results suggest the impact of diagnostic-scale up on treatment decisions may not be straightforward in routine programme conditions given contextual issues such as access to care, treatment-seeking behaviour or supply stock-outs. Despite data limitations, quantitative results could help identify countries (e.g., Mozambique) or issues (e.g., malaria risk) where facility-based research or programme attention may be warranted. The mixed-methods approach brings together population- and facility-based data, programme documents, research studies, and expert opinions and could potentially be used to assess routine programmes across countries or over time to help fill critical evidence gaps.

## Additional files

Additional file 1:
**Characteristics of febrile children under five years taken to any care and receiving medicines in 12 countries.** Point estimates tabulated using sample weights pre-specified in datasets. Standard error estimation accounted for data clustering in survey designs. Additional file 2:
**Effect of diagnostic testing and other covariates on medicines used by febrile children under five years taken to any care in 12 countries.** CI = confidence interval; AOR = adjusted odds ratio. AORs based on mixed-effects logistic regression models adjusted for data clustering and all listed covariates. Additional file 3:
**Country case studies for Benin, Burundi, Malawi, Mozambique, Rwanda, Uganda.**

## Abbreviations

ACT: Artemisinin-based combination therapy
DHS: Demographic and Health Surveys
RDT: Rapid diagnostic test
*Pf*PR_2–10_: *Plasmodium falciparum* parasite rate in two to ten years olds
WHO: World Health Organization
