# Supplementary material for: Effect of diagnostic testing on medicines used by febrile children less than five years in 12 malaria-endemic African countries: a mixed-methods study
Source: Malar J. 2015 May 10;14:194. doi: 10.1186/s12936-015-0709-0 (PMC4432948; doi:10.1186/s12936-015-0709-0)
Supplement: Additional file 1: — Characteristics of febrile children under five years taken to any care and receiving medicines in 12 countries. Point estimates tabulated using sample weights pre-specified in datasets. Standard error estimation accounted for data clustering in survey designs. [file 12936_2015_709_MOESM1_ESM.docx]

**Additional File 1: Characteristics of febrile children less than five years taken to any care and receiving medicines in 12 countries**

| **Benin DHS 2011-2012** |  | **N febrile under-fives taken to any care** | **Percent receiving any anti-malarial** | **95% CI** | | **Percent receiving ACT** | **95% CI** | | **Percent receiving any antibiotic** | **95% CI** | |
| --- | --- | --- | --- | --- | --- | --- | --- | --- | --- | --- | --- |
|  |  |  |  |  |  |  |  |  |  |  |  |
| **Diagnostic test use** | No | 492 | 44.8 | 39.8 | 49.7 | 16.0 | 12.3 | 19.7 | 27.9 | 23.2 | 32.5 |
|  | Yes | 124 | 59.9 | 48.3 | 71.5 | 22.1 | 14.0 | 30.3 | 30.8 | 21.3 | 40.2 |
| **Malaria endemicity** | Malaria-free | 0 | - | - | - | - | - | - | - | - | - |
|  | Unstable risk | 0 | - | - | - | - | - | - | - | - | - |
|  | Low risk (*Pf*PR_2–10_ <5%) | 0 | - | - | - | - | - | - | - | - | - |
|  | Moderate risk (*Pf*PR_2–10_ 5-40%) | 212 | 41.4 | 32.6 | 50.3 | 10.9 | 5.3 | 16.4 | 36.2 | 28.1 | 44.3 |
|  | High risk (*Pf*PR_2–10_ >40%) | 403 | 50.8 | 45.3 | 56.4 | 21.0 | 16.7 | 25.3 | 24.7 | 19.9 | 29.4 |
| **Source (public/private)** | Public | 336 | 60.1 | 54.2 | 66.0 | 24.4 | 19.6 | 29.2 | 38.0 | 32.2 | 43.8 |
|  | Private | 284 | 33.7 | 27.4 | 40.0 | 9.4 | 5.9 | 12.9 | 17.4 | 12.2 | 22.5 |
| **Source (level)** | Hospital | 133 | 51.7 | 42.0 | 61.5 | 16.1 | 9.8 | 22.5 | 39.1 | 29.9 | 48.3 |
|  | Non-hospital formal medical | 250 | 59.1 | 52.3 | 66.0 | 25.5 | 19.8 | 31.2 | 38.9 | 32.3 | 45.6 |
|  | Community health worker | 17 | 56.6 | 33.4 | 79.7 | 16.9 | 1.8 | 32.0 | 13.7 | -1.3 | 28.7 |
|  | Pharmacy | 44 | 46.5 | 30.4 | 62.6 | 17.9 | 6.7 | 29.2 | 24.7 | 11.9 | 37.5 |
|  | Other | 177 | 29.1 | 22.1 | 36.1 | 7.3 | 3.3 | 11.2 | 8.3 | 2.8 | 13.9 |
| **Child's age (months)** | 0 - 5 | 61 | 32.4 | 19.1 | 45.8 | 8.0 | 1.0 | 14.9 | 36.5 | 22.2 | 50.8 |
|  | 6 - 11 | 96 | 43.7 | 32.9 | 54.4 | 17.5 | 10.1 | 24.9 | 41.2 | 30.6 | 51.8 |
|  | 12 - 23 | 162 | 52.1 | 43.8 | 60.3 | 17.2 | 11.1 | 23.4 | 29.0 | 21.9 | 36.2 |
|  | 24 - 35 | 126 | 49.9 | 40.3 | 59.5 | 16.2 | 9.8 | 22.6 | 24.0 | 16.1 | 31.8 |
|  | 36 - 47 | 86 | 48.5 | 36.6 | 60.5 | 27.8 | 17.2 | 38.4 | 25.4 | 15.2 | 35.7 |
|  | 48 - 59 | 89 | 53.0 | 42.2 | 63.7 | 16.5 | 8.6 | 24.3 | 18.1 | 10.1 | 26.0 |
| **Child's sex** | Male | 316 | 47.0 | 40.5 | 53.6 | 18.3 | 13.3 | 23.3 | 29.9 | 24.1 | 35.7 |
|  | Female | 304 | 49.1 | 42.5 | 55.6 | 16.7 | 11.9 | 21.4 | 27.1 | 21.7 | 32.6 |
| **Maternal age (years)** | 15 -24 | 150 | 48.9 | 40.5 | 57.4 | 17.6 | 11.4 | 23.8 | 33.3 | 24.7 | 41.9 |
|  | 25 - 29 | 183 | 49.5 | 41.0 | 57.9 | 16.8 | 11.2 | 22.5 | 28.5 | 21.2 | 35.8 |
|  | 30 - 34 | 156 | 42.8 | 33.6 | 52.0 | 18.0 | 11.6 | 24.5 | 30.5 | 22.2 | 38.7 |
|  | 35 - 39 | 84 | 48.0 | 36.4 | 59.7 | 14.9 | 7.2 | 22.5 | 25.7 | 16.3 | 35.2 |
|  | 40 - 49 | 46 | 57.1 | 43.0 | 71.2 | 22.9 | 9.9 | 36.0 | 12.2 | 3.2 | 21.2 |
| **Maternal education** | No education | 393 | 46.7 | 40.9 | 52.5 | 16.7 | 12.6 | 20.8 | 25.0 | 20.3 | 29.7 |
|  | Primary | 146 | 50.8 | 41.6 | 60.0 | 18.9 | 11.6 | 26.2 | 33.1 | 24.4 | 41.8 |
|  | Secondary or higher | 81 | 49.6 | 37.8 | 61.4 | 18.9 | 10.5 | 27.3 | 37.6 | 26.4 | 48.9 |
| **Residence** | Urban | 308 | 48.1 | 40.5 | 55.6 | 16.5 | 11.4 | 21.6 | 33.7 | 27.1 | 40.3 |
|  | Rural | 312 | 48.0 | 41.9 | 54.0 | 18.5 | 13.8 | 23.3 | 23.5 | 18.5 | 28.5 |
| **Household wealth** | Poorest | 74 | 47.6 | 35.7 | 59.6 | 11.7 | 4.8 | 18.6 | 14.8 | 6.9 | 22.8 |
|  | Second | 119 | 54.7 | 45.2 | 64.3 | 16.2 | 9.5 | 22.9 | 18.8 | 11.2 | 26.4 |
|  | Middle | 120 | 51.5 | 42.2 | 60.9 | 26.6 | 18.0 | 35.3 | 29.3 | 20.7 | 38.0 |
|  | Fourth | 135 | 40.1 | 31.8 | 48.4 | 17.3 | 11.0 | 23.5 | 39.8 | 30.2 | 49.4 |
|  | Least poor | 172 | 47.3 | 36.4 | 58.2 | 14.7 | 7.6 | 21.9 | 31.8 | 23.3 | 40.3 |
| **Household members** | 1-4 members | 170 | 43.9 | 35.8 | 52.0 | 14.4 | 9.1 | 19.8 | 32.6 | 25.0 | 40.3 |
|  | 5-8 members | 332 | 46.9 | 40.5 | 53.3 | 17.2 | 12.8 | 21.6 | 26.4 | 21.0 | 31.9 |
|  | 9-12 members | 87 | 55.8 | 45.3 | 66.3 | 26.9 | 16.5 | 37.2 | 28.3 | 17.5 | 39.0 |
|  | 13 or more members | 32 | 60.8 | 40.6 | 81.1 | 11.4 | 0.5 | 22.3 | 29.9 | 10.1 | 49.6 |
| **Health care access (money)** | Big problem | 364 | 46.6 | 40.6 | 52.6 | 16.7 | 12.8 | 20.6 | 30.0 | 24.7 | 35.3 |
|  | Not a big problem | 256 | 50.1 | 42.6 | 57.6 | 18.7 | 12.8 | 24.5 | 26.5 | 20.4 | 32.5 |
| **Health care access (distance)** | Big problem | 233 | 46.5 | 39.2 | 53.8 | 15.2 | 10.6 | 19.9 | 28.8 | 22.2 | 35.5 |
|  | Not a big problem | 387 | 48.9 | 42.9 | 55.0 | 18.9 | 14.3 | 23.5 | 28.4 | 23.0 | 33.8 |
| **Symptoms** | Fever alone | 380 | 46.6 | 40.8 | 52.4 | 19.4 | 15.1 | 23.7 | 23.7 | 18.4 | 29.0 |
|  | Fever, cough | 142 | 43.7 | 34.7 | 52.6 | 17.7 | 10.6 | 24.8 | 38.9 | 29.9 | 47.9 |
|  | Fever, cough, rapid breaths | 98 | 60.3 | 48.8 | 71.8 | 10.2 | 4.4 | 15.9 | 32.6 | 22.5 | 42.6 |
| **Malaria transmission season** | Off-peak | 466 | 49.4 | 44.3 | 54.5 | 19.0 | 15.1 | 22.8 | 26.4 | 21.8 | 31.0 |
|  | Peak | 149 | 41.9 | 30.5 | 53.3 | 12.9 | 5.5 | 20.4 | 35.6 | 26.5 | 44.7 |
| **Health card** | No (never had or lost) | 55 | 31.4 | 17.4 | 45.4 | 12.6 | 1.4 | 23.7 | 28.7 | 14.3 | 43.0 |
|  | Yes (seen or reported) | 565 | 49.7 | 44.8 | 54.5 | 18.0 | 14.4 | 21.6 | 28.5 | 24.2 | 32.9 |

Point estimates tabulated using sample weights pre-specified in datasets. Standard error estimation accounted for data clustering in survey designs.

**Burkina Faso DHS 2010-2011**

|  |  | **N febrile under-fives taken to any care** | **Percent receiving any anti-malarial** | **95% CI** | | **Percent receiving ACT** | **95% CI** | | **Percent receiving any antibiotic** | **95% CI** | |
| --- | --- | --- | --- | --- | --- | --- | --- | --- | --- | --- | --- |
|  |  |  |  |  |  |  |  |  |  |  |  |
| **Diagnostic test use** | No | 1,671 | 46.8 | 43.6 | 50.1 | 12.1 | 10.1 | 14.1 | 43.8 | 40.6 | 47.0 |
|  | Yes | 140 | 61.1 | 51.9 | 70.3 | 16.9 | 10.5 | 23.3 | 44.2 | 35.0 | 53.4 |
| **Malaria endemicity** | Malaria-free | 0 | - | - | - | - | - | - | - | - | - |
|  | Unstable risk | 0 | - | - | - | - | - | - | - | - | - |
|  | Low risk (*Pf*PR_2–10_ <5%) | 0 | - | - | - | - | - | - | - | - | - |
|  | Moderate risk (*Pf*PR_2–10_ 5-40%) | 0 | - | - | - | - | - | - | - | - | - |
|  | High risk (*Pf*PR_2–10_ >40%) | 1,727 | 48.4 | 45.1 | 51.6 | 12.6 | 10.7 | 14.5 | 43.7 | 40.5 | 46.9 |
| **Source (public/private)** | Public | 1,453 | 56.8 | 53.5 | 60.2 | 14.8 | 12.6 | 17.1 | 51.2 | 47.7 | 54.7 |
|  | Private | 371 | 13.2 | 9.2 | 17.2 | 3.3 | 1.2 | 5.3 | 15.8 | 11.0 | 20.7 |
| **Source (level)** | Hospital | 251 | 63.0 | 55.6 | 70.5 | 16.9 | 11.1 | 22.6 | 46.1 | 37.8 | 54.4 |
|  | Non-hospital formal medical | 1,223 | 55.3 | 51.6 | 59.0 | 14.5 | 12.1 | 16.9 | 52.2 | 48.5 | 55.9 |
|  | Community health worker | 26 | 17.3 | 1.1 | 33.5 | 2.4 | -2.3 | 7.0 | 37.5 | 11.9 | 63.0 |
|  | Pharmacy | 57 | 25.0 | 13.5 | 36.6 | 7.7 | 0.9 | 14.5 | 34.8 | 21.5 | 48.0 |
|  | Other | 266 | 8.2 | 4.7 | 11.7 | 1.0 | -0.3 | 2.3 | 7.2 | 2.6 | 11.7 |
| **Child's age (months)** | 0 - 5 | 116 | 34.6 | 25.2 | 44.0 | 10.5 | 4.2 | 16.8 | 53.1 | 43.5 | 62.7 |
|  | 6 - 11 | 297 | 44.0 | 38.2 | 49.8 | 13.4 | 9.3 | 17.6 | 47.2 | 41.3 | 53.2 |
|  | 12 - 23 | 554 | 46.7 | 41.9 | 51.5 | 12.8 | 9.4 | 16.1 | 45.4 | 40.5 | 50.2 |
|  | 24 - 35 | 409 | 48.7 | 42.6 | 54.9 | 11.2 | 7.8 | 14.6 | 48.3 | 42.9 | 53.8 |
|  | 36 - 47 | 275 | 56.4 | 50.1 | 62.8 | 12.7 | 8.6 | 16.8 | 35.1 | 28.8 | 41.4 |
|  | 48 - 59 | 174 | 52.6 | 44.3 | 60.8 | 14.0 | 8.6 | 19.4 | 32.1 | 24.4 | 39.8 |
| **Child's sex** | Male | 960 | 48.2 | 44.5 | 52.0 | 13.7 | 11.1 | 16.2 | 45.8 | 41.8 | 49.7 |
|  | Female | 863 | 47.7 | 43.6 | 51.7 | 11.2 | 8.7 | 13.7 | 42.1 | 38.3 | 45.9 |
| **Maternal age (years)** | 15 -24 | 579 | 51.0 | 46.0 | 56.0 | 16.4 | 12.8 | 20.1 | 44.5 | 39.8 | 49.2 |
|  | 25 - 29 | 464 | 42.7 | 37.7 | 47.6 | 11.0 | 8.0 | 14.0 | 47.1 | 41.5 | 52.7 |
|  | 30 - 34 | 357 | 48.0 | 41.9 | 54.1 | 11.0 | 7.5 | 14.4 | 39.4 | 33.6 | 45.3 |
|  | 35 - 39 | 267 | 52.0 | 44.7 | 59.3 | 9.3 | 5.1 | 13.5 | 43.9 | 37.9 | 49.8 |
|  | 40 - 49 | 156 | 45.3 | 36.0 | 54.7 | 11.2 | 6.0 | 16.5 | 43.7 | 35.2 | 52.2 |
| **Maternal education** | No education | 1,422 | 45.6 | 42.2 | 49.0 | 11.2 | 9.2 | 13.3 | 43.8 | 40.4 | 47.2 |
|  | Primary | 262 | 56.4 | 50.3 | 62.6 | 14.4 | 10.1 | 18.7 | 43.8 | 36.9 | 50.7 |
|  | Secondary or higher | 138 | 56.8 | 46.1 | 67.4 | 21.9 | 13.6 | 30.3 | 46.0 | 36.6 | 55.3 |
| **Residence** | Urban | 390 | 54.7 | 48.8 | 60.5 | 17.1 | 12.6 | 21.6 | 42.8 | 36.8 | 48.8 |
|  | Rural | 1,433 | 46.2 | 42.5 | 49.8 | 11.2 | 9.2 | 13.3 | 44.4 | 40.8 | 47.9 |
| **Household wealth** | Poorest | 253 | 47.1 | 39.6 | 54.5 | 11.5 | 7.3 | 15.7 | 34.0 | 27.1 | 40.9 |
|  | Second | 333 | 34.6 | 29.0 | 40.2 | 8.1 | 4.9 | 11.3 | 45.3 | 38.6 | 52.1 |
|  | Middle | 439 | 46.4 | 40.5 | 52.3 | 9.1 | 6.1 | 12.0 | 43.8 | 37.9 | 49.7 |
|  | Fourth | 440 | 50.5 | 44.4 | 56.5 | 16.0 | 12.0 | 20.0 | 47.9 | 42.6 | 53.1 |
|  | Least poor | 359 | 59.9 | 52.9 | 66.8 | 17.2 | 12.4 | 21.9 | 45.4 | 38.8 | 52.0 |
| **Household members** | 1-4 members | 462 | 48.6 | 43.4 | 53.7 | 13.0 | 9.7 | 16.3 | 48.1 | 42.8 | 53.5 |
|  | 5-8 members | 783 | 50.9 | 46.5 | 55.3 | 14.1 | 11.2 | 17.0 | 41.1 | 36.9 | 45.4 |
|  | 9-12 members | 379 | 42.8 | 36.4 | 49.2 | 10.3 | 6.8 | 13.9 | 41.8 | 35.9 | 47.8 |
|  | 13 or more members | 199 | 44.9 | 36.5 | 53.2 | 9.1 | 4.9 | 13.3 | 50.0 | 41.3 | 58.8 |
| **Health care access (money)** | Big problem | 1,323 | 48.1 | 44.4 | 51.9 | 13.4 | 11.1 | 15.7 | 41.4 | 37.7 | 45.1 |
|  | Not a big problem | 500 | 47.6 | 42.4 | 52.8 | 10.2 | 7.1 | 13.2 | 50.9 | 45.7 | 56.1 |
| **Health care access (distance)** | Big problem | 869 | 45.8 | 41.5 | 50.1 | 13.1 | 10.1 | 16.0 | 44.0 | 39.7 | 48.3 |
|  | Not a big problem | 954 | 50.0 | 45.8 | 54.1 | 12.0 | 9.7 | 14.3 | 44.0 | 40.3 | 47.8 |
| **Symptoms** | Fever alone | 1,253 | 48.5 | 44.9 | 52.2 | 13.4 | 11.1 | 15.7 | 37.9 | 34.3 | 41.5 |
|  | Fever, cough | 330 | 48.1 | 42.3 | 53.9 | 10.4 | 6.7 | 14.1 | 54.9 | 48.8 | 60.9 |
|  | Fever, cough, rapid breaths | 241 | 44.9 | 37.5 | 52.3 | 10.6 | 6.8 | 14.4 | 61.1 | 54.1 | 68.2 |
| **Malaria transmission season** | Off-peak | 386 | 43.3 | 36.6 | 50.0 | 11.5 | 7.6 | 15.4 | 55.6 | 48.7 | 62.4 |
|  | Peak | 1,341 | 49.8 | 46.1 | 53.5 | 12.9 | 10.7 | 15.1 | 40.3 | 36.7 | 43.9 |
| **Health card** | No (never had or lost) | 176 | 38.5 | 29.8 | 47.1 | 9.9 | 5.6 | 14.1 | 35.9 | 27.9 | 44.0 |
|  | Yes (seen or reported) | 1,645 | 48.9 | 45.8 | 52.1 | 12.8 | 10.8 | 14.8 | 44.8 | 41.6 | 48.1 |

Point estimates tabulated using sample weights pre-specified in datasets. Standard error estimation accounted for data clustering in survey designs.

**Burundi DHS 2010-2011**

|  |  | **N febrile under-fives taken to any care** | **Percent receiving any anti-malarial** | **95% CI** | | **Percent receiving ACT** | **95% CI** | | **Percent receiving any antibiotic** | **95% CI** | |
| --- | --- | --- | --- | --- | --- | --- | --- | --- | --- | --- | --- |
|  |  |  |  |  |  |  |  |  |  |  |  |
| **Diagnostic test use** | No | 895 | 18.8 | 15.7 | 21.9 | 14.1 | 11.1 | 17.0 | 58.1 | 53.9 | 62.2 |
|  | Yes | 528 | 38.3 | 32.5 | 44.0 | 24.9 | 19.3 | 30.6 | 48.8 | 42.7 | 54.9 |
| **Malaria endemicity** | Malaria-free | 59 | 3.8 | -0.1 | 7.8 | - | - | - | 61.1 | 43.3 | 78.8 |
|  | Unstable risk | 0 | - | - | - | - | - | - | - | - | - |
|  | Low risk (*Pf*PR_2–10_ <5%) | 125 | 16.4 | 4.7 | 28.1 | 7.2 | 0.1 | 14.3 | 57.3 | 42.7 | 71.8 |
|  | Moderate risk (*Pf*PR_2–10_ 5-40%) | 1,239 | 27.9 | 24.5 | 31.2 | 20.1 | 16.9 | 23.3 | 54.0 | 49.8 | 58.2 |
|  | High risk (*Pf*PR_2–10_ >40%) | 9 | 28.5 | -20.1 | 77.0 | - | - | - | 62.0 | 11.5 | 112.6 |
| **Source (public/private)** | Public | 1,242 | 26.8 | 23.4 | 30.2 | 18.9 | 15.8 | 22.0 | 55.1 | 50.9 | 59.4 |
|  | Private | 189 | 20.0 | 12.6 | 27.3 | 12.1 | 5.9 | 18.3 | 51.1 | 43.0 | 59.2 |
| **Source (level)** | Hospital | 208 | 25.5 | 18.3 | 32.6 | 13.3 | 7.4 | 19.2 | 57.7 | 49.9 | 65.4 |
|  | Non-hospital formal medical | 1,100 | 26.6 | 23.0 | 30.2 | 19.3 | 16.0 | 22.5 | 55.6 | 51.0 | 60.1 |
|  | Community health worker | 26 | 29.9 | 0.9 | 58.9 | 16.0 | -3.6 | 35.6 | 44.4 | 18.9 | 69.9 |
|  | Pharmacy | 50 | 10.6 | -1.4 | 22.6 | 6.0 | -1.4 | 13.4 | 38.7 | 23.2 | 54.2 |
|  | Other | 48 | 25.7 | 7.5 | 43.8 | 23.9 | 5.4 | 42.4 | 41.7 | 25.9 | 57.5 |
| **Child's age (months)** | 0 - 5 | 126 | 4.9 | 0.0 | 9.8 | 3.1 | -1.2 | 7.4 | 68.8 | 59.3 | 78.3 |
|  | 6 - 11 | 224 | 9.8 | 5.6 | 14.1 | 4.8 | 1.7 | 7.9 | 64.7 | 56.8 | 72.7 |
|  | 12 - 23 | 374 | 23.3 | 18.4 | 28.1 | 16.4 | 11.9 | 20.8 | 59.1 | 52.8 | 65.4 |
|  | 24 - 35 | 310 | 40.6 | 33.4 | 47.7 | 30.5 | 24.3 | 36.7 | 48.7 | 41.7 | 55.8 |
|  | 36 - 47 | 238 | 29.4 | 22.7 | 36.2 | 21.1 | 15.1 | 27.0 | 44.8 | 37.4 | 52.2 |
|  | 48 - 59 | 160 | 37.4 | 28.5 | 46.3 | 23.4 | 15.4 | 31.4 | 44.9 | 35.4 | 54.4 |
| **Child's sex** | Male | 736 | 28.4 | 24.1 | 32.8 | 20.2 | 16.2 | 24.1 | 51.7 | 46.8 | 56.5 |
|  | Female | 696 | 23.2 | 19.5 | 27.0 | 15.7 | 12.3 | 19.2 | 57.7 | 52.8 | 62.6 |
| **Maternal age (years)** | 15 -24 | 367 | 27.5 | 21.4 | 33.6 | 17.4 | 11.6 | 23.2 | 56.1 | 49.7 | 62.5 |
|  | 25 - 29 | 453 | 25.3 | 20.0 | 30.7 | 19.6 | 14.5 | 24.7 | 54.4 | 48.2 | 60.6 |
|  | 30 - 34 | 265 | 26.7 | 19.8 | 33.6 | 18.7 | 12.5 | 24.8 | 56.6 | 48.8 | 64.3 |
|  | 35 - 39 | 212 | 21.1 | 14.9 | 27.4 | 14.1 | 8.5 | 19.8 | 53.9 | 45.7 | 62.1 |
|  | 40 - 49 | 135 | 29.3 | 20.0 | 38.7 | 19.2 | 10.7 | 27.6 | 48.4 | 37.5 | 59.4 |
| **Maternal education** | No education | 737 | 25.4 | 21.6 | 29.2 | 18.1 | 14.6 | 21.7 | 50.9 | 45.8 | 56.0 |
|  | Primary | 609 | 27.3 | 22.8 | 31.8 | 18.2 | 14.2 | 22.3 | 57.2 | 51.8 | 62.7 |
|  | Secondary or higher | 86 | 20.2 | 10.0 | 30.3 | 15.4 | 5.5 | 25.4 | 67.5 | 55.4 | 79.6 |
| **Residence** | Urban | 98 | 16.4 | 10.9 | 21.9 | 7.3 | 2.5 | 12.1 | 64.2 | 57.7 | 70.8 |
|  | Rural | 1,334 | 26.6 | 23.3 | 29.9 | 18.8 | 15.8 | 21.8 | 53.9 | 49.8 | 58.1 |
| **Household wealth** | Poorest | 306 | 27.2 | 20.5 | 33.8 | 18.4 | 12.4 | 24.4 | 48.1 | 40.3 | 55.9 |
|  | Second | 328 | 26.3 | 19.9 | 32.6 | 20.3 | 14.9 | 25.6 | 55.6 | 48.1 | 63.2 |
|  | Middle | 300 | 27.6 | 21.0 | 34.3 | 18.7 | 12.5 | 24.9 | 59.0 | 51.1 | 67.0 |
|  | Fourth | 258 | 28.1 | 21.3 | 34.8 | 19.0 | 12.5 | 25.4 | 54.0 | 46.9 | 61.2 |
|  | Least poor | 240 | 19.3 | 13.9 | 24.7 | 12.6 | 7.4 | 17.7 | 56.7 | 49.0 | 64.3 |
| **Household members** | 1-4 members | 496 | 26.8 | 21.5 | 32.2 | 18.5 | 13.7 | 23.3 | 56.4 | 50.3 | 62.5 |
|  | 5-8 members | 792 | 24.4 | 20.7 | 28.1 | 17.1 | 13.7 | 20.6 | 54.3 | 49.5 | 59.1 |
|  | 9-12 members | 133 | 30.9 | 20.6 | 41.2 | 22.0 | 11.2 | 32.8 | 48.6 | 38.9 | 58.3 |
|  | 13 or more members | 11 | 29.8 | -1.7 | 61.3 | 11.6 | -8.5 | 31.8 | 70.2 | 38.7 | 101.7 |
| **Health care access (money)** | Big problem | 1,070 | 25.8 | 22.2 | 29.5 | 18.1 | 14.8 | 21.4 | 53.2 | 48.8 | 57.6 |
|  | Not a big problem | 362 | 26.2 | 20.4 | 31.9 | 17.8 | 12.4 | 23.1 | 58.7 | 52.7 | 64.7 |
| **Health care access (distance)** | Big problem | 726 | 27.2 | 22.8 | 31.6 | 18.0 | 14.2 | 21.8 | 54.1 | 48.8 | 59.4 |
|  | Not a big problem | 706 | 24.5 | 20.2 | 28.8 | 18.1 | 14.1 | 22.0 | 55.1 | 50.2 | 60.0 |
| **Symptoms** | Fever alone | 487 | 40.4 | 34.3 | 46.4 | 29.6 | 24.0 | 35.1 | 37.4 | 31.8 | 42.9 |
|  | Fever, cough | 99 | 20.5 | 12.3 | 28.6 | 12.6 | 5.2 | 20.1 | 58.6 | 48.8 | 68.4 |
|  | Fever, cough, rapid breaths | 847 | 18.2 | 14.8 | 21.6 | 12.0 | 9.0 | 15.1 | 64.1 | 59.2 | 68.9 |
| **Malaria transmission season** | Off-peak | 1,001 | 23.0 | 19.6 | 26.5 | 16.1 | 12.8 | 19.4 | 56.0 | 51.3 | 60.8 |
|  | Peak | 431 | 32.5 | 26.1 | 39.0 | 22.5 | 17.1 | 27.9 | 51.3 | 44.7 | 57.8 |
| **Health card** | No (never had or lost) | 326 | 27.8 | 22.0 | 33.5 | 18.6 | 13.5 | 23.8 | 50.7 | 44.3 | 57.0 |
|  | Yes (seen or reported) | 1,106 | 25.3 | 21.9 | 28.8 | 17.8 | 14.7 | 21.0 | 55.8 | 51.3 | 60.2 |

Point estimates tabulated using sample weights pre-specified in datasets. Standard error estimation accounted for data clustering in survey designs.

**Cote d’Ivoire DHS 2011-2012**

|  |  | **N febrile under-fives taken to any care** | **Percent receiving any anti-malarial** | **95% CI** | | **Percent receiving ACT** | **95% CI** | | **Percent receiving any antibiotic** | **95% CI** | |
| --- | --- | --- | --- | --- | --- | --- | --- | --- | --- | --- | --- |
|  |  |  |  |  |  |  |  |  |  |  |  |
| **Diagnostic test use** | No | 821 | 19.2 | 15.3 | 23.2 | 2.4 | 0.7 | 4.1 | 33.0 | 28.4 | 37.6 |
|  | Yes | 131 | 42.4 | 32.1 | 52.7 | 9.2 | 3.3 | 15.1 | 45.9 | 35.9 | 56.0 |
| **Malaria endemicity** | Malaria-free | 0 | - | - | - | - | - | - | - | - | - |
|  | Unstable risk | 0 | - | - | - | - | - | - | - | - | - |
|  | Low risk (*Pf*PR_2–10_ <5%) | 0 | - | - | - | - | - | - | - | - | - |
|  | Moderate risk (*Pf*PR_2–10_ 5-40%) | 7 | - | - | - | - | - | - | - | - | - |
|  | High risk (*Pf*PR_2–10_ >40%) | 946 | 23.2 | 19.2 | 27.1 | 3.3 | 1.7 | 5.0 | 34.4 | 30.1 | 38.8 |
| **Source (public/private)** | Public | 406 | 32.8 | 25.4 | 40.2 | 4.2 | 1.7 | 6.7 | 47.5 | 40.4 | 54.6 |
|  | Private | 559 | 15.5 | 11.4 | 19.5 | 2.6 | 0.5 | 4.8 | 25.2 | 19.9 | 30.5 |
| **Source (level)** | Hospital | 165 | 40.3 | 28.8 | 51.9 | 5.4 | 0.2 | 10.5 | 54.2 | 43.1 | 65.4 |
|  | Non-hospital formal medical | 283 | 31.4 | 23.6 | 39.2 | 4.8 | 1.5 | 8.1 | 43.5 | 35.4 | 51.6 |
|  | Community health worker | 11 | 47.3 | 0.3 | 94.3 | - | - | - | 19.7 | -8.7 | 48.0 |
|  | Pharmacy | 133 | 22.9 | 13.0 | 32.8 | 5.0 | -1.5 | 11.5 | 49.2 | 36.1 | 62.4 |
|  | Other | 373 | 7.6 | 4.4 | 10.9 | 0.7 | -0.3 | 1.7 | 14.4 | 9.3 | 19.4 |
| **Child's age (months)** | 0 - 5 | 60 | 12.9 | 2.2 | 23.5 | - | - | - | 61.0 | 45.2 | 76.9 |
|  | 6 - 11 | 152 | 19.7 | 11.8 | 27.6 | 1.4 | -0.8 | 3.7 | 43.6 | 33.3 | 54.0 |
|  | 12 - 23 | 284 | 21.5 | 14.6 | 28.4 | 4.5 | 0.7 | 8.3 | 37.3 | 28.6 | 46.0 |
|  | 24 - 35 | 196 | 25.1 | 16.5 | 33.7 | 2.1 | -0.2 | 4.4 | 31.0 | 22.8 | 39.3 |
|  | 36 - 47 | 164 | 25.7 | 16.7 | 34.8 | 4.7 | -0.3 | 9.7 | 22.5 | 15.5 | 29.6 |
|  | 48 - 59 | 109 | 27.0 | 16.8 | 37.2 | 4.4 | 0.0 | 8.8 | 24.9 | 13.6 | 36.3 |
| **Child's sex** | Male | 470 | 24.9 | 19.9 | 29.9 | 3.9 | 1.3 | 6.5 | 32.1 | 25.5 | 38.7 |
|  | Female | 495 | 20.7 | 14.9 | 26.5 | 2.7 | 0.7 | 4.7 | 36.9 | 31.3 | 42.6 |
| **Maternal age (years)** | 15 -24 | 320 | 22.6 | 16.2 | 29.1 | 1.6 | -0.1 | 3.3 | 37.0 | 29.6 | 44.4 |
|  | 25 - 29 | 270 | 22.9 | 16.2 | 29.5 | 5.7 | 1.4 | 10.1 | 32.7 | 26.2 | 39.2 |
|  | 30 - 34 | 175 | 20.1 | 10.9 | 29.2 | - | - | - | 38.3 | 28.0 | 48.6 |
|  | 35 - 39 | 135 | 28.5 | 17.3 | 39.8 | 6.6 | 0.1 | 13.2 | 36.0 | 26.7 | 45.2 |
|  | 40 - 49 | 66 | 18.2 | 5.6 | 30.8 | 3.2 | -1.1 | 7.4 | 17.7 | 8.0 | 27.3 |
| **Maternal education** | No education | 582 | 19.5 | 14.9 | 24.2 | 3.7 | 1.3 | 6.0 | 34.3 | 28.2 | 40.3 |
|  | Primary | 269 | 28.6 | 20.7 | 36.5 | 2.3 | -0.1 | 4.6 | 30.7 | 23.8 | 37.6 |
|  | Secondary or higher | 114 | 25.5 | 15.6 | 35.5 | 3.8 | -0.7 | 8.2 | 45.3 | 34.3 | 56.4 |
| **Residence** | Urban | 411 | 27.3 | 21.5 | 33.0 | 2.9 | 0.8 | 5.1 | 43.3 | 36.8 | 49.7 |
|  | Rural | 554 | 19.4 | 14.2 | 24.6 | 3.5 | 1.3 | 5.8 | 28.1 | 22.4 | 33.8 |
| **Household wealth** | Poorest | 206 | 14.0 | 7.1 | 21.0 | 4.7 | -0.6 | 10.0 | 21.3 | 14.9 | 27.7 |
|  | Second | 173 | 29.0 | 19.7 | 38.4 | 3.2 | -0.1 | 6.5 | 32.5 | 22.4 | 42.6 |
|  | Middle | 223 | 17.3 | 10.1 | 24.4 | 1.5 | -0.3 | 3.3 | 35.1 | 24.4 | 45.8 |
|  | Fourth | 200 | 25.9 | 17.0 | 34.7 | 2.3 | 0.0 | 4.6 | 42.9 | 34.8 | 51.0 |
|  | Least poor | 162 | 30.9 | 22.1 | 39.6 | 5.2 | 0.3 | 10.2 | 42.7 | 32.9 | 52.6 |
| **Household members** | 1-4 members | 249 | 20.0 | 13.1 | 27.0 | 3.6 | 0.1 | 7.2 | 35.1 | 26.8 | 43.4 |
|  | 5-8 members | 415 | 24.8 | 19.0 | 30.5 | 4.0 | 1.2 | 6.8 | 32.7 | 26.5 | 39.0 |
|  | 9-12 members | 173 | 22.2 | 14.3 | 30.2 | 2.6 | -0.6 | 5.8 | 31.6 | 21.6 | 41.6 |
|  | 13 or more members | 128 | 22.1 | 10.4 | 33.7 | 1.2 | -0.8 | 3.2 | 43.7 | 30.5 | 56.8 |
| **Health care access (money)** | Big problem | 633 | 22.7 | 18.1 | 27.3 | 2.6 | 1.0 | 4.2 | 34.3 | 29.0 | 39.6 |
|  | Not a big problem | 331 | 22.9 | 15.5 | 30.4 | 4.6 | 1.1 | 8.0 | 35.1 | 27.9 | 42.2 |
| **Health care access (distance)** | Big problem | 413 | 19.8 | 13.1 | 26.5 | 1.9 | 0.4 | 3.3 | 31.5 | 25.6 | 37.5 |
|  | Not a big problem | 552 | 25.0 | 20.0 | 29.9 | 4.3 | 1.7 | 7.0 | 36.8 | 31.3 | 42.3 |
| **Symptoms** | Fever alone | 440 | 21.7 | 16.4 | 26.9 | 2.3 | 0.5 | 4.1 | 31.9 | 25.8 | 38.0 |
|  | Fever, cough | 265 | 25.0 | 17.4 | 32.7 | 3.9 | 0.5 | 7.4 | 36.3 | 28.9 | 43.7 |
|  | Fever, cough, rapid breaths | 259 | 22.3 | 15.0 | 29.7 | 4.3 | 0.6 | 7.9 | 37.1 | 28.9 | 45.4 |
| **Malaria transmission season** | Off-peak | 554 | 18.6 | 14.0 | 23.2 | 3.5 | 1.3 | 5.7 | 30.8 | 25.0 | 36.6 |
|  | Peak | 399 | 29.1 | 22.6 | 35.7 | 3.1 | 0.8 | 5.3 | 39.5 | 32.9 | 46.0 |
| **Health card** | No (never had or lost) | 61 | 13.4 | 1.8 | 24.9 | 4.8 | -4.4 | 13.9 | 19.0 | 7.8 | 30.2 |
|  | Yes (seen or reported) | 904 | 23.4 | 19.5 | 27.3 | 3.2 | 1.6 | 4.8 | 35.7 | 31.0 | 40.3 |

Point estimates tabulated using sample weights pre-specified in datasets. Standard error estimation accounted for data clustering in survey designs.

**Gabon DHS 2012**

|  |  | **N febrile under-fives taken to any care** | **Percent receiving any anti-malarial** | **95% CI** | | **Percent receiving ACT** | **95% CI** | | **Percent receiving any antibiotic** | **95% CI** | |
| --- | --- | --- | --- | --- | --- | --- | --- | --- | --- | --- | --- |
|  |  |  |  |  |  |  |  |  |  |  |  |
| **Diagnostic test use** | No | 596 | 25.0 | 17.6 | 32.3 | 10.8 | 5.9 | 15.6 | 60.9 | 53.2 | 68.6 |
|  | Yes | 124 | 32.9 | 20.5 | 45.3 | 8.6 | 3.6 | 13.5 | 51.2 | 37.6 | 64.8 |
| **Malaria endemicity** | Malaria-free | 0 | - | - | - | - | - | - | - | - | - |
|  | Unstable risk | 0 | - | - | - | - | - | - | - | - | - |
|  | Low risk (*Pf*PR_2–10_ <5%) | 0 | - | - | - | - | - | - | - | - | - |
|  | Moderate risk (*Pf*PR_2–10_ 5-40%) | 39 | 26.9 | 10.8 | 43.1 | 14.6 | 0.0 | 29.2 | 63.5 | 44.0 | 83.0 |
|  | High risk (*Pf*PR_2–10_ >40%) | 696 | 26.0 | 19.4 | 32.6 | 10.3 | 6.4 | 14.2 | 58.9 | 51.1 | 66.8 |
| **Source (public/private)** | Public | 427 | 26.6 | 20.3 | 33.0 | 10.7 | 5.9 | 15.5 | 56.3 | 47.2 | 65.3 |
|  | Private | 312 | 25.6 | 16.5 | 34.7 | 10.6 | 3.6 | 17.5 | 62.8 | 49.0 | 76.5 |
| **Source (level)** | Hospital | 310 | 30.6 | 22.5 | 38.8 | 8.8 | 3.9 | 13.7 | 57.6 | 47.4 | 67.8 |
|  | Non-hospital formal medical | 198 | 23.4 | 14.7 | 32.2 | 14.0 | 6.0 | 22.0 | 52.2 | 40.7 | 63.8 |
|  | Community health worker | 0 | - | - | - | - | - | - | - | - | - |
|  | Pharmacy | 195 | 20.7 | 13.1 | 28.3 | 6.7 | 1.9 | 11.4 | 68.3 | 51.5 | 85.2 |
|  | Other | 35 | 33.7 | -6.1 | 73.5 | 29.9 | -11.4 | 71.1 | 57.3 | 27.3 | 87.2 |
| **Child's age (months)** | 0 - 5 | 37 | 14.6 | -1.2 | 30.5 | 8.4 | -6.0 | 22.7 | 50.3 | 24.2 | 76.5 |
|  | 6 - 11 | 131 | 17.0 | 6.7 | 27.4 | 4.0 | -0.4 | 8.5 | 60.2 | 45.9 | 74.4 |
|  | 12 - 23 | 177 | 28.4 | 17.7 | 39.0 | 11.8 | 4.0 | 19.6 | 57.7 | 46.6 | 68.9 |
|  | 24 - 35 | 157 | 25.4 | 11.6 | 39.1 | 18.5 | 4.7 | 32.2 | 66.2 | 54.2 | 78.3 |
|  | 36 - 47 | 156 | 29.7 | 17.7 | 41.7 | 10.5 | 2.9 | 18.2 | 48.6 | 31.6 | 65.7 |
|  | 48 - 59 | 81 | 36.6 | 17.2 | 56.0 | 4.8 | 1.0 | 8.5 | 70.0 | 53.0 | 87.0 |
| **Child's sex** | Male | 421 | 28.1 | 21.0 | 35.3 | 11.6 | 7.0 | 16.1 | 57.6 | 49.6 | 65.6 |
|  | Female | 318 | 23.6 | 15.1 | 32.2 | 9.4 | 4.9 | 13.9 | 60.9 | 49.6 | 72.1 |
| **Maternal age (years)** | 15 -24 | 260 | 24.9 | 16.7 | 33.2 | 8.5 | 2.4 | 14.5 | 54.5 | 44.0 | 65.1 |
|  | 25 - 29 | 161 | 28.4 | 14.6 | 42.3 | 16.5 | 3.4 | 29.5 | 61.8 | 50.3 | 73.3 |
|  | 30 - 34 | 150 | 29.7 | 18.3 | 41.1 | 14.5 | 5.8 | 23.3 | 64.7 | 46.6 | 82.7 |
|  | 35 - 39 | 118 | 22.0 | 10.1 | 33.9 | 6.4 | 1.2 | 11.7 | 58.3 | 40.8 | 75.8 |
|  | 40 - 49 | 50 | 24.9 | 8.8 | 41.1 | 1.2 | -1.2 | 3.5 | 58.3 | 28.9 | 87.7 |
| **Maternal education** | No education | 45 | 13.6 | 1.1 | 26.1 | 10.7 | -1.1 | 22.5 | 58.4 | 38.5 | 78.2 |
|  | Primary | 207 | 27.4 | 18.1 | 36.7 | 9.8 | 3.2 | 16.4 | 49.2 | 37.3 | 61.2 |
|  | Secondary or higher | 487 | 26.8 | 19.9 | 33.8 | 11.0 | 5.5 | 16.4 | 63.2 | 55.0 | 71.5 |
| **Residence** | Urban | 644 | 26.2 | 19.2 | 33.3 | 10.6 | 6.4 | 14.8 | 60.4 | 51.9 | 68.9 |
|  | Rural | 95 | 26.0 | 18.5 | 33.5 | 10.7 | 6.0 | 15.5 | 49.6 | 42.1 | 57.1 |
| **Household wealth** | Poorest | 114 | 23.1 | 16.5 | 29.7 | 4.9 | 1.8 | 8.1 | 47.6 | 39.8 | 55.3 |
|  | Second | 164 | 23.4 | 13.3 | 33.5 | 6.6 | 2.1 | 11.2 | 55.6 | 42.2 | 69.0 |
|  | Middle | 148 | 22.8 | 10.1 | 35.4 | 5.1 | 0.1 | 10.0 | 70.4 | 58.9 | 81.9 |
|  | Fourth | 169 | 29.6 | 17.2 | 42.0 | 17.9 | 5.6 | 30.1 | 57.7 | 42.1 | 73.3 |
|  | Least poor | 144 | 31.4 | 19.7 | 43.1 | 17.0 | 7.5 | 26.5 | 61.8 | 41.0 | 82.6 |
| **Household members** | 1-4 members | 225 | 28.6 | 16.0 | 41.1 | 10.8 | 4.0 | 17.5 | 56.9 | 42.7 | 71.2 |
|  | 5-8 members | 334 | 29.3 | 22.3 | 36.2 | 14.4 | 8.3 | 20.5 | 60.5 | 52.5 | 68.4 |
|  | 9-12 members | 129 | 12.5 | 5.7 | 19.4 | 4.3 | 1.1 | 7.5 | 61.9 | 44.9 | 78.9 |
|  | 13 or more members | 51 | 30.3 | 12.7 | 47.8 | 1.1 | -1.1 | 3.3 | 51.3 | 31.3 | 71.4 |
| **Health care access (money)** | Big problem | 531 | 28.0 | 21.0 | 35.1 | 11.7 | 7.3 | 16.1 | 57.4 | 50.0 | 64.7 |
|  | Not a big problem | 207 | 21.5 | 12.1 | 30.9 | 7.9 | 2.7 | 13.1 | 63.4 | 45.0 | 81.8 |
| **Health care access (distance)** | Big problem | 440 | 25.4 | 18.2 | 32.6 | 10.2 | 5.4 | 15.1 | 58.9 | 49.4 | 68.4 |
|  | Not a big problem | 298 | 27.5 | 17.8 | 37.2 | 11.2 | 2.6 | 19.8 | 59.2 | 49.5 | 68.8 |
| **Symptoms** | Fever alone | 196 | 35.5 | 20.6 | 50.4 | 13.4 | 2.4 | 24.4 | 51.3 | 41.1 | 61.4 |
|  | Fever, cough | 292 | 22.3 | 13.6 | 30.9 | 7.7 | 3.1 | 12.2 | 65.9 | 56.1 | 75.7 |
|  | Fever, cough, rapid breaths | 245 | 24.0 | 13.6 | 34.3 | 12.1 | 2.9 | 21.3 | 58.3 | 47.0 | 69.6 |
| **Malaria transmission season** | Off-peak | 11 | 37.2 | -8.5 | 82.9 | 37.2 | -8.5 | 82.9 | 74.8 | 39.9 | 109.7 |
|  | Peak | 724 | 25.9 | 19.6 | 32.3 | 10.2 | 6.5 | 14.0 | 58.9 | 51.3 | 66.6 |
| **Health card** | No (never had or lost) | 48 | 34.0 | 12.9 | 55.2 | 10.4 | -6.6 | 27.5 | 38.1 | 19.5 | 56.7 |
|  | Yes (seen or reported) | 689 | 25.6 | 19.2 | 32.0 | 10.6 | 6.5 | 14.6 | 60.6 | 53.2 | 67.9 |

Point estimates tabulated using sample weights pre-specified in datasets. Standard error estimation accounted for data clustering in survey designs.

**Guinea DHS 2012**

|  |  | **N febrile under-fives taken to any care** | **Percent receiving any anti-malarial** | **95% CI** | | **Percent receiving ACT** | **95% CI** | | **Percent receiving any antibiotic** | **95% CI** | |
| --- | --- | --- | --- | --- | --- | --- | --- | --- | --- | --- | --- |
|  |  |  |  |  |  |  |  |  |  |  |  |
| **Diagnostic test use** | No | 808 | 43.0 | 38.4 | 47.7 | 1.9 | 0.6 | 3.2 | 38.3 | 33.5 | 43.1 |
|  | Yes | 118 | 53.0 | 40.9 | 65.0 | 4.1 | 0.7 | 7.5 | 54.6 | 43.7 | 65.4 |
| **Malaria endemicity** | Malaria-free | 0 | - | - | - | - | - | - | - | - | - |
|  | Unstable risk | 9 | - | - | - | - | - | - | - | - | - |
|  | Low risk (*Pf*PR_2–10_ <5%) | 0 | - | - | - | - | - | - | - | - | - |
|  | Moderate risk (*Pf*PR_2–10_ 5-40%) | 564 | 39.7 | 34.8 | 44.7 | 1.9 | 0.4 | 3.5 | 48.4 | 42.3 | 54.5 |
|  | High risk (*Pf*PR_2–10_ >40%) | 355 | 51.3 | 43.6 | 59.1 | 2.6 | 0.6 | 4.6 | 27.9 | 20.9 | 34.9 |
| **Source (public/private)** | Public | 521 | 52.8 | 46.8 | 58.8 | 3.5 | 1.5 | 5.6 | 49.3 | 43.1 | 55.4 |
|  | Private | 409 | 33.5 | 27.9 | 39.1 | 0.4 | -0.1 | 0.9 | 29.5 | 23.7 | 35.2 |
| **Source (level)** | Hospital | 93 | 40.5 | 25.6 | 55.4 | 4.1 | 0.4 | 7.8 | 56.3 | 43.3 | 69.4 |
|  | Non-hospital formal medical | 483 | 54.3 | 48.2 | 60.5 | 3.3 | 1.2 | 5.4 | 50.0 | 43.3 | 56.7 |
|  | Community health worker | 0 | - | - | - | - | - | - | - | - | - |
|  | Pharmacy | 67 | 41.5 | 23.5 | 59.5 | - | - | - | 60.1 | 44.9 | 75.2 |
|  | Other | 287 | 29.4 | 22.7 | 36.1 | 0.2 | -0.2 | 0.5 | 15.1 | 9.3 | 20.8 |
| **Child's age (months)** | 0 - 5 | 63 | 33.1 | 17.4 | 48.8 | 2.1 | -0.8 | 5.1 | 65.8 | 50.7 | 80.9 |
|  | 6 - 11 | 104 | 40.7 | 29.8 | 51.6 | 5.0 | 0.7 | 9.3 | 48.7 | 37.0 | 60.5 |
|  | 12 - 23 | 256 | 48.4 | 41.0 | 55.8 | 1.7 | -1.0 | 4.4 | 36.9 | 30.0 | 43.7 |
|  | 24 - 35 | 192 | 40.5 | 33.1 | 47.8 | 1.7 | 0.0 | 3.4 | 36.8 | 29.0 | 44.6 |
|  | 36 - 47 | 177 | 45.9 | 38.0 | 53.8 | 2.4 | -1.1 | 5.8 | 35.6 | 26.8 | 44.4 |
|  | 48 - 59 | 139 | 47.9 | 37.3 | 58.5 | 1.3 | -0.5 | 3.1 | 41.5 | 30.8 | 52.3 |
| **Child's sex** | Male | 478 | 45.2 | 38.9 | 51.5 | 2.0 | 0.8 | 3.2 | 40.6 | 34.9 | 46.3 |
|  | Female | 452 | 43.4 | 37.7 | 49.1 | 2.3 | 0.1 | 4.6 | 40.5 | 34.5 | 46.5 |
| **Maternal age (years)** | 15 -24 | 301 | 40.5 | 33.8 | 47.2 | 1.7 | 0.2 | 3.2 | 44.0 | 37.2 | 50.8 |
|  | 25 - 29 | 235 | 54.9 | 46.7 | 63.1 | 2.5 | -0.3 | 5.2 | 39.5 | 31.1 | 48.0 |
|  | 30 - 34 | 166 | 47.5 | 38.2 | 56.8 | 2.2 | 0.0 | 4.4 | 43.7 | 34.1 | 53.4 |
|  | 35 - 39 | 151 | 38.6 | 29.2 | 48.1 | 3.5 | -1.0 | 8.1 | 28.9 | 19.7 | 38.0 |
|  | 40 - 49 | 78 | 31.3 | 18.5 | 44.2 | 0.3 | -0.3 | 0.9 | 46.6 | 31.8 | 61.3 |
| **Maternal education** | No education | 675 | 45.3 | 40.3 | 50.3 | 2.6 | 1.0 | 4.1 | 36.6 | 31.5 | 41.7 |
|  | Primary | 127 | 47.3 | 36.0 | 58.6 | - | - | - | 46.7 | 37.8 | 55.6 |
|  | Secondary or higher | 129 | 36.2 | 27.0 | 45.4 | 2.2 | -0.2 | 4.7 | 55.3 | 42.2 | 68.4 |
| **Residence** | Urban | 239 | 41.9 | 34.9 | 49.0 | 5.0 | 1.6 | 8.5 | 51.9 | 41.2 | 62.5 |
|  | Rural | 692 | 45.2 | 39.8 | 50.5 | 1.2 | 0.2 | 2.2 | 36.7 | 31.5 | 41.8 |
| **Household wealth** | Poorest | 183 | 39.7 | 32.1 | 47.3 | 3.0 | -0.4 | 6.3 | 32.2 | 24.5 | 39.9 |
|  | Second | 200 | 45.5 | 35.3 | 55.7 | - | - | - | 33.7 | 23.8 | 43.7 |
|  | Middle | 212 | 48.3 | 39.6 | 56.9 | 1.1 | -0.2 | 2.3 | 33.6 | 25.3 | 42.0 |
|  | Fourth | 193 | 49.9 | 39.9 | 59.8 | 5.0 | 0.3 | 9.7 | 50.3 | 42.5 | 58.1 |
|  | Least poor | 143 | 35.3 | 25.6 | 45.1 | 2.0 | -0.1 | 4.1 | 58.1 | 43.4 | 72.8 |
| **Household members** | 1-4 members | 142 | 39.8 | 30.9 | 48.7 | 2.3 | -0.4 | 4.9 | 35.0 | 24.4 | 45.6 |
|  | 5-8 members | 433 | 46.3 | 40.1 | 52.5 | 1.1 | 0.2 | 2.0 | 43.2 | 36.9 | 49.5 |
|  | 9-12 members | 224 | 44.4 | 36.0 | 52.7 | 4.5 | 0.3 | 8.7 | 35.8 | 28.4 | 43.2 |
|  | 13 or more members | 131 | 42.7 | 30.7 | 54.7 | 1.6 | -0.4 | 3.6 | 46.1 | 33.9 | 58.3 |
| **Health care access (money)** | Big problem | - | - | - | - | - | - | - | - | - | - |
|  | Not a big problem | - | - | - | - | - | - | - | - | - | - |
| **Health care access (distance)** | Big problem | - | - | - | - | - | - | - | - | - | - |
|  | Not a big problem | - | - | - | - | - | - | - | - | - | - |
| **Symptoms** | Fever alone | 525 | 49.4 | 43.6 | 55.2 | 1.9 | 0.3 | 3.4 | 33.4 | 27.4 | 39.4 |
|  | Fever, cough | 141 | 40.9 | 31.7 | 50.1 | 4.1 | -0.2 | 8.3 | 44.2 | 35.2 | 53.2 |
|  | Fever, cough, rapid breaths | 264 | 36.1 | 29.1 | 43.1 | 1.7 | 0.0 | 3.4 | 52.9 | 45.9 | 59.9 |
| **Malaria transmission season** | Off-peak | 11 | 55.0 | 30.3 | 79.7 | - | - | - | 45.0 | 20.3 | 69.7 |
|  | Peak | 919 | 44.2 | 39.8 | 48.6 | 2.2 | 1.0 | 3.4 | 40.5 | 35.8 | 45.2 |
| **Health card** | No (never had or lost) | 168 | 35.9 | 28.3 | 43.5 | 0.3 | -0.3 | 0.8 | 29.1 | 21.8 | 37.6 |
|  | Yes (seen or reported) | 762 | 46.1 | 41.3 | 51.0 | 2.6 | 1.1 | 4.0 | 43.1 | 38.0 | 48.3 |

Point estimates tabulated using sample weights pre-specified in datasets. Standard error estimation accounted for data clustering in survey designs.

**Malawi DHS 2010**

|  |  | **N febrile under-fives taken to any care** | **Percent receiving any anti-malarial** | **95% CI** | | **Percent receiving ACT** | **95% CI** | | **Percent receiving any antibiotic** | **95% CI** | |
| --- | --- | --- | --- | --- | --- | --- | --- | --- | --- | --- | --- |
|  |  |  |  |  |  |  |  |  |  |  |  |
| **Diagnostic test use** | No | 3,363 | 52.6 | 50.1 | 55.1 | 45.6 | 43.0 | 48.1 | 28.5 | 26.3 | 30.7 |
|  | Yes | 928 | 64.2 | 59.1 | 69.3 | 50.6 | 45.3 | 55.9 | 33.8 | 29.2 | 38.5 |
| **Malaria endemicity** | Malaria-free | 0 | - | - | - | - | - | - | - | - | - |
|  | Unstable risk | 0 | - | - | - | - | - | - | - | - | - |
|  | Low risk (*Pf*PR_2–10_ <5%) | 0 | - | - | - | - | - | - | - | - | - |
|  | Moderate risk (*Pf*PR_2–10_ 5-40%) | 2,361 | 53.2 | 49.5 | 56.9 | 43.5 | 39.7 | 47.3 | 31.4 | 28.4 | 34.5 |
|  | High risk (*Pf*PR_2–10_ >40%) | 1,870 | 56.9 | 53.8 | 60.1 | 50.1 | 47.0 | 53.2 | 27.7 | 24.9 | 30.5 |
| **Source (public/private)** | Public | 2,978 | 61.3 | 58.6 | 63.9 | 55.1 | 52.4 | 57.9 | 27.6 | 25.2 | 30.0 |
|  | Private | 1,358 | 41.2 | 36.9 | 45.4 | 27.8 | 23.8 | 31.8 | 34.0 | 30.2 | 37.8 |
| **Source (level)** | Hospital | 1,265 | 62.4 | 58.3 | 66.6 | 49.4 | 44.6 | 54.1 | 34.6 | 30.7 | 38.6 |
|  | Non-hospital formal medical | 2,535 | 60.2 | 57.3 | 63.1 | 53.1 | 50.1 | 56.1 | 28.3 | 25.6 | 30.9 |
|  | Community health worker | 0 | - | - | - | - | - | - | - | - | - |
|  | Pharmacy | 2 | - | - | - | - | - | - | - | - | - |
|  | Other | 535 | 12.3 | 8.7 | 16.0 | 8.5 | 5.3 | 11.8 | 24.0 | 19.3 | 28.8 |
| **Child's age (months)** | 0 - 5 | 255 | 27.1 | 20.3 | 33.9 | 19.6 | 13.4 | 25.8 | 44.1 | 36.2 | 51.9 |
|  | 6 - 11 | 642 | 46.0 | 40.3 | 51.7 | 39.3 | 33.7 | 45.0 | 34.8 | 30.0 | 39.6 |
|  | 12 - 23 | 1,104 | 58.6 | 54.9 | 62.3 | 49.5 | 45.7 | 53.3 | 27.3 | 23.7 | 30.8 |
|  | 24 - 35 | 965 | 60.6 | 56.2 | 65.0 | 51.6 | 46.9 | 56.3 | 29.0 | 25.2 | 32.7 |
|  | 36 - 47 | 742 | 57.9 | 53.1 | 62.7 | 49.2 | 44.2 | 54.2 | 27.0 | 23.0 | 30.9 |
|  | 48 - 59 | 629 | 56.8 | 50.8 | 62.9 | 48.8 | 42.9 | 54.6 | 26.7 | 22.7 | 30.8 |
| **Child's sex** | Male | 2,207 | 55.3 | 52.4 | 58.2 | 45.8 | 42.8 | 48.7 | 28.8 | 26.2 | 31.5 |
|  | Female | 2,130 | 54.6 | 51.3 | 58.0 | 47.4 | 44.0 | 50.8 | 30.4 | 27.7 | 33.2 |
| **Maternal age (years)** | 15 -24 | 1,517 | 52.5 | 49.1 | 55.9 | 44.6 | 41.2 | 47.9 | 30.0 | 26.7 | 33.3 |
|  | 25 - 29 | 1,297 | 56.3 | 52.4 | 60.2 | 47.6 | 43.6 | 51.5 | 32.1 | 28.2 | 36.0 |
|  | 30 - 34 | 738 | 57.1 | 52.6 | 61.6 | 47.2 | 42.4 | 52.1 | 28.2 | 23.9 | 32.4 |
|  | 35 - 39 | 499 | 54.7 | 49.2 | 60.3 | 45.3 | 39.8 | 50.8 | 28.4 | 23.7 | 33.2 |
|  | 40 - 49 | 287 | 56.9 | 49.5 | 64.3 | 52.7 | 45.2 | 60.3 | 22.5 | 16.3 | 28.6 |
| **Maternal education** | No education | 622 | 53.1 | 47.9 | 58.3 | 45.2 | 39.8 | 50.7 | 24.8 | 20.6 | 29.0 |
|  | Primary | 3,092 | 55.1 | 52.4 | 57.9 | 47.7 | 44.9 | 50.4 | 28.6 | 26.2 | 30.9 |
|  | Secondary or higher | 623 | 56.0 | 50.5 | 61.4 | 42.4 | 36.9 | 47.8 | 39.7 | 34.5 | 44.9 |
| **Residence** | Urban | 551 | 53.9 | 44.7 | 63.1 | 43.7 | 33.5 | 53.8 | 42.2 | 36.0 | 48.5 |
|  | Rural | 3,786 | 55.1 | 52.7 | 57.6 | 47.0 | 44.5 | 49.4 | 27.8 | 25.7 | 29.9 |
| **Household wealth** | Poorest | 912 | 54.2 | 49.8 | 58.5 | 47.2 | 42.9 | 51.6 | 24.9 | 21.1 | 28.7 |
|  | Second | 932 | 56.0 | 51.8 | 60.2 | 49.7 | 45.4 | 53.9 | 23.8 | 20.4 | 27.2 |
|  | Middle | 1,063 | 53.7 | 49.3 | 58.0 | 46.9 | 42.3 | 51.5 | 31.4 | 27.4 | 35.3 |
|  | Fourth | 794 | 55.0 | 50.1 | 59.9 | 44.7 | 39.7 | 49.7 | 34.0 | 29.2 | 38.9 |
|  | Least poor | 637 | 56.8 | 50.3 | 63.2 | 42.8 | 37.4 | 48.1 | 36.5 | 29.9 | 43.1 |
| **Household members** | 1-4 members | 1,428 | 55.0 | 51.5 | 58.6 | 46.6 | 43.1 | 50.1 | 30.0 | 26.3 | 33.7 |
|  | 5-8 members | 2,445 | 54.7 | 51.6 | 57.8 | 46.4 | 43.4 | 49.5 | 29.3 | 26.6 | 32.0 |
|  | 9-12 members | 416 | 55.0 | 49.7 | 60.3 | 46.0 | 40.4 | 51.6 | 29.7 | 23.8 | 35.5 |
|  | 13 or more members | 48 | 65.3 | 46.5 | 84.0 | 55.2 | 37.4 | 73.0 | 34.0 | 20.5 | 47.5 |
| **Health care access (money)** | Big problem | 2,402 | 54.4 | 51.1 | 57.6 | 47.2 | 43.9 | 50.5 | 28.9 | 26.4 | 31.5 |
|  | Not a big problem | 1,932 | 55.7 | 52.4 | 59.1 | 45.7 | 42.4 | 49.1 | 30.5 | 27.4 | 33.5 |
| **Health care access (distance)** | Big problem | 2,584 | 54.9 | 51.8 | 57.9 | 47.2 | 44.1 | 50.3 | 28.5 | 26.1 | 30.8 |
|  | Not a big problem | 1,751 | 55.1 | 51.8 | 58.5 | 45.6 | 42.2 | 49.0 | 31.3 | 27.9 | 34.8 |
| **Symptoms** | Fever alone | 1,980 | 60.1 | 57.1 | 63.2 | 51.7 | 48.4 | 54.9 | 18.2 | 16.0 | 20.4 |
|  | Fever, cough | 920 | 52.9 | 48.4 | 57.5 | 46.5 | 42.1 | 51.0 | 40.0 | 35.4 | 44.6 |
|  | Fever, cough, rapid breaths | 1,436 | 49.1 | 45.4 | 52.8 | 39.5 | 35.6 | 43.3 | 38.7 | 35.0 | 42.5 |
| **Malaria transmission season** | Off-peak | 4,207 | 54.7 | 52.2 | 57.3 | 46.3 | 43.8 | 48.9 | 29.8 | 27.7 | 32.0 |
|  | Peak | 23 | 69.4 | 59.0 | 79.8 | 58.1 | 52.1 | 64.2 | 21.8 | 3.6 | 40.0 |
| **Health card** | No (never had or lost) | 462 | 54.2 | 47.8 | 60.6 | 43.8 | 37.7 | 49.9 | 24.5 | 19.4 | 29.5 |
|  | Yes (seen or reported) | 3,871 | 55.0 | 52.5 | 57.6 | 46.8 | 44.2 | 49.4 | 30.3 | 28.1 | 32.4 |

Point estimates tabulated using sample weights pre-specified in datasets. Standard error estimation accounted for data clustering in survey designs.

**Mozambique DHS 2011**

|  |  | **N febrile under-fives taken to any care** | **Percent receiving any anti-malarial** | **95% CI** | | **Percent receiving ACT** | **95% CI** | | **Percent receiving any antibiotic** | **95% CI** | |
| --- | --- | --- | --- | --- | --- | --- | --- | --- | --- | --- | --- |
|  |  |  |  |  |  |  |  |  |  |  |  |
| **Diagnostic test use** | No | 502 | 31.3 | 25.9 | 36.8 | 14.3 | 10.2 | 18.3 | 11.4 | 8.0 | 14.8 |
|  | Yes | 386 | 55.5 | 48.9 | 62.0 | 39.7 | 32.5 | 46.9 | 12.8 | 9.0 | 16.6 |
| **Malaria endemicity** | Malaria-free | 0 | - | - | - | - | - | - | - | - | - |
|  | Unstable risk | 0 | - | - | - | - | - | - | - | - | - |
|  | Low risk (*Pf*PR_2–10_ <5%) | 0 | - | - | - | - | - | - | - | - | - |
|  | Moderate risk (*Pf*PR_2–10_ 5-40%) | 416 | 34.4 | 28.6 | 40.2 | 19.5 | 15.1 | 23.9 | 17.6 | 13.8 | 21.4 |
|  | High risk (*Pf*PR_2–10_ >40%) | 472 | 48.3 | 40.9 | 55.7 | 30.4 | 22.5 | 38.2 | 7.1 | 4.0 | 10.1 |
| **Source (public/private)** | Public | 782 | 43.8 | 38.8 | 48.8 | 27.6 | 22.5 | 32.6 | 13.4 | 10.7 | 16.1 |
|  | Private | 106 | 26.8 | 16.1 | 37.6 | 8.6 | 3.1 | 14.1 | 2.0 | -0.4 | 4.4 |
| **Source (level)** | Public facility; private hospital | 776 | 43.0 | 38.0 | 48.0 | 26.7 | 21.6 | 31.7 | 13.5 | 10.8 | 16.2 |
|  | Mobile outreach; private doctor | 11 | 85.4 | 65.6 | 105.3 | 80.5 | 58.3 | 102.7 | 4.9 | -4.9 | 14.8 |
|  | Community health worker | 44 | 31.3 | 10.1 | 52.4 | 7.0 | -0.2 | 14.1 | 1.5 | -1.5 | 4.4 |
|  | Pharmacy | 4 | 47.3 | -1.8 | 96.5 | 37.5 | -14.2 | 89.4 | - | - | - |
|  | Other | 52 | 23.7 | 11.1 | 36.4 | 8.4 | 1.3 | 15.5 | 1.7 | -1.7 | 5.0 |
| **Child's age (months)** | 0 - 5 | 62 | 18.9 | 6.1 | 31.8 | 16.5 | 4.0 | 29.0 | 21.8 | 7.0 | 36.7 |
|  | 6 - 11 | 152 | 30.3 | 20.8 | 39.7 | 16.1 | 9.5 | 22.7 | 12.6 | 7.4 | 17.9 |
|  | 12 - 23 | 241 | 38.2 | 31.3 | 45.2 | 21.3 | 15.3 | 27.3 | 11.8 | 7.3 | 16.2 |
|  | 24 - 35 | 174 | 45.5 | 36.5 | 54.5 | 28.6 | 21.1 | 36.0 | 9.1 | 4.9 | 13.3 |
|  | 36 - 47 | 141 | 61.7 | 50.8 | 72.6 | 35.5 | 24.2 | 46.7 | 13.3 | 6.9 | 19.8 |
|  | 48 - 59 | 119 | 46.7 | 31.5 | 62.0 | 32.9 | 16.5 | 49.4 | 9.3 | 3.5 | 15.1 |
| **Child's sex** | Male | 445 | 43.7 | 37.7 | 49.7 | 25.9 | 19.9 | 31.9 | 11.0 | 7.6 | 14.5 |
|  | Female | 443 | 39.9 | 34.2 | 45.6 | 24.7 | 19.5 | 29.9 | 13.0 | 9.5 | 16.5 |
| **Maternal age (years)** | 15 -24 | 322 | 36.3 | 30.5 | 42.1 | 20.5 | 15.7 | 25.4 | 12.8 | 9.0 | 16.6 |
|  | 25 - 29 | 205 | 40.0 | 32.2 | 47.8 | 26.5 | 19.5 | 33.4 | 16.4 | 10.3 | 22.4 |
|  | 30 - 34 | 179 | 48.0 | 34.2 | 61.7 | 30.0 | 14.0 | 46.0 | 5.5 | 2.2 | 8.8 |
|  | 35 - 39 | 107 | 42.7 | 29.7 | 55.6 | 25.3 | 14.7 | 36.0 | 13.4 | 4.1 | 22.8 |
|  | 40 - 49 | 76 | 54.3 | 39.6 | 68.9 | 31.0 | 18.2 | 43.8 | 10.2 | 3.1 | 17.4 |
| **Maternal education** | No education | 268 | 46.5 | 37.2 | 55.9 | 28.7 | 19.5 | 38.0 | 10.6 | 6.3 | 14.8 |
|  | Primary | 512 | 42.8 | 37.2 | 48.4 | 25.7 | 20.9 | 30.5 | 10.1 | 6.8 | 13.4 |
|  | Secondary or higher | 108 | 25.1 | 16.2 | 34.1 | 14.7 | 7.2 | 22.2 | 24.6 | 16.1 | 33.1 |
| **Residence** | Urban | 279 | 31.3 | 24.9 | 37.7 | 17.7 | 12.5 | 22.9 | 13.6 | 9.5 | 17.8 |
|  | Rural | 609 | 46.6 | 40.6 | 52.6 | 28.8 | 22.6 | 35.0 | 11.3 | 8.2 | 14.3 |
| **Household wealth** | Poorest | 199 | 56.9 | 45.8 | 68.1 | 33.9 | 21.9 | 45.9 | 7.7 | 2.7 | 12.6 |
|  | Second | 163 | 41.9 | 31.4 | 52.4 | 27.8 | 18.7 | 36.8 | 9.8 | 4.2 | 15.5 |
|  | Middle | 187 | 50.5 | 41.6 | 59.5 | 31.0 | 22.2 | 39.8 | 9.5 | 5.1 | 13.8 |
|  | Fourth | 200 | 31.7 | 24.8 | 38.6 | 19.2 | 13.0 | 25.5 | 16.4 | 10.0 | 22.9 |
|  | Least poor | 140 | 23.0 | 13.4 | 32.5 | 11.2 | 5.4 | 17.0 | 17.8 | 11.4 | 24.2 |
| **Household members** | 1-4 members | 269 | 37.0 | 30.7 | 43.4 | 19.1 | 14.3 | 24.0 | 9.5 | 5.9 | 13.1 |
|  | 5-8 members | 508 | 45.3 | 38.5 | 52.0 | 28.0 | 20.8 | 35.2 | 9.3 | 6.2 | 12.4 |
|  | 9-12 members | 87 | 41.4 | 27.0 | 55.8 | 30.6 | 17.1 | 44.1 | 30.8 | 20.9 | 40.6 |
|  | 13 or more members | 24 | 23.7 | 5.6 | 41.7 | 17.9 | 1.3 | 34.5 | 29.1 | 10.5 | 47.7 |
| **Health care access (money)** | Big problem | 385 | 44.6 | 37.0 | 52.2 | 20.9 | 13.8 | 28.1 | 9.7 | 5.7 | 13.8 |
|  | Not a big problem | 503 | 39.7 | 34.4 | 45.0 | 28.6 | 23.4 | 33.8 | 13.8 | 10.8 | 16.7 |
| **Health care access (distance)** | Big problem | 475 | 46.8 | 40.5 | 53.1 | 27.0 | 20.9 | 33.1 | 10.4 | 6.8 | 14.0 |
|  | Not a big problem | 413 | 36.1 | 30.0 | 42.1 | 23.4 | 17.7 | 29.0 | 13.9 | 10.5 | 17.3 |
| **Symptoms** | Fever alone | 553 | 44.5 | 39.5 | 49.5 | 25.7 | 21.0 | 30.4 | 9.9 | 7.1 | 12.7 |
|  | Fever, cough | 171 | 37.5 | 27.9 | 47.0 | 23.2 | 16.0 | 30.5 | 15.5 | 10.2 | 20.9 |
|  | Fever, cough, rapid breaths | 164 | 37.4 | 25.3 | 49.5 | 26.1 | 13.0 | 39.2 | 15.5 | 8.7 | 22.3 |
| **Malaria transmission season** | Off-peak | 764 | 40.9 | 35.9 | 45.9 | 27.2 | 22.0 | 32.3 | 13.3 | 10.7 | 15.9 |
|  | Peak | 124 | 47.5 | 33.8 | 61.3 | 13.8 | 6.9 | 20.6 | 3.9 | -0.5 | 8.3 |
| **Health card** | No (never had or lost) | 74 | 50.8 | 35.2 | 66.2 | 30.2 | 15.2 | 45.2 | 4.1 | -0.5 | 8.8 |
|  | Yes (seen or reported) | 814 | 41.0 | 36.1 | 46.0 | 24.8 | 19.8 | 29.9 | 12.7 | 10.1 | 15.4 |

Point estimates tabulated using sample weights pre-specified in datasets. Standard error estimation accounted for data clustering in survey designs.

**Rwanda DHS 2010-2011**

|  |  | **N febrile under-fives taken to any care** | **Percent receiving any anti-malarial** | **95% CI** | | **Percent receiving ACT** | **95% CI** | | **Percent receiving any antibiotic** | **95% CI** | |
| --- | --- | --- | --- | --- | --- | --- | --- | --- | --- | --- | --- |
|  |  |  |  |  |  |  |  |  |  |  |  |
| **Diagnostic test use** | No | 413 | 20.6 | 16.5 | 24.7 | 19.6 | 15.6 | 23.6 | 39.5 | 34.4 | 44.5 |
|  | Yes | 240 | 20.4 | 15.5 | 25.3 | 19.9 | 15.1 | 24.7 | 65.4 | 59.2 | 71.6 |
| **Malaria endemicity** | Malaria-free | 118 | 2.5 | -0.3 | 5.3 | 2.5 | -0.3 | 5.3 | 65.3 | 56.3 | 74.4 |
|  | Unstable risk | 0 | - | - | - | - | - | - | - | - | - |
|  | Low risk (*Pf*PR_2–10_ <5%) | 345 | 16.3 | 12.0 | 20.7 | 15.8 | 11.5 | 20.0 | 53.5 | 47.8 | 59.2 |
|  | Moderate risk (*Pf*PR_2–10_ 5-40%) | 194 | 38.6 | 31.8 | 45.5 | 36.9 | 30.2 | 43.6 | 31.4 | 24.0 | 38.7 |
|  | High risk (*Pf*PR_2–10_ >40%) | 0 | - | - | - | - | - | - | - | - | - |
| **Source (public/private)** | Public | 522 | 24.4 | 20.6 | 28.2 | 23.5 | 19.8 | 27.3 | 51.2 | 46.6 | 55.7 |
|  | Private | 134 | 5.0 | 1.1 | 9.0 | 4.4 | 0.6 | 8.2 | 41.1 | 31.3 | 51.0 |
| **Source (level)** | Hospital | 19 | 6.1 | -5.9 | 18.1 | 6.1 | -5.9 | 18.1 | 79.3 | 59.3 | 99.3 |
|  | Non-hospital formal medical | 327 | 13.8 | 10.0 | 17.6 | 12.5 | 8.7 | 16.2 | 70.5 | 65.0 | 75.9 |
|  | Community health worker | 188 | 43.2 | 35.5 | 50.8 | 43.2 | 35.5 | 50.8 | 17.5 | 11.8 | 23.3 |
|  | Pharmacy | 58 | 11.6 | 2.8 | 20.4 | 10.1 | 1.7 | 18.5 | 69.4 | 57.9 | 81.0 |
|  | Other | 65 | - | - | - | - | - | - | 5.7 | -0.7 | 12.1 |
| **Child's age (months)** | 0 - 5 | 32 | 5.6 | -2.3 | 13.4 | 2.1 | -2.1 | 6.4 | 45.4 | 26.3 | 64.4 |
|  | 6 - 11 | 109 | 13.1 | 5.6 | 20.5 | 12.0 | 4.7 | 19.3 | 59.2 | 49.7 | 68.7 |
|  | 12 - 23 | 184 | 20.9 | 15.2 | 26.7 | 20.3 | 14.6 | 26.1 | 53.1 | 45.7 | 60.5 |
|  | 24 - 35 | 147 | 21.2 | 14.4 | 28.1 | 20.5 | 13.9 | 27.1 | 46.2 | 38.1 | 54.2 |
|  | 36 - 47 | 102 | 23.6 | 14.9 | 32.3 | 23.6 | 14.9 | 32.3 | 38.5 | 28.3 | 48.6 |
|  | 48 - 59 | 83 | 29.3 | 19.2 | 39.4 | 28.3 | 18.3 | 38.3 | 46.6 | 35.8 | 57.5 |
| **Child's sex** | Male | 354 | 20.0 | 15.7 | 24.4 | 19.5 | 15.3 | 23.7 | 50.9 | 45.5 | 56.3 |
|  | Female | 302 | 20.9 | 16.5 | 25.3 | 19.8 | 15.3 | 24.2 | 47.0 | 41.1 | 53.0 |
| **Maternal age (years)** | 15 -24 | 163 | 15.3 | 9.3 | 21.2 | 14.6 | 9.0 | 20.2 | 50.2 | 42.0 | 58.4 |
|  | 25 - 29 | 205 | 23.4 | 17.1 | 29.7 | 22.8 | 16.6 | 29.0 | 48.7 | 40.9 | 56.4 |
|  | 30 - 34 | 144 | 16.3 | 10.1 | 22.5 | 14.9 | 8.9 | 21.0 | 55.9 | 46.8 | 65.0 |
|  | 35 - 39 | 87 | 28.0 | 17.6 | 38.3 | 26.7 | 16.8 | 36.7 | 38.8 | 27.3 | 50.2 |
|  | 40 - 49 | 58 | 23.3 | 12.6 | 33.9 | 23.3 | 12.6 | 33.9 | 46.5 | 33.1 | 59.9 |
| **Maternal education** | No education | 90 | 25.8 | 16.9 | 34.6 | 25.8 | 16.9 | 34.6 | 43.8 | 32.4 | 55.3 |
|  | Primary | 492 | 20.8 | 16.9 | 24.6 | 19.7 | 15.9 | 23.5 | 45.5 | 40.6 | 50.3 |
|  | Secondary or higher | 75 | 11.8 | 4.0 | 19.5 | 11.8 | 4.0 | 19.5 | 79.4 | 69.5 | 89.2 |
| **Residence** | Urban | 109 | 10.4 | 4.7 | 16.0 | 10.4 | 4.7 | 16.0 | 67.0 | 58.1 | 76.0 |
|  | Rural | 548 | 22.4 | 18.9 | 25.9 | 21.5 | 18.0 | 24.9 | 45.5 | 41.0 | 50.1 |
| **Household wealth** | Poorest | 143 | 28.0 | 19.5 | 36.6 | 28.0 | 19.5 | 36.6 | 34.7 | 26.3 | 43.1 |
|  | Second | 125 | 19.5 | 12.4 | 26.6 | 17.1 | 10.5 | 23.7 | 47.4 | 37.6 | 57.2 |
|  | Middle | 126 | 22.4 | 14.2 | 30.6 | 21.5 | 13.5 | 29.6 | 46.5 | 37.2 | 55.7 |
|  | Fourth | 114 | 15.1 | 8.5 | 21.7 | 15.1 | 8.5 | 21.7 | 42.7 | 33.5 | 51.9 |
|  | Least poor | 148 | 16.2 | 9.8 | 22.6 | 15.4 | 9.2 | 21.7 | 71.7 | 64.3 | 79.1 |
| **Household members** | 1-4 members | 269 | 21.0 | 16.1 | 25.9 | 20.3 | 15.4 | 25.2 | 50.0 | 43.4 | 56.7 |
|  | 5-8 members | 340 | 21.2 | 16.8 | 25.6 | 20.3 | 16.0 | 24.5 | 46.1 | 40.3 | 52.0 |
|  | 9-12 members | 45 | 11.5 | -0.4 | 23.5 | 11.5 | -0.4 | 23.5 | 63.5 | 48.6 | 78.4 |
|  | 13 or more members | 2 | - | - | - | - | - | - | - | - | - |
| **Health care access (money)** | Big problem | 363 | 20.5 | 15.9 | 25.0 | 19.6 | 15.2 | 24.1 | 46.9 | 41.3 | 52.5 |
|  | Not a big problem | 293 | 20.3 | 15.5 | 25.1 | 19.6 | 14.8 | 24.3 | 51.8 | 45.6 | 58.1 |
| **Health care access (distance)** | Big problem | 180 | 23.1 | 16.4 | 29.7 | 22.0 | 15.5 | 28.5 | 46.9 | 39.2 | 54.6 |
|  | Not a big problem | 477 | 19.4 | 15.8 | 23.1 | 18.7 | 15.1 | 22.3 | 49.9 | 44.9 | 55.0 |
| **Symptoms** | Fever alone | 220 | 30.4 | 24.6 | 36.2 | 30.0 | 24.2 | 35.7 | 32.9 | 26.3 | 39.4 |
|  | Fever, cough | 175 | 17.0 | 11.4 | 22.6 | 16.4 | 10.9 | 21.9 | 51.0 | 43.0 | 59.0 |
|  | Fever, cough, rapid breaths | 262 | 14.3 | 9.7 | 18.8 | 13.1 | 8.6 | 17.6 | 61.5 | 54.8 | 68.2 |
| **Malaria transmission season** | Off-peak | 406 | 13.7 | 10.0 | 17.4 | 12.9 | 9.5 | 16.4 | 51.8 | 46.4 | 57.2 |
|  | Peak | 250 | 31.3 | 25.3 | 37.3 | 30.4 | 24.4 | 36.5 | 44.7 | 37.8 | 51.7 |
| **Health card** | No (never had or lost) | 93 | 28.7 | 18.0 | 39.4 | 28.7 | 18.0 | 39.3 | 39.5 | 28.8 | 50.2 |
|  | Yes (seen or reported) | 563 | 19.0 | 15.8 | 22.3 | 18.1 | 14.9 | 21.3 | 50.7 | 46.2 | 55.2 |

Point estimates tabulated using sample weights pre-specified in datasets. Standard error estimation accounted for data clustering in survey designs.

**Senegal DHS 2010-2011**

|  |  | **N febrile under-fives taken to any care** | **Percent receiving any anti-malarial** | **95% CI** | | **Percent receiving ACT** | **95% CI** | | **Percent receiving any antibiotic** | **95% CI** | |
| --- | --- | --- | --- | --- | --- | --- | --- | --- | --- | --- | --- |
|  |  |  |  |  |  |  |  |  |  |  |  |
| **Diagnostic test use** | No | 1,078 | 13.0 | 9.4 | 16.7 | 4.9 | 2.3 | 7.6 | 41.5 | 36.2 | 46.8 |
|  | Yes | 190 | 20.4 | 11.2 | 29.6 | 8.8 | 3.6 | 13.9 | 51.9 | 40.1 | 63.6 |
| **Malaria endemicity** | Malaria-free | 0 | - | - | - | - | - | - | - | - | - |
|  | Unstable risk | 0 | - | - | - | - | - | - | - | - | - |
|  | Low risk (*Pf*PR_2–10_ <5%) | 814 | 15.0 | 9.6 | 20.5 | 6.5 | 2.7 | 10.2 | 44.0 | 36.2 | 51.8 |
|  | Moderate risk (*Pf*PR_2–10_ 5-40%) | 442 | 12.3 | 9.4 | 15.2 | 3.8 | 2.4 | 5.3 | 41.3 | 35.5 | 47.1 |
|  | High risk (*Pf*PR_2–10_ >40%) | 11 | 22.2 | 8.9 | 35.4 | 7.4 | -6.3 | 21.1 | 26.9 | 6.0 | 47.8 |
| **Source (public/private)** | Public | 916 | 15.9 | 12.1 | 19.7 | 6.2 | 3.3 | 9.2 | 49.5 | 43.7 | 55.4 |
|  | Private | 359 | 9.5 | 3.0 | 16.0 | 3.7 | 0.8 | 6.7 | 26.1 | 18.9 | 33.3 |
| **Source (level)** | Hospital | 203 | 18.8 | 8.2 | 29.3 | 8.4 | -0.2 | 17.0 | 46.6 | 35.2 | 57.9 |
|  | Non-hospital formal medical | 737 | 14.5 | 11.4 | 17.6 | 5.4 | 3.1 | 7.6 | 50.9 | 44.4 | 57.4 |
|  | Community health worker | 40 | 5.9 | -0.2 | 12.0 | 1.4 | -1.4 | 4.3 | 24.0 | 11.4 | 36.6 |
|  | Pharmacy | 134 | 18.6 | 4.0 | 33.1 | 8.8 | 1.7 | 16.0 | 24.9 | 16.4 | 33.3 |
|  | Other | 161 | 4.8 | 1.4 | 8.1 | 1.0 | -0.1 | 2.1 | 21.8 | 10.8 | 32.8 |
| **Child's age (months)** | 0 - 5 | 127 | 10.8 | 2.6 | 19.0 | 3.5 | -1.1 | 8.0 | 42.4 | 31.7 | 53.1 |
|  | 6 - 11 | 195 | 9.7 | 4.5 | 14.9 | 2.9 | -0.3 | 6.1 | 31.4 | 21.0 | 41.8 |
|  | 12 - 23 | 293 | 12.0 | 6.8 | 17.1 | 3.8 | 0.8 | 6.7 | 46.6 | 38.4 | 54.8 |
|  | 24 - 35 | 267 | 17.5 | 11.3 | 23.6 | 4.9 | 1.9 | 7.9 | 48.6 | 38.7 | 58.6 |
|  | 36 - 47 | 253 | 18.0 | 9.2 | 26.9 | 11.1 | 2.2 | 20.0 | 40.7 | 32.3 | 49.2 |
|  | 48 - 59 | 140 | 14.3 | 5.9 | 22.7 | 5.9 | 0.0 | 11.9 | 45.0 | 35.4 | 54.7 |
| **Child's sex** | Male | 693 | 16.1 | 11.5 | 20.6 | 5.1 | 1.6 | 8.7 | 42.3 | 36.4 | 48.3 |
|  | Female | 582 | 11.8 | 7.5 | 16.1 | 6.0 | 3.0 | 9.0 | 43.7 | 36.8 | 50.6 |
| **Maternal age (years)** | 15 -24 | 334 | 11.4 | 5.5 | 17.2 | 4.0 | 1.1 | 6.8 | 48.0 | 39.8 | 56.2 |
|  | 25 - 29 | 346 | 12.8 | 7.4 | 18.3 | 3.5 | 0.5 | 6.4 | 40.9 | 31.4 | 50.4 |
|  | 30 - 34 | 266 | 18.6 | 10.6 | 26.6 | 8.8 | 2.1 | 15.4 | 36.6 | 27.7 | 45.5 |
|  | 35 - 39 | 222 | 14.7 | 7.8 | 21.7 | 8.2 | 2.9 | 13.5 | 45.3 | 32.7 | 57.9 |
|  | 40 - 49 | 106 | 14.4 | 4.8 | 24.0 | 3.5 | 0.3 | 6.8 | 44.8 | 31.4 | 58.3 |
| **Maternal education** | No education | 750 | 13.4 | 9.3 | 17.6 | 5.2 | 2.5 | 7.8 | 43.3 | 37.5 | 49.1 |
|  | Primary | 348 | 15.8 | 9.7 | 21.9 | 4.7 | 1.0 | 8.4 | 43.5 | 33.8 | 53.3 |
|  | Secondary or higher | 176 | 13.7 | 6.2 | 21.2 | 8.7 | 2.2 | 15.1 | 40.3 | 25.8 | 54.8 |
| **Residence** | Urban | 720 | 15.5 | 9.5 | 21.5 | 6.5 | 2.5 | 10.6 | 43.5 | 34.9 | 52.1 |
|  | Rural | 555 | 12.3 | 9.3 | 15.4 | 4.2 | 2.1 | 6.3 | 42.3 | 37.0 | 47.5 |
| **Household wealth** | Poorest | 185 | 12.8 | 8.5 | 17.0 | 3.9 | 1.6 | 6.3 | 37.8 | 30.0 | 45.6 |
|  | Second | 181 | 12.5 | 7.9 | 17.2 | 3.6 | 0.4 | 6.8 | 46.4 | 38.2 | 54.6 |
|  | Middle | 221 | 13.6 | 6.7 | 20.5 | 3.9 | 1.0 | 6.8 | 47.2 | 36.6 | 57.8 |
|  | Fourth | 347 | 11.9 | 5.3 | 18.5 | 5.4 | 1.7 | 9.1 | 40.2 | 29.0 | 51.3 |
|  | Least poor | 341 | 18.2 | 8.9 | 27.5 | 8.6 | 0.9 | 16.4 | 44.0 | 34.8 | 53.3 |
| **Household members** | 1-4 members | 61 | 28.2 | 11.3 | 45.1 | 15.2 | 2.9 | 27.5 | 41.8 | 28.7 | 55.0 |
|  | 5-8 members | 275 | 12.6 | 7.4 | 17.8 | 3.4 | 0.4 | 6.3 | 46.0 | 36.3 | 55.6 |
|  | 9-12 members | 292 | 15.3 | 9.9 | 20.7 | 4.8 | 1.8 | 7.9 | 41.7 | 32.9 | 50.4 |
|  | 13 or more members | 647 | 12.9 | 7.9 | 17.8 | 5.8 | 2.4 | 9.2 | 42.4 | 34.3 | 50.4 |
| **Health care access (money)** | Big problem | 676 | 13.2 | 8.9 | 17.4 | 5.4 | 2.8 | 8.0 | 42.5 | 35.1 | 49.8 |
|  | Not a big problem | 599 | 15.2 | 10.9 | 19.5 | 5.7 | 2.5 | 8.9 | 43.5 | 37.0 | 50.1 |
| **Health care access (distance)** | Big problem | 350 | 13.4 | 9.2 | 17.5 | 4.8 | 2.0 | 7.6 | 47.2 | 38.8 | 55.5 |
|  | Not a big problem | 924 | 14.4 | 10.0 | 18.8 | 5.8 | 2.5 | 9.1 | 41.4 | 34.6 | 48.1 |
| **Symptoms** | Fever alone | 527 | 15.5 | 11.3 | 19.8 | 5.6 | 2.7 | 8.4 | 36.8 | 30.0 | 43.7 |
|  | Fever, cough | 240 | 11.3 | 6.0 | 16.7 | 3.6 | 0.7 | 6.5 | 41.0 | 31.8 | 50.3 |
|  | Fever, cough, rapid breaths | 508 | 13.9 | 9.2 | 18.7 | 6.4 | 2.5 | 10.3 | 50.2 | 42.2 | 58.2 |
| **Malaria transmission season** | Off-peak | 781 | 12.0 | 9.6 | 14.5 | 4.1 | 2.4 | 5.8 | 43.7 | 38.5 | 48.9 |
|  | Peak | 487 | 17.5 | 8.7 | 26.4 | 7.9 | 1.9 | 13.9 | 41.6 | 30.4 | 52.7 |
| **Health card** | No (never had or lost) | 37 | 15.2 | 4.2 | 26.2 | 9.4 | 0.4 | 18.3 | 37.5 | 19.3 | 55.8 |
|  | Yes (seen or reported) | 1,238 | 14.1 | 10.4 | 17.7 | 5.4 | 2.9 | 7.9 | 43.1 | 37.5 | 48.7 |

Point estimates tabulated using sample weights pre-specified in datasets. Standard error estimation accounted for data clustering in survey designs.

**Uganda DHS 2011**

|  |  | **N febrile under-fives taken to any care** | **Percent receiving any anti-malarial** | **95% CI** | | **Percent receiving ACT** | **95% CI** | | **Percent receiving any antibiotic** | **95% CI** | |
| --- | --- | --- | --- | --- | --- | --- | --- | --- | --- | --- | --- |
|  |  |  |  |  |  |  |  |  |  |  |  |
| **Diagnostic test use** | No | 1,726 | 67.6 | 64.1 | 71.1 | 45.5 | 41.3 | 49.7 | 33.1 | 29.7 | 36.5 |
|  | Yes | 697 | 76.4 | 71.9 | 80.8 | 52.7 | 47.2 | 58.1 | 40.9 | 35.4 | 46.5 |
| **Malaria endemicity** | Malaria-free | 23 | 29.6 | 17.0 | 42.3 | 10.5 | 1.7 | 19.3 | 23.8 | 1.7 | 45.9 |
|  | Unstable risk | 0 | - | - | - | - | - | - | - | - | - |
|  | Low risk (*Pf*PR_2–10_ <5%) | 1 | - | - | - | - | - | - | - | - | - |
|  | Moderate risk (*Pf*PR_2–10_ 5-40%) | 625 | 73.1 | 68.0 | 78.1 | 51.6 | 46.1 | 57.1 | 33.0 | 28.1 | 37.9 |
|  | High risk (*Pf*PR_2–10_ >40%) | 1,730 | 68.8 | 65.2 | 72.4 | 46.1 | 41.4 | 50.9 | 36.6 | 32.8 | 40.3 |
| **Source (public/private)** | Public | 965 | 78.1 | 74.5 | 81.6 | 60.4 | 55.9 | 64.8 | 31.7 | 27.9 | 35.5 |
|  | Private | 1,474 | 64.5 | 60.5 | 68.5 | 39.0 | 34.0 | 44.0 | 37.6 | 33.6 | 41.6 |
| **Source (level)** | Hospital | 1,424 | 66.8 | 63.1 | 70.5 | 40.3 | 35.4 | 45.1 | 38.8 | 34.8 | 42.8 |
|  | Non-hospital formal medical | 809 | 77.7 | 74.0 | 81.5 | 61.8 | 57.3 | 66.3 | 30.9 | 27.0 | 34.9 |
|  | Community health worker | 36 | 86.6 | 73.9 | 99.3 | 79.7 | 63.4 | 95.9 | 26.5 | 4.2 | 48.7 |
|  | Pharmacy | 47 | 56.8 | 37.6 | 75.9 | 23.0 | 8.4 | 37.6 | 21.2 | 6.9 | 35.5 |
|  | Other | 124 | 53.4 | 39.7 | 67.1 | 36.3 | 22.8 | 49.7 | 30.5 | 19.0 | 42.0 |
| **Child's age (months)** | 0 - 5 | 172 | 35.6 | 26.7 | 44.5 | 17.3 | 9.9 | 24.7 | 50.9 | 41.2 | 60.6 |
|  | 6 - 11 | 312 | 66.3 | 60.4 | 72.2 | 40.9 | 34.5 | 47.4 | 41.3 | 35.0 | 47.7 |
|  | 12 - 23 | 557 | 73.4 | 68.4 | 78.4 | 51.7 | 45.8 | 57.7 | 34.0 | 29.5 | 38.5 |
|  | 24 - 35 | 532 | 73.8 | 69.3 | 78.2 | 50.7 | 44.8 | 56.6 | 35.9 | 30.9 | 40.9 |
|  | 36 - 47 | 450 | 72.5 | 67.6 | 77.3 | 52.4 | 46.5 | 58.2 | 31.1 | 24.8 | 37.5 |
|  | 48 - 59 | 418 | 74.1 | 68.1 | 80.2 | 49.7 | 42.9 | 56.6 | 29.6 | 22.7 | 36.4 |
| **Child's sex** | Male | 1,168 | 68.3 | 64.7 | 72.0 | 47.4 | 43.1 | 51.7 | 34.2 | 30.8 | 37.6 |
|  | Female | 1,272 | 71.3 | 67.7 | 74.8 | 47.5 | 43.4 | 51.7 | 36.2 | 32.1 | 40.4 |
| **Maternal age (years)** | 15 -24 | 759 | 68.5 | 64.1 | 72.9 | 45.3 | 40.7 | 49.9 | 34.7 | 29.7 | 39.7 |
|  | 25 - 29 | 708 | 69.9 | 64.9 | 75.0 | 43.7 | 38.0 | 49.4 | 38.1 | 33.2 | 42.9 |
|  | 30 - 34 | 433 | 68.3 | 62.1 | 74.5 | 50.4 | 43.0 | 57.8 | 33.4 | 27.4 | 39.4 |
|  | 35 - 39 | 332 | 69.6 | 63.3 | 75.8 | 51.2 | 44.2 | 58.1 | 33.9 | 26.2 | 41.6 |
|  | 40 - 49 | 208 | 78.1 | 69.7 | 86.6 | 56.3 | 44.6 | 68.0 | 33.9 | 22.8 | 45.1 |
| **Maternal education** | No education | 322 | 64.5 | 57.0 | 72.1 | 46.7 | 38.3 | 55.1 | 31.9 | 25.1 | 38.7 |
|  | Primary | 1,637 | 71.7 | 68.3 | 75.1 | 47.1 | 42.6 | 51.7 | 33.7 | 29.8 | 37.5 |
|  | Secondary or higher | 480 | 67.0 | 61.8 | 72.2 | 49.1 | 43.0 | 55.2 | 43.0 | 37.5 | 48.5 |
| **Residence** | Urban | 285 | 64.6 | 58.0 | 71.2 | 44.7 | 38.1 | 51.3 | 45.6 | 38.6 | 52.6 |
|  | Rural | 2,155 | 70.6 | 67.5 | 73.6 | 47.8 | 43.8 | 51.9 | 33.9 | 30.6 | 37.2 |
| **Household wealth** | Poorest | 649 | 71.2 | 66.4 | 76.0 | 46.0 | 39.6 | 52.5 | 30.4 | 25.4 | 35.5 |
|  | Second | 538 | 74.6 | 69.0 | 80.2 | 51.2 | 44.3 | 58.1 | 30.7 | 25.4 | 36.0 |
|  | Middle | 447 | 67.6 | 62.0 | 73.1 | 46.3 | 39.9 | 52.6 | 36.1 | 30.0 | 42.3 |
|  | Fourth | 436 | 65.4 | 58.5 | 72.2 | 44.9 | 37.6 | 52.1 | 37.6 | 31.5 | 43.8 |
|  | Least poor | 370 | 68.7 | 62.1 | 75.3 | 49.1 | 42.5 | 55.7 | 46.5 | 39.5 | 53.6 |
| **Household members** | 1-4 members | 567 | 68.1 | 63.2 | 73.0 | 44.4 | 38.8 | 50.0 | 36.2 | 30.7 | 41.7 |
|  | 5-8 members | 1,315 | 69.8 | 66.0 | 73.7 | 46.6 | 41.8 | 51.5 | 33.7 | 29.9 | 37.6 |
|  | 9-12 members | 486 | 72.2 | 67.0 | 77.3 | 55.6 | 49.8 | 61.5 | 35.5 | 29.6 | 41.5 |
|  | 13 or more members | 71 | 68.9 | 58.2 | 79.6 | 31.3 | 17.7 | 44.9 | 54.3 | 36.5 | 72.1 |
| **Health care access (money)** | Big problem | 1,278 | 70.9 | 67.1 | 74.7 | 49.2 | 44.5 | 53.9 | 34.7 | 30.8 | 38.5 |
|  | Not a big problem | 1,161 | 68.8 | 65.1 | 72.4 | 45.6 | 41.3 | 49.9 | 35.9 | 31.9 | 39.9 |
| **Health care access (distance)** | Big problem | 1,168 | 68.8 | 64.5 | 73.0 | 45.5 | 40.7 | 50.3 | 34.3 | 29.6 | 38.9 |
|  | Not a big problem | 1,272 | 70.9 | 67.1 | 74.6 | 49.3 | 44.6 | 53.9 | 36.2 | 32.8 | 39.6 |
| **Symptoms** | Fever alone | 798 | 72.1 | 67.9 | 76.4 | 50.3 | 44.9 | 55.7 | 24.6 | 20.4 | 28.8 |
|  | Fever, cough | 779 | 71.2 | 66.9 | 75.4 | 49.7 | 44.7 | 54.7 | 37.3 | 32.5 | 42.1 |
|  | Fever, cough, rapid breaths | 863 | 66.6 | 61.7 | 71.4 | 42.8 | 37.9 | 47.8 | 43.3 | 38.1 | 48.6 |
| **Malaria transmission season** | Off-peak | 239 | 62.0 | 53.8 | 70.3 | 39.9 | 31.8 | 48.0 | 33.9 | 25.3 | 42.5 |
|  | Peak | 2,142 | 70.3 | 67.3 | 73.4 | 48.0 | 44.0 | 52.1 | 35.7 | 32.5 | 38.9 |
| **Health card** | No (never had or lost) | 380 | 67.1 | 59.6 | 73.9 | 42.8 | 35.5 | 50.1 | 34.1 | 27.9 | 40.3 |
|  | Yes (seen or reported) | 2,060 | 70.4 | 67.5 | 73.1 | 48.3 | 44.5 | 52.2 | 35.5 | 32.1 | 38.8 |

Point estimates tabulated using sample weights pre-specified in datasets. Standard error estimation accounted for data clustering in survey designs.

**Zimbabwe DHS 2010-2011**

|  |  | **N febrile under-fives taken to any care** | **Percent receiving any anti-malarial** | **95% CI** | | **Percent receiving ACT** | **95% CI** | | **Percent receiving any antibiotic** | **95% CI** | |
| --- | --- | --- | --- | --- | --- | --- | --- | --- | --- | --- | --- |
|  |  |  |  |  |  |  |  |  |  |  |  |
| **Diagnostic test use** | No | 189 | 2.8 | 0.3 | 5.2 | 1.4 | -0.6 | 3.5 | 40.1 | 32.7 | 47.5 |
|  | Yes | 27 | 20.5 | 3.8 | 37.1 | 10.9 | -2.0 | 23.8 | 30.2 | 12.1 | 48.3 |
| **Malaria endemicity** | Malaria-free | 3 | - | - | - | - | - | - | - | - | - |
|  | Unstable risk | 0 | - | - | - | - | - | - | - | - | - |
|  | Low risk (*Pf*PR_2–10_ <5%) | 78 | 1.8 | -0.9 | 4.6 | 1.3 | -1.3 | 3.9 | 34.1 | 22.9 | 45.4 |
|  | Moderate risk (*Pf*PR_2–10_ 5-40%) | 128 | 7.3 | 1.2 | 13.4 | 3.6 | -0.1 | 7.4 | 41.8 | 32.8 | 50.9 |
|  | High risk (*Pf*PR_2–10_ >40%) | 0 | - | - | - | - | - | - | - | - | - |
| **Source (public/private)** | Public | 159 | 4.7 | 0.3 | 9.1 | 1.5 | -0.7 | 3.6 | 40.8 | 32.9 | 48.7 |
|  | Private | 58 | 5.8 | -0.8 | 12.5 | 5.8 | -0.8 | 12.5 | 33.4 | 20.9 | 46.0 |
| **Source (level)** | Hospital | 57 | 10.7 | -0.7 | 22.1 | 7.1 | -0.6 | 14.7 | 32.5 | 19.7 | 45.3 |
|  | Non-hospital formal medical | 124 | 2.4 | -0.3 | 5.2 | - | - | - | 43.8 | 34.9 | 52.7 |
|  | Community health worker | 1 | - | - | - | - | - | - | - | - | - |
|  | Pharmacy | 17 | 6.1 | -5.5 | 17.6 | 6.1 | -5.5 | 17.6 | 55.3 | 33.6 | 77.1 |
|  | Other | 17 | - | - | - | - | - | - | 9.6 | -8.3 | 27.4 |
| **Child's age (months)** | 0 - 5 | 20 | - | - | - | - | - | - | 37.6 | 17.5 | 57.7 |
|  | 6 - 11 | 48 | 2.1 | -2.0 | 6.3 | 2.1 | -2.0 | 6.3 | 41.7 | 27.7 | 55.7 |
|  | 12 - 23 | 41 | 5.1 | -2.1 | 12.2 | 0.0 | 0.0 | 0.0 | 38.0 | 23.6 | 52.3 |
|  | 24 - 35 | 43 | 3.6 | -1.5 | 8.6 | 1.4 | -1.4 | 4.2 | 42.3 | 25.1 | 59.5 |
|  | 36 - 47 | 38 | 12.6 | 0.2 | 25.1 | 10.6 | -1.3 | 22.5 | 31.1 | 15.2 | 46.9 |
|  | 48 - 59 | 25 | 5.2 | -4.8 | 15.2 | - | - | - | 41.8 | 22.5 | 61.0 |
| **Child's sex** | Male | 107 | 4.1 | -0.5 | 8.7 | 2.5 | -1.0 | 6.0 | 38.8 | 29.0 | 48.7 |
|  | Female | 110 | 5.9 | 1.5 | 10.2 | 2.7 | -0.7 | 6.1 | 38.8 | 29.2 | 48.5 |
| **Maternal age (years)** | 15 -24 | 93 | 4.4 | -2.7 | 11.5 | 2.6 | -1.2 | 6.3 | 39.8 | 29.0 | 50.6 |
|  | 25 - 29 | 64 | 5.7 | -0.2 | 11.7 | 1.6 | -1.5 | 4.7 | 35.7 | 23.9 | 47.5 |
|  | 30 - 34 | 29 | 7.8 | -3.6 | 19.2 | 7.8 | -3.6 | 19.2 | 47.9 | 28.9 | 66.8 |
|  | 35 - 39 | 24 | 3.2 | -3.1 | 9.6 | - | - | - | 35.5 | 16.1 | 54.9 |
|  | 40 - 49 | 7 | - | - | - | - | - | - | 27.6 | -1.2 | 56.4 |
| **Maternal education** | No education | 4 | 19.1 | -15.2 | 53.3 | - | - | - | 56.1 | 14.3 | 97.9 |
|  | Primary | 72 | 5.4 | -0.4 | 11.3 | 2.3 | -2.1 | 6.8 | 37.5 | 25.5 | 49.5 |
|  | Secondary or higher | 141 | 4.3 | -0.4 | 9.1 | 2.9 | -0.1 | 5.8 | 39.0 | 30.3 | 47.7 |
| **Residence** | Urban | 57 | 8.4 | -2.5 | 19.4 | 4.8 | -1.7 | 11.2 | 34.4 | 20.3 | 48.5 |
|  | Rural | 160 | 3.8 | 0.7 | 6.8 | 1.9 | -0.4 | 4.2 | 40.4 | 32.5 | 48.3 |
| **Household wealth** | Poorest | 55 | 1.3 | -1.2 | 3.7 | 1.3 | -1.2 | 3.7 | 35.7 | 23.4 | 48.1 |
|  | Second | 45 | 8.8 | -0.5 | 18.0 | 3.7 | -3.5 | 11.0 | 38.8 | 23.5 | 54.2 |
|  | Middle | 41 | 1.9 | -1.8 | 5.6 | - | - | - | 50.4 | 35.2 | 65.6 |
|  | Fourth | 41 | 4.0 | -1.7 | 9.7 | 4.0 | -1.7 | 9.7 | 41.1 | 25.4 | 56.8 |
|  | Least poor | 34 | 11.0 | -5.8 | 27.9 | 4.9 | -4.1 | 13.9 | 27.2 | 9.2 | 45.3 |
| **Household members** | 1-4 members | 85 | 4.7 | -0.2 | 9.6 | 4.7 | -0.2 | 9.6 | 42.1 | 30.4 | 53.7 |
|  | 5-8 members | 104 | 6.5 | 1.3 | 11.7 | 1.6 | -1.5 | 4.7 | 35.9 | 26.1 | 45.7 |
|  | 9-12 members | 21 | - | - | - | - | - | - | 47.3 | 25.1 | 69.5 |
|  | 13 or more members | 6 | - | - | - | - | - | - | 16.5 | -7.5 | 40.5 |
| **Health care access (money)** | Big problem | 127 | 2.8 | -0.4 | 6.1 | 1.8 | -0.9 | 4.5 | 40.9 | 31.8 | 50.0 |
|  | Not a big problem | 89 | 8.1 | 0.4 | 15.7 | 3.8 | -0.6 | 8.2 | 35.9 | 25.2 | 46.6 |
| **Health care access (distance)** | Big problem | 94 | 3.2 | 0.1 | 6.4 | 1.4 | -0.5 | 3.3 | 42.5 | 32.1 | 52.8 |
|  | Not a big problem | 123 | 6.3 | 0.2 | 12.4 | 3.6 | -0.5 | 7.6 | 36.1 | 26.6 | 45.5 |
| **Symptoms** | Fever alone | 88 | 9.2 | 0.8 | 17.6 | 5.3 | 0.0 | 10.6 | 31.7 | 22.0 | 41.4 |
|  | Fever, cough | 60 | 3.9 | -1.5 | 9.3 | 1.7 | -1.6 | 5.0 | 45.9 | 32.7 | 59.0 |
|  | Fever, cough, rapid breaths | 69 | 0.6 | -0.6 | 1.7 | - | - | - | 41.8 | 29.7 | 54.0 |
| **Malaria transmission season** | Off-peak | 161 | 4.9 | 0.5 | 9.2 | 2.5 | -0.1 | 5.1 | 39.3 | 31.4 | 47.3 |
|  | Peak | 49 | 6.1 | -1.3 | 13.6 | 3.4 | -3.0 | 9.8 | 35.2 | 20.8 | 49.7 |
| **Health card** | No (never had or lost) | 22 | 12.1 | -4.1 | 28.2 | 7.8 | -6.7 | 22.2 | 27.6 | 7.7 | 47.5 |
|  | Yes (seen or reported) | 195 | 4.2 | 1.2 | 7.2 | 2.1 | -0.1 | 4.2 | 40.1 | 32.7 | 47.5 |

Point estimates tabulated using sample weights pre-specified in datasets. Standard error estimation accounted for data clustering in survey designs.
