# Supplementary material for: Effect of diagnostic testing on medicines used by febrile children less than five years in 12 malaria-endemic African countries: a mixed-methods study
Source: Malar J. 2015 May 10;14:194. doi: 10.1186/s12936-015-0709-0 (PMC4432948; doi:10.1186/s12936-015-0709-0)
Supplement: Additional file 2: — Effect of diagnostic testing and other covariates on medicines used by febrile children under five years taken to any care in 12 countries. CI = confidence interval; AOR = adjusted odds ratio. AORs based on mixed-effects logistic regression models adjusted for data clustering and all listed covariates. [file 12936_2015_709_MOESM2_ESM.docx]

**Additional File 2: Effect of diagnostic testing and other covariates on medicines used by febrile children less than five years taken to any care in 12 countries**

**Benin DHS 2011-2012**

|  |  | **Any anti-malarial use** | | | | **ACT Use** | | | | **Any antibiotic use** | | | |
| --- | --- | --- | --- | --- | --- | --- | --- | --- | --- | --- | --- | --- | --- |
|  |  | **AOR** | **95% CI** | | **pvalue** | **AOR** | **95% CI** | | **pvalue** | **AOR** | **95% CI** | | **pvalue** |
| **Diagnostic test use** | No | 1.00 |  |  |  | 1.00 |  |  |  | 1.00 |  |  |  |
|  | Yes | 1.65 | 0.92 | 2.98 | 0.096 | 1.96 | 0.91 | 4.19 | 0.084 | 1.15 | 0.64 | 2.08 | 0.636 |
| **Malaria endemicity** | Moderate risk (*Pf*PR_2–10_ 5-40%) | 1.00 |  |  |  | 1.00 |  |  |  | 1.00 |  |  |  |
|  | High risk (*Pf*PR_2–10_ >40%) | 1.25 | 0.54 | 2.87 | 0.600 | 4.64 | 1.22 | 17.68 | 0.025 | 0.72 | 0.31 | 1.70 | 0.459 |
| **Source (public/private)** | Public | 1.00 |  |  |  | 1.00 |  |  |  | 1.00 |  |  |  |
|  | Private | 0.24 | 0.10 | 0.61 | 0.003 | 0.27 | 0.06 | 1.14 | 0.076 | 0.90 | 0.37 | 2.21 | 0.826 |
| **Source (level)** | Hospital | 1.00 |  |  |  | 1.00 |  |  |  | 1.00 |  |  |  |
|  | Non-hospital formal medical | 0.85 | 0.43 | 1.67 | 0.631 | 1.75 | 0.73 | 4.23 | 0.212 | 1.03 | 0.53 | 1.98 | 0.931 |
|  | Community health worker | 1.01 | 0.27 | 3.75 | 0.990 | 1.00 | 0.18 | 5.55 | 0.999 | 0.31 | 0.07 | 1.45 | 0.138 |
|  | Pharmacy | 1.98 | 0.66 | 5.91 | 0.220 | 3.86 | 0.71 | 21.00 | 0.118 | 0.55 | 0.18 | 1.70 | 0.298 |
|  | Other | 0.70 | 0.28 | 1.71 | 0.430 | 0.84 | 0.19 | 3.68 | 0.816 | 0.09 | 0.03 | 0.26 | <0.001 |
| **Child's age (months)** | 0 - 5 | 0.33 | 0.13 | 0.86 | 0.023 | 0.20 | 0.04 | 0.89 | 0.035 | 1.19 | 0.47 | 3.01 | 0.711 |
|  | 6 - 11 | 1.00 |  |  |  | 1.00 |  |  |  | 1.00 |  |  |  |
|  | 12 - 23 | 1.04 | 0.52 | 2.08 | 0.916 | 0.65 | 0.25 | 1.66 | 0.369 | 0.69 | 0.34 | 1.43 | 0.322 |
|  | 24 - 35 | 1.26 | 0.60 | 2.62 | 0.545 | 0.77 | 0.28 | 2.07 | 0.601 | 0.36 | 0.16 | 0.82 | 0.015 |
|  | 36 - 47 | 1.03 | 0.45 | 2.31 | 0.952 | 1.84 | 0.63 | 5.34 | 0.261 | 0.61 | 0.25 | 1.51 | 0.289 |
|  | 48 - 59 | 1.68 | 0.73 | 3.86 | 0.221 | 1.06 | 0.35 | 3.23 | 0.923 | 0.45 | 0.18 | 1.18 | 0.104 |
| **Child's sex** | Male | 1.00 |  |  |  | 1.00 |  |  |  | 1.00 |  |  |  |
|  | Female | 1.09 | 0.71 | 1.68 | 0.698 | 0.85 | 0.46 | 1.55 | 0.592 | 0.96 | 0.60 | 1.53 | 0.851 |
| **Maternal age (years)** | 15 -24 | 1.00 |  |  |  | 1.00 |  |  |  | 1.00 |  |  |  |
|  | 25 - 29 | 1.19 | 0.65 | 2.15 | 0.577 | 0.99 | 0.44 | 2.24 | 0.989 | 0.74 | 0.39 | 1.39 | 0.347 |
|  | 30 - 34 | 0.56 | 0.28 | 1.11 | 0.096 | 1.24 | 0.49 | 3.18 | 0.649 | 1.33 | 0.64 | 2.73 | 0.445 |
|  | 35 - 39 | 1.01 | 0.47 | 2.21 | 0.972 | 0.68 | 0.23 | 1.99 | 0.485 | 0.80 | 0.35 | 1.84 | 0.605 |
|  | 40 - 49 | 1.46 | 0.58 | 3.72 | 0.424 | 1.52 | 0.45 | 5.09 | 0.499 | 0.54 | 0.17 | 1.64 | 0.274 |
| **Maternal education** | No education | 1.00 |  |  |  | 1.00 |  |  |  | 1.00 |  |  |  |
|  | Primary | 1.39 | 0.79 | 2.47 | 0.256 | 1.38 | 0.63 | 3.01 | 0.421 | 1.20 | 0.66 | 2.18 | 0.550 |
|  | Secondary or higher | 1.18 | 0.55 | 2.51 | 0.672 | 1.97 | 0.71 | 5.47 | 0.194 | 2.16 | 0.98 | 4.76 | 0.057 |
| **Residence** | Urban | 1.00 |  |  |  | 1.00 |  |  |  | 1.00 |  |  |  |
|  | Rural | 0.72 | 0.41 | 1.28 | 0.267 | 0.85 | 0.39 | 1.85 | 0.685 | 0.69 | 0.37 | 1.26 | 0.229 |
| **Household wealth** | Poorest | 1.00 |  |  |  | 1.00 |  |  |  | 1.00 |  |  |  |
|  | Second | 1.72 | 0.78 | 3.77 | 0.178 | 1.84 | 0.59 | 5.75 | 0.295 | 1.49 | 0.59 | 3.77 | 0.401 |
|  | Middle | 1.44 | 0.66 | 3.13 | 0.364 | 5.35 | 1.62 | 17.69 | 0.006 | 2.83 | 1.11 | 7.20 | 0.029 |
|  | Fourth | 0.79 | 0.35 | 1.81 | 0.583 | 2.80 | 0.84 | 9.31 | 0.093 | 3.79 | 1.43 | 10.04 | 0.007 |
|  | Least poor | 1.37 | 0.50 | 3.76 | 0.546 | 2.77 | 0.65 | 11.90 | 0.170 | 1.80 | 0.58 | 5.57 | 0.306 |
| **Household members** | 1-4 members | 1.00 |  |  |  | 1.00 |  |  |  | 1.00 |  |  |  |
|  | 5-8 members | 0.99 | 0.57 | 1.72 | 0.970 | 0.76 | 0.35 | 1.66 | 0.493 | 1.06 | 0.59 | 1.91 | 0.845 |
|  | 9-12 members | 1.55 | 0.74 | 3.21 | 0.243 | 1.26 | 0.49 | 3.23 | 0.631 | 0.78 | 0.35 | 1.73 | 0.535 |
|  | 13 or more members | 1.73 | 0.58 | 5.12 | 0.324 | 0.29 | 0.05 | 1.56 | 0.149 | 1.46 | 0.46 | 4.62 | 0.521 |
| **Health care access (money)** | Big problem | 1.00 |  |  |  | 1.00 |  |  |  | 1.00 |  |  |  |
|  | Not a big problem | 1.08 | 0.64 | 1.83 | 0.764 | 0.89 | 0.44 | 1.81 | 0.749 | 0.51 | 0.28 | 0.91 | 0.023 |
| **Health care access (distance)** | Big problem | 1.00 |  |  |  | 1.00 |  |  |  | 1.00 |  |  |  |
|  | Not a big problem | 0.94 | 0.55 | 1.58 | 0.802 | 1.45 | 0.70 | 3.00 | 0.323 | 1.15 | 0.65 | 2.02 | 0.629 |
| **Symptoms** | Fever alone | 1.00 |  |  |  | 1.00 |  |  |  | 1.00 |  |  |  |
|  | Fever, cough | 0.83 | 0.48 | 1.43 | 0.508 | 0.67 | 0.31 | 1.43 | 0.302 | 2.64 | 1.47 | 4.75 | 0.001 |
|  | Fever, cough, rapid breaths | 1.79 | 0.94 | 3.42 | 0.078 | 0.32 | 0.12 | 0.86 | 0.025 | 1.57 | 0.79 | 3.11 | 0.196 |
| **Malaria transmission season** | Off-peak | 1.00 |  |  |  | 1.00 |  |  |  | 1.00 |  |  |  |
|  | Peak | 0.74 | 0.30 | 1.81 | 0.504 | 1.65 | 0.41 | 6.58 | 0.476 | 0.90 | 0.36 | 2.24 | 0.814 |
| **Health card** | No (never had or lost) | 1.00 |  |  |  | 1.00 |  |  |  | 1.00 |  |  |  |
|  | Yes (seen or reported) | 2.74 | 1.21 | 6.17 | 0.015 | 1.64 | 0.52 | 5.12 | 0.397 | 0.52 | 0.22 | 1.25 | 0.143 |

CI refers to confidence interval. AOR refers to adjusted odds ratio. AORs based on mixed-effects logistic regression models adjusted for data clustering and all listed covariates. Some covariates may have been included in model with collapsed response categories, and if so, this is noted in the above table.

**Burkina Faso DHS 2010-2011**

|  |  | **Any anti-malarial use** | | | | **ACT Use** | | | | **Any antibiotic use** | | | |
| --- | --- | --- | --- | --- | --- | --- | --- | --- | --- | --- | --- | --- | --- |
|  |  | **AOR** | **95% CI** | | **pvalue** | **AOR** | **95% CI** | | **pvalue** | **AOR** | **95% CI** | | **pvalue** |
| **Diagnostic test use** | No | 1.00 |  |  |  | 1.00 |  |  |  | 1.00 |  |  |  |
|  | Yes | 1.32 | 0.84 | 2.05 | 0.225 | 1.45 | 0.84 | 2.52 | 0.180 | 0.89 | 0.57 | 1.40 | 0.616 |
| **Malaria endemicity** | High risk (*Pf*PR_2–10_ >40%) | 1.00 |  |  |  | 1.00 |  |  |  | 1.00 |  |  |  |
| **Source (public/private)** | Public | 1.00 |  |  |  | 1.00 |  |  |  | 1.00 |  |  |  |
|  | Private | 0.16 | 0.05 | 0.52 | 0.003 | 0.28 | 0.04 | 1.96 | 0.201 | 0.82 | 0.24 | 2.85 | 0.753 |
| **Source (level)** | Hospital | 1.00 |  |  |  | 1.00 |  |  |  | 1.00 |  |  |  |
|  | Non-hospital formal medical | 0.90 | 0.62 | 1.32 | 0.600 | 0.97 | 0.60 | 1.58 | 0.915 | 1.05 | 0.71 | 1.55 | 0.819 |
|  | Community health worker | 0.75 | 0.14 | 4.08 | 0.738 | 0.83 | 0.05 | 14.83 | 0.900 | 0.81 | 0.16 | 4.07 | 0.795 |
|  | Pharmacy | 1.02 | 0.26 | 3.94 | 0.978 | 1.61 | 0.18 | 14.04 | 0.667 | 0.39 | 0.09 | 1.58 | 0.186 |
|  | Other | 0.24 | 0.07 | 0.87 | 0.030 | 0.16 | 0.02 | 1.39 | 0.096 | 0.05 | 0.01 | 0.18 | <0.001 |
| **Child's age (months)** | 0 - 5 | 0.46 | 0.26 | 0.81 | 0.007 | 0.57 | 0.25 | 1.32 | 0.191 | 1.28 | 0.72 | 2.28 | 0.392 |
|  | 6 - 11 | 1.00 |  |  |  | 1.00 |  |  |  | 1.00 |  |  |  |
|  | 12 - 23 | 1.13 | 0.79 | 1.61 | 0.506 | 1.00 | 0.61 | 1.62 | 0.986 | 0.87 | 0.60 | 1.27 | 0.472 |
|  | 24 - 35 | 1.10 | 0.75 | 1.62 | 0.610 | 0.71 | 0.42 | 1.20 | 0.200 | 1.07 | 0.72 | 1.60 | 0.725 |
|  | 36 - 47 | 2.15 | 1.40 | 3.28 | <0.001 | 1.12 | 0.63 | 1.97 | 0.700 | 0.48 | 0.31 | 0.75 | 0.001 |
|  | 48 - 59 | 1.67 | 1.02 | 2.75 | 0.042 | 1.16 | 0.61 | 2.23 | 0.648 | 0.52 | 0.31 | 0.88 | 0.015 |
| **Child's sex** | Male | 1.00 |  |  |  | 1.00 |  |  |  | 1.00 |  |  |  |
|  | Female | 1.12 | 0.89 | 1.42 | 0.333 | 0.89 | 0.65 | 1.24 | 0.495 | 0.81 | 0.64 | 1.04 | 0.094 |
| **Maternal age (years)** | 15 -24 | 1.00 |  |  |  | 1.00 |  |  |  | 1.00 |  |  |  |
|  | 25 - 29 | 0.62 | 0.45 | 0.86 | 0.004 | 0.59 | 0.38 | 0.91 | 0.018 | 1.46 | 1.03 | 2.05 | 0.031 |
|  | 30 - 34 | 0.77 | 0.53 | 1.11 | 0.160 | 0.50 | 0.31 | 0.82 | 0.006 | 1.08 | 0.74 | 1.59 | 0.676 |
|  | 35 - 39 | 1.04 | 0.69 | 1.58 | 0.850 | 0.37 | 0.20 | 0.68 | 0.001 | 1.45 | 0.94 | 2.23 | 0.091 |
|  | 40 - 49 | 0.71 | 0.44 | 1.17 | 0.179 | 0.51 | 0.26 | 1.02 | 0.058 | 1.60 | 0.96 | 2.67 | 0.073 |
| **Maternal education** | No education | 1.00 |  |  |  | 1.00 |  |  |  | 1.00 |  |  |  |
|  | Primary | 1.52 | 1.07 | 2.14 | 0.018 | 1.32 | 0.84 | 2.07 | 0.232 | 0.77 | 0.53 | 1.10 | 0.146 |
|  | Secondary or higher | 1.75 | 1.04 | 2.95 | 0.034 | 1.87 | 1.02 | 3.44 | 0.043 | 0.78 | 0.46 | 1.32 | 0.351 |
| **Residence** | Urban | 1.00 |  |  |  | 1.00 |  |  |  | 1.00 |  |  |  |
|  | Rural | 0.91 | 0.61 | 1.37 | 0.667 | 0.80 | 0.47 | 1.36 | 0.409 | 1.25 | 0.80 | 1.93 | 0.324 |
| **Household wealth** | Poorest | 1.00 |  |  |  | 1.00 |  |  |  | 1.00 |  |  |  |
|  | Second | 0.60 | 0.39 | 0.92 | 0.021 | 0.62 | 0.33 | 1.19 | 0.154 | 1.66 | 1.05 | 2.63 | 0.031 |
|  | Middle | 0.76 | 0.50 | 1.16 | 0.207 | 0.67 | 0.37 | 1.24 | 0.202 | 1.26 | 0.81 | 1.96 | 0.299 |
|  | Fourth | 0.78 | 0.51 | 1.20 | 0.260 | 1.18 | 0.65 | 2.12 | 0.588 | 1.61 | 1.02 | 2.52 | 0.039 |
|  | Least poor | 1.01 | 0.59 | 1.74 | 0.957 | 0.96 | 0.46 | 1.99 | 0.913 | 1.38 | 0.79 | 2.42 | 0.258 |
| **Household members** | 1-4 members | 1.00 |  |  |  | 1.00 |  |  |  | 1.00 |  |  |  |
|  | 5-8 members | 1.03 | 0.75 | 1.42 | 0.862 | 1.38 | 0.90 | 2.12 | 0.140 | 0.75 | 0.53 | 1.04 | 0.088 |
|  | 9-12 members | 0.77 | 0.52 | 1.13 | 0.183 | 1.18 | 0.69 | 2.02 | 0.545 | 0.72 | 0.48 | 1.08 | 0.109 |
|  | 13 or more members | 1.09 | 0.69 | 1.74 | 0.703 | 1.07 | 0.55 | 2.08 | 0.837 | 1.25 | 0.77 | 2.03 | 0.362 |
| **Health care access (money)** | Big problem | 1.00 |  |  |  | 1.00 |  |  |  | 1.00 |  |  |  |
|  | Not a big problem | 0.91 | 0.68 | 1.22 | 0.514 | 0.75 | 0.50 | 1.13 | 0.169 | 1.86 | 1.37 | 2.53 | <0.001 |
| **Health care access (distance)** | Big problem | 1.00 |  |  |  | 1.00 |  |  |  | 1.00 |  |  |  |
|  | Not a big problem | 1.01 | 0.78 | 1.32 | 0.916 | 0.77 | 0.53 | 1.10 | 0.150 | 0.86 | 0.65 | 1.13 | 0.284 |
| **Symptoms** | Fever alone | 1.00 |  |  |  | 1.00 |  |  |  | 1.00 |  |  |  |
|  | Fever, cough | 0.73 | 0.53 | 1.00 | 0.049 | 0.60 | 0.38 | 0.95 | 0.030 | 2.39 | 1.71 | 3.33 | <0.001 |
|  | Fever, cough, rapid breaths | 0.70 | 0.49 | 0.99 | 0.044 | 0.57 | 0.35 | 0.95 | 0.032 | 3.59 | 2.44 | 5.26 | <0.001 |
| **Malaria transmission season** | Off-peak | 1.00 |  |  |  | 1.00 |  |  |  | 1.00 |  |  |  |
|  | Peak | 1.59 | 1.13 | 2.22 | 0.007 | 1.41 | 0.87 | 2.28 | 0.162 | 0.42 | 0.29 | 0.61 | <0.001 |
| **Health card** | No (never had or lost) | 1.00 |  |  |  | 1.00 |  |  |  | 1.00 |  |  |  |
|  | Yes (seen or reported) | 1.16 | 0.75 | 1.78 | 0.502 | 0.79 | 0.44 | 1.45 | 0.452 | 1.13 | 0.73 | 1.77 | 0.579 |

CI refers to confidence interval. AOR refers to adjusted odds ratio. AORs based on mixed-effects logistic regression models adjusted for data clustering and all listed covariates. Some covariates may have been included in model with collapsed response categories, and if so, this is noted in the above table.

**Burundi DHS 2010-2011**

|  |  | **Any anti-malarial use** | | | | **ACT Use** | | | | **Any antibiotic use** | | | |
| --- | --- | --- | --- | --- | --- | --- | --- | --- | --- | --- | --- | --- | --- |
|  |  | **AOR** | **95% CI** | | **pvalue** | **AOR** | **95% CI** | | **pvalue** | **AOR** | **95% CI** | | **pvalue** |
| **Diagnostic test use** | No | 1.00 |  |  |  | 1.00 |  |  |  | 1.00 |  |  |  |
|  | Yes | 3.71 | 2.63 | 5.25 | <0.001 | 2.78 | 1.81 | 4.27 | <0.001 | 0.53 | 0.40 | 0.72 | <0.001 |
| **Malaria endemicity** | Malaria-free | 0.25 | 0.07 | 0.85 | 0.026 | 1.00 |  |  |  | 0.98 | 0.47 | 2.06 | 0.954 |
|  | Low risk (*Pf*PR_2–10_ <5%) | 0.47 | 0.22 | 0.97 | 0.040 |  |  |  |  | 0.94 | 0.51 | 1.73 | 0.833 |
|  | Moderate risk (*Pf*PR_2–10_ 5-40%) | 1.00 |  |  |  |  |  |  |  | 1.00 |  |  |  |
|  | High risk (*Pf*PR_2–10_ >40%) | 0.39 | 0.03 | 4.41 | 0.446 | 6.80 | 2.31 | 20.02 | 0.001 | 3.80 | 0.54 | 26.96 | 0.182 |
| **Source (public/private)** | Public | 1.00 |  |  |  | 1.00 |  |  |  | 1.00 |  |  |  |
|  | Private | 0.70 | 0.33 | 1.49 | 0.356 | 0.72 | 0.26 | 2.04 | 0.540 | 1.49 | 0.79 | 2.80 | 0.220 |
| **Source (level)** | Hospital | 1.00 |  |  |  | 1.00 |  |  |  | 1.00 |  |  |  |
|  | Non-hospital formal medical | 0.86 | 0.51 | 1.45 | 0.566 | 1.39 | 0.68 | 2.84 | 0.367 | 1.05 | 0.68 | 1.63 | 0.826 |
|  | Community health worker | 1.45 | 0.42 | 4.98 | 0.551 | 1.13 | 0.20 | 6.27 | 0.886 | 0.65 | 0.21 | 1.97 | 0.444 |
|  | Pharmacy | 0.46 | 0.12 | 1.78 | 0.258 | 0.45 | 0.07 | 3.04 | 0.413 | 0.24 | 0.10 | 0.61 | 0.002 |
|  | Other | 0.80 | 0.25 | 2.51 | 0.698 | 1.68 | 0.42 | 6.79 | 0.464 | 0.45 | 0.17 | 1.19 | 0.106 |
| **Child's age (months)** | 0 - 5 | 0.30 | 0.09 | 0.98 | 0.046 | 0.31 | 0.06 | 1.65 | 0.171 | 1.16 | 0.64 | 2.09 | 0.621 |
|  | 6 - 11 | 1.00 |  |  |  | 1.00 |  |  |  | 1.00 |  |  |  |
|  | 12 - 23 | 2.16 | 1.19 | 3.92 | 0.011 | 3.18 | 1.39 | 7.25 | 0.006 | 0.90 | 0.59 | 1.38 | 0.625 |
|  | 24 - 35 | 6.97 | 3.81 | 12.74 | <0.001 | 10.75 | 4.71 | 24.54 | <0.001 | 0.60 | 0.38 | 0.95 | 0.028 |
|  | 36 - 47 | 3.93 | 2.08 | 7.42 | <0.001 | 5.63 | 2.38 | 13.33 | <0.001 | 0.52 | 0.33 | 0.84 | 0.007 |
|  | 48 - 59 | 6.01 | 3.03 | 11.90 | <0.001 | 5.90 | 2.36 | 14.78 | <0.001 | 0.52 | 0.30 | 0.88 | 0.016 |
| **Child's sex** | Male | 1.00 |  |  |  | 1.00 |  |  |  | 1.00 |  |  |  |
|  | Female | 0.82 | 0.59 | 1.13 | 0.219 | 0.87 | 0.58 | 1.30 | 0.506 | 1.03 | 0.79 | 1.34 | 0.845 |
| **Maternal age (years)** | 15 -24 | 1.00 |  |  |  | 1.00 |  |  |  | 1.00 |  |  |  |
|  | 25 - 29 | 0.85 | 0.54 | 1.35 | 0.498 | 1.45 | 0.81 | 2.57 | 0.210 | 1.07 | 0.73 | 1.57 | 0.719 |
|  | 30 - 34 | 0.77 | 0.45 | 1.29 | 0.318 | 1.01 | 0.52 | 1.95 | 0.982 | 1.41 | 0.90 | 2.20 | 0.137 |
|  | 35 - 39 | 0.62 | 0.34 | 1.11 | 0.107 | 0.71 | 0.33 | 1.50 | 0.370 | 1.09 | 0.68 | 1.76 | 0.723 |
|  | 40 - 49 | 0.87 | 0.45 | 1.67 | 0.667 | 1.11 | 0.48 | 2.54 | 0.811 | 0.98 | 0.56 | 1.73 | 0.951 |
| **Maternal education** | No education | 1.00 |  |  |  | 1.00 |  |  |  | 1.00 |  |  |  |
|  | Primary | 1.24 | 0.87 | 1.77 | 0.229 | 1.17 | 0.75 | 1.81 | 0.494 | 1.31 | 0.97 | 1.76 | 0.081 |
|  | Secondary or higher | 0.63 | 0.29 | 1.34 | 0.230 | 1.09 | 0.41 | 2.93 | 0.865 | 2.66 | 1.44 | 4.91 | 0.002 |
| **Residence** | Urban | 1.00 |  |  |  | 1.00 |  |  |  | 1.00 |  |  |  |
|  | Rural | 1.91 | 0.97 | 3.80 | 0.063 | 4.57 | 1.61 | 12.96 | 0.004 | 0.64 | 0.36 | 1.13 | 0.125 |
| **Household wealth** | Poorest | 1.00 |  |  |  | 1.00 |  |  |  | 1.00 |  |  |  |
|  | Second | 0.75 | 0.46 | 1.24 | 0.266 | 1.10 | 0.60 | 2.03 | 0.751 | 1.45 | 0.96 | 2.21 | 0.081 |
|  | Middle | 1.02 | 0.61 | 1.70 | 0.945 | 1.22 | 0.65 | 2.30 | 0.535 | 1.69 | 1.09 | 2.62 | 0.019 |
|  | Fourth | 1.05 | 0.63 | 1.77 | 0.848 | 1.44 | 0.75 | 2.75 | 0.276 | 1.27 | 0.81 | 1.97 | 0.297 |
|  | Least poor | 0.56 | 0.30 | 1.05 | 0.072 | 0.79 | 0.35 | 1.76 | 0.566 | 1.24 | 0.74 | 2.07 | 0.422 |
| **Household members** | 1-4 members | 1.00 |  |  |  | 1.00 |  |  |  | 1.00 |  |  |  |
|  | 5-8 members | 0.91 | 0.61 | 1.36 | 0.643 | 0.99 | 0.59 | 1.65 | 0.961 | 1.00 | 0.71 | 1.41 | 0.991 |
|  | 9-12 members | 1.23 | 0.65 | 2.34 | 0.520 | 1.50 | 0.66 | 3.40 | 0.329 | 0.77 | 0.44 | 1.33 | 0.343 |
|  | 13 or more members | 1.40 | 0.27 | 7.25 | 0.690 | 2.41 | 0.28 | 20.74 | 0.424 | 3.18 | 0.64 | 15.80 | 0.158 |
| **Health care access (money)** | Big problem | 1.00 |  |  |  | 1.00 |  |  |  | 1.00 |  |  |  |
|  | Not a big problem | 1.05 | 0.72 | 1.55 | 0.787 | 0.95 | 0.58 | 1.53 | 0.820 | 1.10 | 0.79 | 1.52 | 0.579 |
| **Health care access (distance)** | Big problem | 1.00 |  |  |  | 1.00 |  |  |  | 1.00 |  |  |  |
|  | Not a big problem | 1.05 | 0.74 | 1.50 | 0.766 | 1.45 | 0.93 | 2.26 | 0.098 | 0.91 | 0.68 | 1.22 | 0.535 |
| **Symptoms** | Fever alone | 1.00 |  |  |  | 1.00 |  |  |  | 1.00 |  |  |  |
|  | Fever, cough | 0.43 | 0.22 | 0.84 | 0.014 | 0.46 | 0.20 | 1.06 | 0.068 | 2.46 | 1.43 | 4.24 | 0.001 |
|  | Fever, cough, rapid breaths | 0.33 | 0.23 | 0.47 | <0.001 | 0.29 | 0.19 | 0.46 | <0.001 | 3.03 | 2.24 | 4.10 | <0.001 |
| **Malaria transmission season** | Off-peak | 1.00 |  |  |  | 1.00 |  |  |  | 1.00 |  |  |  |
|  | Peak | 1.45 | 0.96 | 2.21 | 0.081 | 1.77 | 0.99 | 3.16 | 0.054 | 0.91 | 0.62 | 1.34 | 0.648 |
| **Health card** | No (never had or lost) | 1.00 |  |  |  | 1.00 |  |  |  | 1.00 |  |  |  |
|  | Yes (seen or reported) | 1.31 | 0.88 | 1.94 | 0.179 | 1.49 | 0.90 | 2.44 | 0.118 | 1.02 | 0.73 | 1.42 | 0.916 |

CI refers to confidence interval. AOR refers to adjusted odds ratio. AORs based on mixed-effects logistic regression models adjusted for data clustering and all listed covariates. Some covariates may have been included in model with collapsed response categories, and if so, this is noted in the above table.

**Cote d’Ivoire DHS 2011-2012**

|  |  | **Any anti-malarial use** | | | | **ACT Use** | | | | **Any antibiotic use** | | | |
| --- | --- | --- | --- | --- | --- | --- | --- | --- | --- | --- | --- | --- | --- |
|  |  | **AOR** | **95% CI** | | **pvalue** | **AOR** | **95% CI** | | **pvalue** | **AOR** | **95% CI** | | **pvalue** |
| **Diagnostic test use** | No | 1.00 |  |  |  | 1.00 |  |  |  | 1.00 |  |  |  |
|  | Yes | 1.89 | 1.14 | 3.13 | 0.013 | 16.83 | 1.03 | 276.13 | 0.048 | 1.08 | 0.68 | 1.74 | 0.737 |
| **Malaria endemicity** | Moderate or high risk (*Pf*PR_2–10_ >5%) | 1.00 |  |  |  |  |  |  |  |  |  |  |  |
| **Source (public/private)** | Public | 1.00 |  |  |  | 1.00 |  |  |  | 1.00 |  |  |  |
|  | Private | 2.41 | 1.04 | 5.63 | 0.041 | 1.90 | 0.16 | 22.54 | 0.609 | 0.92 | 0.41 | 2.09 | 0.843 |
| **Source (level)** | Hospital | 1.00 |  |  |  | 1.00 |  |  |  | 1.00 |  |  |  |
|  | Non-hospital formal medical | 0.68 | 0.38 | 1.21 | 0.191 | 0.35 | 0.03 | 4.08 | 0.406 | 0.80 | 0.46 | 1.39 | 0.425 |
|  | Community health worker | 1.72 | 0.28 | 10.62 | 0.560 |  |  |  |  | 0.49 | 0.08 | 3.11 | 0.446 |
|  | Pharmacy | 0.19 | 0.07 | 0.49 | 0.001 |  |  |  |  | 0.68 | 0.28 | 1.65 | 0.397 |
|  | Other | 0.07 | 0.03 | 0.18 | <0.001 | 0.07 | 0.01 | 1.01 | 0.051 | 0.14 | 0.06 | 0.33 | <0.001 |
| **Child's age (months)** | 0 - 5 | 0.36 | 0.13 | 1.04 | 0.060 |  |  |  |  | 4.14 | 1.90 | 9.00 | <0.001 |
|  | 6 - 11 | 1.00 |  |  |  | 1.00 |  |  |  | 1.00 |  |  |  |
|  | 12 - 23 | 0.64 | 0.34 | 1.21 | 0.172 | 6.34 | 0.47 | 85.44 | 0.164 | 1.14 | 0.68 | 1.91 | 0.624 |
|  | 24 - 35 | 1.37 | 0.73 | 2.55 | 0.323 |  |  |  |  | 1.07 | 0.62 | 1.84 | 0.800 |
|  | 36 - 47 | 1.32 | 0.69 | 2.52 | 0.402 |  |  |  |  | 0.57 | 0.32 | 1.03 | 0.063 |
|  | 48 - 59 | 1.45 | 0.69 | 3.05 | 0.321 |  |  |  |  | 0.64 | 0.33 | 1.25 | 0.194 |
| **Child's sex** | Male | 1.00 |  |  |  | 1.00 |  |  |  | 1.00 |  |  |  |
|  | Female | 0.77 | 0.52 | 1.15 | 0.196 | 1.08 | 0.29 | 3.99 | 0.904 | 1.04 | 0.74 | 1.46 | 0.820 |
| **Maternal age (years)** | 15 -24 | 1.00 |  |  |  | 1.00 |  |  |  | 1.00 |  |  |  |
|  | 25 - 29 | 1.00 | 0.60 | 1.66 | 0.995 | 5.56 | 0.69 | 44.66 | 0.107 | 1.06 | 0.68 | 1.64 | 0.807 |
|  | 30 - 34 | 0.61 | 0.33 | 1.16 | 0.132 | 0.00 | 0.00 | . | 0.990 | 1.41 | 0.85 | 2.35 | 0.187 |
|  | 35 - 39 | 1.03 | 0.56 | 1.90 | 0.914 | 6.49 | 0.79 | 53.13 | 0.081 | 1.36 | 0.80 | 2.34 | 0.257 |
|  | 40 - 49 | 1.24 | 0.54 | 2.81 | 0.615 | 9.69 | 0.57 | 164.60 | 0.116 | 0.62 | 0.28 | 1.38 | 0.242 |
| **Maternal education** | No education | 1.00 |  |  |  | 1.00 |  |  |  | 1.00 |  |  |  |
|  | Primary | 1.87 | 1.18 | 2.97 | 0.008 | 0.30 | 0.05 | 1.94 | 0.208 | 0.84 | 0.55 | 1.28 | 0.410 |
|  | Secondary or higher | 1.12 | 0.59 | 2.15 | 0.724 | 0.72 | 0.08 | 6.38 | 0.769 | 0.87 | 0.49 | 1.54 | 0.635 |
| **Residence** | Urban | 1.00 |  |  |  | 1.00 |  |  |  | 1.00 |  |  |  |
|  | Rural | 1.16 | 0.59 | 2.28 | 0.666 | 3.83 | 0.34 | 43.21 | 0.277 | 0.86 | 0.47 | 1.55 | 0.611 |
| **Household wealth** | Poorest | 1.00 |  |  |  | 1.00 |  |  |  | 1.00 |  |  |  |
|  | Second | 1.35 | 0.67 | 2.71 | 0.396 | 1.79 | 0.14 | 23.41 | 0.657 | 1.00 | 0.55 | 1.82 | 0.990 |
|  | Middle | 1.01 | 0.49 | 2.07 | 0.987 | 1.63 | 0.12 | 21.48 | 0.708 | 1.12 | 0.62 | 2.04 | 0.709 |
|  | Fourth | 1.35 | 0.60 | 3.04 | 0.474 | 2.44 | 0.13 | 46.30 | 0.551 | 1.11 | 0.55 | 2.22 | 0.768 |
|  | Least poor | 1.40 | 0.53 | 3.67 | 0.494 | 6.98 | 0.23 | 207.59 | 0.262 | 0.93 | 0.40 | 2.14 | 0.857 |
| **Household members** | 1-4 members | 1.00 |  |  |  | 1.00 |  |  |  | 1.00 |  |  |  |
|  | 5-8 members | 1.10 | 0.65 | 1.87 | 0.714 | 0.43 | 0.08 | 2.32 | 0.326 | 1.18 | 0.75 | 1.88 | 0.474 |
|  | 9-12 members | 1.13 | 0.59 | 2.16 | 0.705 | 0.39 | 0.05 | 3.20 | 0.378 | 0.98 | 0.56 | 1.74 | 0.955 |
|  | 13 or more members | 0.87 | 0.42 | 1.81 | 0.704 | 0.18 | 0.01 | 2.74 | 0.219 | 1.10 | 0.59 | 2.04 | 0.769 |
| **Health care access (money)** | Big problem | 1.00 |  |  |  | 1.00 |  |  |  | 1.00 |  |  |  |
|  | Not a big problem | 0.75 | 0.48 | 1.19 | 0.226 | 0.82 | 0.17 | 3.97 | 0.803 | 0.92 | 0.62 | 1.37 | 0.686 |
| **Health care access (distance)** | Big problem | 1.00 |  |  |  | 1.00 |  |  |  | 1.00 |  |  |  |
|  | Not a big problem | 1.25 | 0.80 | 1.95 | 0.323 | 2.49 | 0.48 | 12.83 | 0.275 | 0.98 | 0.67 | 1.44 | 0.938 |
| **Symptoms** | Fever alone | 1.00 |  |  |  | 1.00 |  |  |  | 1.00 |  |  |  |
|  | Fever, cough | 0.91 | 0.56 | 1.46 | 0.688 | 1.15 | 0.23 | 5.85 | 0.868 | 1.58 | 1.04 | 2.39 | 0.032 |
|  | Fever, cough, rapid breaths | 0.74 | 0.46 | 1.22 | 0.239 | 0.64 | 0.11 | 3.82 | 0.624 | 1.50 | 0.99 | 2.26 | 0.056 |
| **Malaria transmission season** | Off-peak | 1.00 |  |  |  | 1.00 |  |  |  | 1.00 |  |  |  |
|  | Peak | 1.92 | 1.20 | 3.08 | 0.006 | 1.54 | 0.29 | 8.13 | 0.613 | 1.06 | 0.67 | 1.65 | 0.813 |
| **Health card** | No (never had or lost) | 1.00 |  |  |  | 1.00 |  |  |  | 1.00 |  |  |  |
|  | Yes (seen or reported) | 0.72 | 0.27 | 1.90 | 0.505 | 0.63 | 0.02 | 16.84 | 0.786 | 0.96 | 0.45 | 2.06 | 0.912 |

CI refers to confidence interval. AOR refers to adjusted odds ratio. AORs based on mixed-effects logistic regression models adjusted for data clustering and all listed covariates. Some covariates may have been included in model with collapsed response categories, and if so, this is noted in the above table.

**Gabon DHS 2012**

|  |  | **Any anti-malarial use** | | | | **ACT Use** | | | | **Any antibiotic use** | | | |
| --- | --- | --- | --- | --- | --- | --- | --- | --- | --- | --- | --- | --- | --- |
|  |  | **AOR** | **95% CI** | | **pvalue** | **AOR** | **95% CI** | | **pvalue** | **AOR** | **95% CI** | | **pvalue** |
| **Diagnostic test use** | No | 1.00 |  |  |  | 1.00 |  |  |  | 1.00 |  |  |  |
|  | Yes | 2.00 | 1.16 | 3.44 | 0.013 | 2.45 | 1.13 | 5.33 | 0.024 | 0.84 | 0.52 | 1.35 | 0.467 |
| **Malaria endemicity** | Moderate risk (*Pf*PR_2–10_ 5-40%) | 1.00 |  |  |  | 1.00 |  |  |  | 1.00 |  |  |  |
|  | High risk (*Pf*PR_2–10_ >40%) | 0.73 | 0.31 | 1.71 | 0.473 | 2.07 | 0.49 | 8.82 | 0.323 | 0.76 | 0.38 | 1.51 | 0.434 |
| **Source (public/private)** | Public | 1.00 |  |  |  | 1.00 |  |  |  | 1.00 |  |  |  |
|  | Private | 1.48 | 0.60 | 3.68 | 0.395 | 0.47 | 0.11 | 2.00 | 0.304 | 0.81 | 0.37 | 1.80 | 0.610 |
| **Source (level)** | Hospital | 1.00 |  |  |  | 1.00 |  |  |  | 1.00 |  |  |  |
|  | Non-hospital formal medical | 0.47 | 0.26 | 0.86 | 0.014 | 0.96 | 0.41 | 2.25 | 0.918 | 0.70 | 0.44 | 1.12 | 0.139 |
|  | Community health worker | 0.00 | 0.00 | . | 0.983 |  |  |  |  | 0.00 | 0.00 | . | 0.980 |
|  | Pharmacy | 0.52 | 0.19 | 1.40 | 0.198 |  |  |  |  | 0.86 | 0.36 | 2.04 | 0.727 |
|  | Other | 0.19 | 0.05 | 0.76 | 0.019 | 1.11 | 0.23 | 5.33 | 0.892 | 0.34 | 0.11 | 1.04 | 0.060 |
| **Child's age (months)** | 0 - 5 | 0.39 | 0.13 | 1.24 | 0.110 | 1.51 | 0.21 | 10.68 | 0.682 | 0.92 | 0.40 | 2.13 | 0.853 |
|  | 6 - 11 | 1.00 |  |  |  | 1.00 |  |  |  | 1.00 |  |  |  |
|  | 12 - 23 | 1.25 | 0.62 | 2.49 | 0.535 | 3.21 | 0.88 | 11.79 | 0.079 | 0.67 | 0.38 | 1.18 | 0.166 |
|  | 24 - 35 | 1.16 | 0.56 | 2.38 | 0.691 | 3.72 | 0.98 | 14.10 | 0.054 | 0.81 | 0.45 | 1.46 | 0.490 |
|  | 36 - 47 | 1.71 | 0.83 | 3.52 | 0.149 | 2.86 | 0.76 | 10.67 | 0.119 | 0.70 | 0.38 | 1.27 | 0.238 |
|  | 48 - 59 | 1.69 | 0.76 | 3.74 | 0.195 | 2.77 | 0.64 | 11.99 | 0.173 | 1.13 | 0.58 | 2.21 | 0.721 |
| **Child's sex** | Male | 1.00 |  |  |  | 1.00 |  |  |  | 1.00 |  |  |  |
|  | Female | 1.09 | 0.70 | 1.69 | 0.699 | 1.42 | 0.71 | 2.81 | 0.321 | 1.09 | 0.76 | 1.56 | 0.649 |
| **Maternal age (years)** | 15 -24 | 1.00 |  |  |  | 1.00 |  |  |  | 1.00 |  |  |  |
|  | 25 - 29 | 0.87 | 0.48 | 1.60 | 0.661 | 1.56 | 0.67 | 3.64 | 0.303 | 1.69 | 1.03 | 2.78 | 0.037 |
|  | 30 - 34 | 1.33 | 0.71 | 2.48 | 0.368 | 1.02 | 0.38 | 2.74 | 0.961 | 1.34 | 0.78 | 2.29 | 0.283 |
|  | 35 - 39 | 1.07 | 0.54 | 2.11 | 0.856 |  |  |  |  | 1.15 | 0.66 | 2.01 | 0.616 |
|  | 40 - 49 | 0.65 | 0.26 | 1.61 | 0.348 | 0.48 | 0.16 | 1.43 | 0.189 | 1.34 | 0.64 | 2.77 | 0.437 |
| **Maternal education** | No education | 1.00 |  |  |  | 1.00 |  |  |  | 1.00 |  |  |  |
|  | Primary | 1.83 | 0.57 | 5.87 | 0.311 | 0.92 | 0.18 | 4.57 | 0.917 | 0.66 | 0.27 | 1.61 | 0.367 |
|  | Secondary or higher | 2.22 | 0.71 | 6.91 | 0.169 | 1.13 | 0.25 | 5.12 | 0.870 | 0.81 | 0.34 | 1.93 | 0.631 |
| **Residence** | Urban | 1.00 |  |  |  | 1.00 |  |  |  | 1.00 |  |  |  |
|  | Rural | 1.28 | 0.68 | 2.41 | 0.445 | 1.68 | 0.68 | 4.11 | 0.258 | 0.90 | 0.55 | 1.48 | 0.686 |
| **Household wealth** | Poorest | 1.00 |  |  |  | 1.00 |  |  |  | 1.00 |  |  |  |
|  | Second | 0.82 | 0.42 | 1.61 | 0.572 | 2.07 | 0.73 | 5.87 | 0.170 | 0.96 | 0.56 | 1.65 | 0.889 |
|  | Middle | 0.68 | 0.31 | 1.46 | 0.321 | 1.29 | 0.39 | 4.25 | 0.675 | 1.26 | 0.68 | 2.32 | 0.467 |
|  | Fourth | 2.06 | 0.94 | 4.49 | 0.069 | 5.01 | 1.58 | 15.92 | 0.006 | 1.23 | 0.64 | 2.39 | 0.532 |
|  | Least poor | 0.65 | 0.25 | 1.73 | 0.392 | 3.28 | 0.85 | 12.72 | 0.085 | 0.95 | 0.43 | 2.10 | 0.890 |
| **Household members** | 1-4 members | 1.00 |  |  |  | 1.00 |  |  |  | 1.00 |  |  |  |
|  | 5-8 members | 0.92 | 0.52 | 1.62 | 0.772 | 0.82 | 0.36 | 1.86 | 0.640 | 1.13 | 0.70 | 1.83 | 0.615 |
|  | 9-12 members | 0.75 | 0.37 | 1.51 | 0.416 | 0.28 | 0.09 | 0.91 | 0.034 | 1.12 | 0.63 | 1.98 | 0.699 |
|  | 13 or more members | 0.64 | 0.27 | 1.50 | 0.301 |  |  |  |  | 0.98 | 0.50 | 1.92 | 0.943 |
| **Health care access (money)** | Big problem | 1.00 |  |  |  | 1.00 |  |  |  | 1.00 |  |  |  |
|  | Not a big problem | 0.85 | 0.47 | 1.52 | 0.573 | 0.95 | 0.38 | 2.41 | 0.921 | 1.35 | 0.84 | 2.18 | 0.219 |
| **Health care access (distance)** | Big problem | 1.00 |  |  |  | 1.00 |  |  |  | 1.00 |  |  |  |
|  | Not a big problem | 0.95 | 0.58 | 1.56 | 0.842 | 0.66 | 0.30 | 1.43 | 0.292 | 0.93 | 0.62 | 1.39 | 0.712 |
| **Symptoms** | Fever alone | 1.00 |  |  |  | 1.00 |  |  |  | 1.00 |  |  |  |
|  | Fever, cough | 0.51 | 0.30 | 0.88 | 0.016 | 0.72 | 0.33 | 1.57 | 0.405 | 2.03 | 1.30 | 3.19 | 0.002 |
|  | Fever, cough, rapid breaths | 0.42 | 0.25 | 0.73 | 0.002 | 0.41 | 0.17 | 1.02 | 0.054 | 1.92 | 1.22 | 3.01 | 0.005 |
| **Malaria transmission season** | Off-peak | 1.00 |  |  |  | 1.00 |  |  |  | 1.00 |  |  |  |
|  | Peak | 0.46 | 0.03 | 6.20 | 0.556 | 0.04 | 0.00 | 0.77 | 0.033 | 0.72 | 0.08 | 6.79 | 0.774 |
| **Health card** | No (never had or lost) | 1.00 |  |  |  | 1.00 |  |  |  | 1.00 |  |  |  |
|  | Yes (seen or reported) | 1.21 | 0.55 | 2.63 | 0.636 | 2.00 | 0.40 | 9.99 | 0.398 | 1.52 | 0.80 | 2.88 | 0.197 |

CI refers to confidence interval. AOR refers to adjusted odds ratio. AORs based on mixed-effects logistic regression models adjusted for data clustering and all listed covariates. Some covariates may have been included in model with collapsed response categories, and if so, this is noted in the above table.

**Guinea DHS 2012**

|  |  | **Any anti-malarial use** | | | | **ACT Use** | | | | **Any antibiotic use** | | | |
| --- | --- | --- | --- | --- | --- | --- | --- | --- | --- | --- | --- | --- | --- |
|  |  | **AOR** | **95% CI** | | **pvalue** | **AOR** | **95% CI** | | **pvalue** | **AOR** | **95% CI** | | **pvalue** |
| **Diagnostic test use** | No | 1.00 |  |  |  | 1.00 |  |  |  | 1.00 |  |  |  |
|  | Yes | 1.28 | 0.78 | 2.11 | 0.330 | 2.42 | 0.43 | 13.68 | 0.319 | 1.05 | 0.63 | 1.75 | 0.862 |
| **Malaria endemicity** | Moderate risk (*Pf*PR_2–10_ 5-40%) | 1.00 |  |  |  | 1.00 |  |  |  | 1.00 |  |  |  |
|  | High risk (*Pf*PR_2–10_ >40%) | 1.76 | 1.13 | 2.74 | 0.012 | 10.24 | 1.35 | 77.89 | 0.025 | 0.42 | 0.26 | 0.68 | <0.001 |
| **Source (public/private)** | Public | 1.00 |  |  |  | 1.00 |  |  |  | 1.00 |  |  |  |
|  | Private | 0.83 | 0.38 | 1.84 | 0.652 | 0.44 | 0.03 | 6.06 | 0.540 | 2.28 | 1.00 | 5.20 | 0.051 |
| **Source (level)** | Hospital | 1.00 |  |  |  | 1.00 |  |  |  | 1.00 |  |  |  |
|  | Non-hospital formal medical | 1.17 | 0.60 | 2.26 | 0.643 | 0.64 | 0.08 | 5.26 | 0.679 | 1.40 | 0.71 | 2.76 | 0.327 |
|  | Pharmacy | 0.53 | 0.20 | 1.40 | 0.201 | 0.00 | 0.00 | . | 0.991 | 0.94 | 0.35 | 2.56 | 0.908 |
|  | Other | 0.32 | 0.13 | 0.79 | 0.013 | 0.13 | 0.00 | 6.11 | 0.299 | 0.10 | 0.04 | 0.25 | <0.001 |
| **Child's age (months)** | 0 - 5 | 0.66 | 0.30 | 1.42 | 0.286 | 1.00 |  |  |  | 1.69 | 0.76 | 3.75 | 0.197 |
|  | 6 - 11 | 1.00 |  |  |  | 1.14 | 0.10 | 12.61 | 0.913 | 1.00 |  |  |  |
|  | 12 - 23 | 1.21 | 0.70 | 2.09 | 0.495 | 0.06 | 0.00 | 0.99 | 0.049 | 0.67 | 0.37 | 1.20 | 0.174 |
|  | 24 - 35 | 1.02 | 0.58 | 1.81 | 0.935 | 0.17 | 0.01 | 2.22 | 0.176 | 0.80 | 0.44 | 1.46 | 0.471 |
|  | 36 - 47 | 1.22 | 0.68 | 2.17 | 0.506 | 0.14 | 0.01 | 2.48 | 0.181 | 0.67 | 0.36 | 1.23 | 0.191 |
|  | 48 - 59 | 1.39 | 0.75 | 2.61 | 0.299 | 0.10 | 0.01 | 1.71 | 0.110 | 0.95 | 0.49 | 1.85 | 0.885 |
| **Child's sex** | Male | 1.00 |  |  |  | 1.00 |  |  |  | 1.00 |  |  |  |
|  | Female | 0.85 | 0.63 | 1.17 | 0.327 | 0.42 | 0.10 | 1.82 | 0.246 | 1.05 | 0.75 | 1.46 | 0.778 |
| **Maternal age (years)** | 15 -24 | 1.00 |  |  |  | 1.00 |  |  |  | 1.00 |  |  |  |
|  | 25 - 29 | 1.37 | 0.91 | 2.08 | 0.135 | 2.46 | 0.39 | 15.73 | 0.341 | 0.85 | 0.55 | 1.31 | 0.455 |
|  | 30 - 34 | 1.27 | 0.79 | 2.04 | 0.320 | 1.22 | 0.17 | 8.84 | 0.843 | 0.93 | 0.56 | 1.54 | 0.782 |
|  | 35 - 39 | 0.71 | 0.43 | 1.17 | 0.177 | 2.92 | 0.30 | 28.29 | 0.355 | 0.64 | 0.38 | 1.09 | 0.103 |
|  | 40 - 49 | 0.52 | 0.27 | 0.98 | 0.045 | 0.54 | 0.03 | 9.32 | 0.675 | 1.34 | 0.68 | 2.65 | 0.396 |
| **Maternal education** | No education | 1.00 |  |  |  | 1.00 |  |  |  | 1.00 |  |  |  |
|  | Primary | 1.04 | 0.66 | 1.64 | 0.873 | 0.00 | 0.00 | . | 0.986 | 1.47 | 0.90 | 2.39 | 0.123 |
|  | Secondary or higher | 0.82 | 0.48 | 1.38 | 0.456 | 0.46 | 0.08 | 2.76 | 0.394 | 1.30 | 0.76 | 2.25 | 0.340 |
| **Residence** | Urban | 1.00 |  |  |  | 1.00 |  |  |  | 1.00 |  |  |  |
|  | Rural | 0.95 | 0.49 | 1.84 | 0.883 | 0.08 | 0.01 | 1.29 | 0.075 | 2.12 | 1.07 | 4.23 | 0.032 |
| **Household wealth** | Poorest | 1.00 |  |  |  | 1.00 |  |  |  | 1.00 |  |  |  |
|  | Second | 0.82 | 0.48 | 1.38 | 0.452 | 0.00 | 0.00 | . | 0.985 | 0.89 | 0.51 | 1.54 | 0.669 |
|  | Middle | 1.04 | 0.62 | 1.74 | 0.889 | 0.32 | 0.04 | 2.78 | 0.300 | 0.63 | 0.36 | 1.09 | 0.101 |
|  | Fourth | 1.22 | 0.65 | 2.30 | 0.530 | 0.44 | 0.04 | 5.37 | 0.520 | 1.25 | 0.65 | 2.41 | 0.505 |
|  | Least poor | 0.66 | 0.26 | 1.67 | 0.382 | 0.15 | 0.01 | 4.21 | 0.262 | 1.96 | 0.76 | 5.07 | 0.164 |
| **Household members** | 1-4 members | 1.00 |  |  |  |  |  |  |  | 1.00 |  |  |  |
|  | 5-8 members | 1.34 | 0.83 | 2.15 | 0.230 |  |  |  |  | 1.66 | 1.00 | 2.75 | 0.050 |
|  | 9-12 members | 1.45 | 0.85 | 2.45 | 0.171 |  |  |  |  | 1.58 | 0.90 | 2.75 | 0.110 |
|  | 13 or more members | 1.04 | 0.57 | 1.90 | 0.889 |  |  |  |  | 2.23 | 1.17 | 4.26 | 0.015 |
| **Health care access (money)** | Big problem |  |  |  |  |  |  |  |  |  |  |  |  |
|  | Not a big problem |  |  |  |  |  |  |  |  |  |  |  |  |
| **Health care access (distance)** | Big problem |  |  |  |  |  |  |  |  |  |  |  |  |
|  | Not a big problem |  |  |  |  |  |  |  |  |  |  |  |  |
| **Symptoms** | Fever alone | 1.00 |  |  |  | 1.00 |  |  |  | 1.00 |  |  |  |
|  | Fever, cough | 0.84 | 0.53 | 1.32 | 0.442 | 4.37 | 0.82 | 23.19 | 0.084 | 2.02 | 1.24 | 3.29 | 0.004 |
|  | Fever, cough, rapid breaths | 0.87 | 0.60 | 1.27 | 0.474 | 1.58 | 0.30 | 8.38 | 0.588 | 2.14 | 1.44 | 3.20 | <0.001 |
| **Malaria transmission season** | Off-peak | 1.00 |  |  |  | 1.00 |  |  |  | 1.00 |  |  |  |
|  | Peak | 0.00 | 0.00 | . | 0.974 | 0.00 | 0.00 | . | 0.992 | 0.00 | 0.00 | . | 0.944 |
| **Health card** | No (never had or lost) | 1.00 |  |  |  | 1.00 |  |  |  | 1.00 |  |  |  |
|  | Yes (seen or reported) | 0.87 | 0.55 | 1.38 | 0.561 | 4.89 | 0.29 | 82.19 | 0.270 | 1.41 | 0.86 | 2.32 | 0.178 |

CI refers to confidence interval. AOR refers to adjusted odds ratio. AORs based on mixed-effects logistic regression models adjusted for data clustering and all listed covariates. Some covariates may have been included in model with collapsed response categories, and if so, this is noted in the above table. Blank cells in the results table indicate the covariate could not be included in the final model.

**Malawi DHS 2010**

|  |  | **Any anti-malarial use** | | | | **ACT Use** | | | | **Any antibiotic use** | | | |
| --- | --- | --- | --- | --- | --- | --- | --- | --- | --- | --- | --- | --- | --- |
|  |  | **AOR** | **95% CI** | | **pvalue** | **AOR** | **95% CI** | | **pvalue** | **AOR** | **95% CI** | | **pvalue** |
| **Diagnostic test use** | No | 1.00 |  |  |  | 1.00 |  |  |  | 1.00 |  |  |  |
|  | Yes | 1.34 | 1.11 | 1.61 | 0.002 | 1.12 | 0.94 | 1.34 | 0.206 | 1.00 | 0.82 | 1.22 | 1.000 |
| **Malaria endemicity** | Moderate risk (*Pf*PR_2–10_ 5-40%) | 1.00 |  |  |  | 1.00 |  |  |  | 1.00 |  |  |  |
|  | High risk (*Pf*PR_2–10_ >40%) | 1.15 | 0.96 | 1.37 | 0.123 | 1.41 | 1.18 | 1.68 | <0.001 | 0.94 | 0.78 | 1.14 | 0.547 |
| **Source (public/private)** | Public | 1.00 |  |  |  | 1.00 |  |  |  | 1.00 |  |  |  |
|  | Private | 0.84 | 0.68 | 1.04 | 0.118 | 0.50 | 0.40 | 0.62 | <0.001 | 1.91 | 1.53 | 2.39 | <0.001 |
| **Source (level)** | Hospital | 1.00 |  |  |  | 1.00 |  |  |  | 1.00 |  |  |  |
|  | Non-hospital formal medical | 0.94 | 0.78 | 1.14 | 0.543 | 1.03 | 0.85 | 1.23 | 0.789 | 0.96 | 0.79 | 1.17 | 0.686 |
|  | Other or pharmacy | 0.07 | 0.05 | 0.10 | <0.001 | 0.10 | 0.07 | 0.15 | <0.001 | 0.44 | 0.32 | 0.60 | <0.001 |
| **Child's age (months)** | 0 - 5 | 0.39 | 0.27 | 0.55 | <0.001 | 0.33 | 0.22 | 0.48 | <0.001 | 1.46 | 1.05 | 2.04 | 0.026 |
|  | 6 - 11 | 1.00 |  |  |  | 1.00 |  |  |  | 1.00 |  |  |  |
|  | 12 - 23 | 1.82 | 1.45 | 2.28 | <0.001 | 1.77 | 1.41 | 2.23 | <0.001 | 0.65 | 0.51 | 0.83 | 0.001 |
|  | 24 - 35 | 2.15 | 1.70 | 2.72 | <0.001 | 1.97 | 1.56 | 2.50 | <0.001 | 0.73 | 0.57 | 0.93 | 0.011 |
|  | 36 - 47 | 1.95 | 1.52 | 2.51 | <0.001 | 1.88 | 1.46 | 2.42 | <0.001 | 0.69 | 0.53 | 0.90 | 0.006 |
|  | 48 - 59 | 1.93 | 1.49 | 2.51 | <0.001 | 1.96 | 1.51 | 2.55 | <0.001 | 0.66 | 0.50 | 0.87 | 0.003 |
| **Child's sex** | Male | 1.00 |  |  |  | 1.00 |  |  |  | 1.00 |  |  |  |
|  | Female | 0.90 | 0.79 | 1.04 | 0.165 | 0.98 | 0.85 | 1.13 | 0.770 | 1.01 | 0.87 | 1.17 | 0.934 |
| **Maternal age (years)** | 15 -24 | 1.00 |  |  |  | 1.00 |  |  |  | 1.00 |  |  |  |
|  | 25 - 29 | 1.02 | 0.85 | 1.24 | 0.812 | 0.98 | 0.81 | 1.18 | 0.834 | 1.16 | 0.95 | 1.42 | 0.137 |
|  | 30 - 34 | 1.07 | 0.85 | 1.34 | 0.571 | 0.91 | 0.73 | 1.13 | 0.396 | 1.03 | 0.81 | 1.31 | 0.799 |
|  | 35 - 39 | 0.97 | 0.75 | 1.26 | 0.843 | 0.92 | 0.71 | 1.19 | 0.521 | 1.15 | 0.88 | 1.51 | 0.313 |
|  | 40 - 49 | 1.09 | 0.79 | 1.51 | 0.605 | 1.07 | 0.77 | 1.48 | 0.675 | 0.84 | 0.59 | 1.20 | 0.334 |
| **Maternal education** | No education | 1.00 |  |  |  | 1.00 |  |  |  | 1.00 |  |  |  |
|  | Primary | 1.26 | 1.01 | 1.57 | 0.039 | 1.13 | 0.91 | 1.40 | 0.282 | 0.96 | 0.76 | 1.22 | 0.749 |
|  | Secondary or higher | 1.24 | 0.92 | 1.68 | 0.158 | 1.10 | 0.82 | 1.48 | 0.532 | 1.32 | 0.96 | 1.81 | 0.088 |
| **Residence** | Urban | 1.00 |  |  |  | 1.00 |  |  |  | 1.00 |  |  |  |
|  | Rural | 1.52 | 1.10 | 2.09 | 0.011 | 1.23 | 0.89 | 1.69 | 0.210 | 0.74 | 0.53 | 1.04 | 0.088 |
| **Household wealth** | Poorest | 1.00 |  |  |  | 1.00 |  |  |  | 1.00 |  |  |  |
|  | Second | 1.05 | 0.85 | 1.30 | 0.674 | 1.09 | 0.88 | 1.34 | 0.437 | 0.96 | 0.76 | 1.21 | 0.735 |
|  | Middle | 1.00 | 0.81 | 1.24 | 0.972 | 1.05 | 0.85 | 1.29 | 0.666 | 1.29 | 1.03 | 1.62 | 0.026 |
|  | Fourth | 1.00 | 0.80 | 1.26 | 0.986 | 0.94 | 0.75 | 1.18 | 0.591 | 1.33 | 1.04 | 1.69 | 0.023 |
|  | Least poor | 1.38 | 1.02 | 1.87 | 0.037 | 1.09 | 0.81 | 1.46 | 0.586 | 0.97 | 0.71 | 1.34 | 0.867 |
| **Household members** | 1-4 members | 1.00 |  |  |  | 1.00 |  |  |  | 1.00 |  |  |  |
|  | 5-8 members | 1.02 | 0.86 | 1.22 | 0.816 | 1.08 | 0.91 | 1.29 | 0.381 | 0.98 | 0.81 | 1.18 | 0.828 |
|  | 9-12 members | 1.14 | 0.87 | 1.49 | 0.352 | 1.14 | 0.87 | 1.49 | 0.346 | 1.05 | 0.79 | 1.39 | 0.751 |
|  | 13 or more members | 1.76 | 0.91 | 3.40 | 0.090 | 1.64 | 0.88 | 3.03 | 0.116 | 1.10 | 0.59 | 2.06 | 0.771 |
| **Health care access (money)** | Big problem | 1.00 |  |  |  | 1.00 |  |  |  | 1.00 |  |  |  |
|  | Not a big problem | 1.05 | 0.89 | 1.23 | 0.589 | 0.95 | 0.81 | 1.12 | 0.539 | 1.04 | 0.88 | 1.24 | 0.637 |
| **Health care access (distance)** | Big problem | 1.00 |  |  |  | 1.00 |  |  |  | 1.00 |  |  |  |
|  | Not a big problem | 0.97 | 0.82 | 1.15 | 0.723 | 0.97 | 0.82 | 1.15 | 0.735 | 1.01 | 0.85 | 1.21 | 0.902 |
| **Symptoms** | Fever alone | 1.00 |  |  |  | 1.00 |  |  |  | 1.00 |  |  |  |
|  | Fever, cough | 0.66 | 0.55 | 0.80 | <0.001 | 0.75 | 0.62 | 0.90 | 0.002 | 2.64 | 2.17 | 3.22 | <0.001 |
|  | Fever, cough, rapid breaths | 0.51 | 0.43 | 0.60 | <0.001 | 0.48 | 0.41 | 0.56 | <0.001 | 2.94 | 2.46 | 3.52 | <0.001 |
| **Malaria transmission season** | Off-peak | 1.00 |  |  |  | 1.00 |  |  |  | 1.00 |  |  |  |
|  | Peak | 1.37 | 0.54 | 3.45 | 0.507 | 1.04 | 0.42 | 2.55 | 0.935 | 0.71 | 0.26 | 1.97 | 0.516 |
| **Health card** | No (never had or lost) | 1.00 |  |  |  | 1.00 |  |  |  | 1.00 |  |  |  |
|  | Yes (seen or reported) | 1.06 | 0.83 | 1.35 | 0.645 | 1.10 | 0.86 | 1.39 | 0.456 | 1.14 | 0.87 | 1.49 | 0.337 |

CI refers to confidence interval. AOR refers to adjusted odds ratio. AORs based on mixed-effects logistic regression models adjusted for data clustering and all listed covariates. Some covariates may have been included in model with collapsed response categories, and if so, this is noted in the above table.

**Mozambique DHS 2011**

|  |  | **Any anti-malarial use** | | | | **ACT Use** | | | | **Any antibiotic use** | | | |
| --- | --- | --- | --- | --- | --- | --- | --- | --- | --- | --- | --- | --- | --- |
|  |  | **AOR** | **95% CI** | | **pvalue** | **AOR** | **95% CI** | | **pvalue** | **AOR** | **95% CI** | | **pvalue** |
| **Diagnostic test use** | No | 1.00 |  |  |  | 1.00 |  |  |  | 1.00 |  |  |  |
|  | Yes | 2.79 | 1.92 | 4.05 | <0.001 | 3.54 | 2.33 | 5.39 | <0.001 | 1.01 | 0.64 | 1.59 | 0.966 |
| **Malaria endemicity** | Moderate risk (*Pf*PR_2–10_ 5-40%) | 1.00 |  |  |  | 1.00 |  |  |  | 1.00 |  |  |  |
|  | High risk (*Pf*PR_2–10_ >40%) | 1.41 | 0.94 | 2.12 | 0.100 | 1.52 | 0.96 | 2.42 | 0.075 | 0.52 | 0.29 | 0.91 | 0.021 |
| **Source (public/private)** | Public | 1.00 |  |  |  | 1.00 |  |  |  | 1.00 |  |  |  |
|  | Private | 0.03 | 0.00 | 0.30 | 0.003 | 0.03 | 0.00 | 0.23 | 0.001 | 0.51 | 0.07 | 3.65 | 0.506 |
| **Source (level)** | Public facility, private clinic/doctor | 1.00 |  |  |  | 1.00 |  |  |  | 1.00 |  |  |  |
|  | All other sources | 11.75 | 1.26 | 109.14 | 0.030 | 13.09 | 2.03 | 84.29 | 0.007 | 0.39 | 0.06 | 2.75 | 0.347 |
| **Child's age (months)** | 0 - 5 | 0.55 | 0.22 | 1.40 | 0.213 | 0.75 | 0.26 | 2.18 | 0.603 | 1.35 | 0.54 | 3.38 | 0.516 |
|  | 6 - 11 | 1.00 |  |  |  | 1.00 |  |  |  | 1.00 |  |  |  |
|  | 12 - 23 | 1.52 | 0.88 | 2.64 | 0.136 | 1.38 | 0.73 | 2.62 | 0.318 | 1.23 | 0.64 | 2.35 | 0.531 |
|  | 24 - 35 | 2.47 | 1.38 | 4.42 | 0.002 | 2.16 | 1.12 | 4.16 | 0.022 | 0.71 | 0.34 | 1.48 | 0.356 |
|  | 36 - 47 | 3.06 | 1.63 | 5.76 | 0.001 | 2.24 | 1.11 | 4.53 | 0.025 | 1.38 | 0.64 | 2.98 | 0.411 |
|  | 48 - 59 | 2.06 | 1.05 | 4.02 | 0.035 | 1.96 | 0.92 | 4.18 | 0.082 | 0.94 | 0.39 | 2.23 | 0.883 |
| **Child's sex** | Male | 1.00 |  |  |  | 1.00 |  |  |  | 1.00 |  |  |  |
|  | Female | 1.00 | 0.71 | 1.43 | 0.980 | 0.98 | 0.66 | 1.45 | 0.914 | 1.08 | 0.70 | 1.68 | 0.715 |
| **Maternal age (years)** | 15 -24 | 1.00 |  |  |  | 1.00 |  |  |  | 1.00 |  |  |  |
|  | 25 - 29 | 1.18 | 0.73 | 1.88 | 0.501 | 1.36 | 0.81 | 2.30 | 0.249 | 1.13 | 0.64 | 1.97 | 0.678 |
|  | 30 - 34 | 1.37 | 0.80 | 2.34 | 0.253 | 1.27 | 0.69 | 2.35 | 0.435 | 0.54 | 0.26 | 1.11 | 0.095 |
|  | 35 - 39 | 0.99 | 0.53 | 1.85 | 0.981 | 1.16 | 0.58 | 2.34 | 0.676 | 1.11 | 0.52 | 2.38 | 0.788 |
|  | 40 - 49 | 1.14 | 0.57 | 2.30 | 0.710 | 1.05 | 0.49 | 2.22 | 0.905 | 0.88 | 0.34 | 2.27 | 0.790 |
| **Maternal education** | No education | 1.00 |  |  |  | 1.00 |  |  |  | 1.00 |  |  |  |
|  | Primary | 1.39 | 0.90 | 2.14 | 0.135 | 1.31 | 0.81 | 2.12 | 0.279 | 1.13 | 0.63 | 2.04 | 0.683 |
|  | Secondary or higher | 1.13 | 0.54 | 2.38 | 0.739 | 1.29 | 0.55 | 3.03 | 0.559 | 2.44 | 1.08 | 5.53 | 0.032 |
| **Residence** | Urban | 1.00 |  |  |  | 1.00 |  |  |  | 1.00 |  |  |  |
|  | Rural | 1.66 | 1.00 | 2.74 | 0.050 | 1.13 | 0.64 | 1.99 | 0.682 | 1.40 | 0.76 | 2.60 | 0.280 |
| **Household wealth** | Poorest | 1.00 |  |  |  | 1.00 |  |  |  | 1.00 |  |  |  |
|  | Second | 0.74 | 0.42 | 1.34 | 0.323 | 0.94 | 0.50 | 1.77 | 0.844 | 0.92 | 0.38 | 2.23 | 0.862 |
|  | Middle | 0.91 | 0.52 | 1.58 | 0.728 | 0.78 | 0.43 | 1.43 | 0.423 | 1.11 | 0.49 | 2.51 | 0.807 |
|  | Fourth | 0.42 | 0.23 | 0.79 | 0.006 | 0.36 | 0.18 | 0.72 | 0.004 | 1.14 | 0.49 | 2.66 | 0.757 |
|  | Least poor | 0.22 | 0.09 | 0.50 | <0.001 | 0.18 | 0.07 | 0.49 | 0.001 | 1.38 | 0.50 | 3.81 | 0.536 |
| **Household members** | 1-4 members | 1.00 |  |  |  | 1.00 |  |  |  | 1.00 |  |  |  |
|  | 5-8 members | 1.36 | 0.90 | 2.05 | 0.147 | 1.59 | 0.99 | 2.56 | 0.055 | 0.77 | 0.45 | 1.32 | 0.345 |
|  | 9-12 members | 1.03 | 0.54 | 1.96 | 0.927 | 1.57 | 0.76 | 3.24 | 0.220 | 3.02 | 1.54 | 5.92 | 0.001 |
|  | 13 or more members | 0.81 | 0.26 | 2.54 | 0.720 | 1.06 | 0.28 | 3.98 | 0.935 | 4.65 | 1.62 | 13.35 | 0.004 |
| **Health care access (money)** | Big problem | 1.00 |  |  |  | 1.00 |  |  |  | 1.00 |  |  |  |
|  | Not a big problem | 1.39 | 0.90 | 2.15 | 0.135 | 2.09 | 1.28 | 3.43 | 0.003 | 1.24 | 0.71 | 2.20 | 0.450 |
| **Health care access (distance)** | Big problem | 1.00 |  |  |  | 1.00 |  |  |  | 1.00 |  |  |  |
|  | Not a big problem | 0.88 | 0.56 | 1.39 | 0.591 | 0.73 | 0.44 | 1.21 | 0.227 | 0.79 | 0.45 | 1.41 | 0.428 |
| **Symptoms** | Fever alone | 1.00 |  |  |  | 1.00 |  |  |  | 1.00 |  |  |  |
|  | Fever, cough | 1.13 | 0.72 | 1.77 | 0.607 | 1.29 | 0.78 | 2.15 | 0.319 | 1.25 | 0.72 | 2.18 | 0.429 |
|  | Fever, cough, rapid breaths | 0.64 | 0.38 | 1.08 | 0.094 | 0.66 | 0.36 | 1.20 | 0.176 | 1.69 | 0.95 | 3.01 | 0.077 |
| **Malaria transmission season** | Off-peak | 1.00 |  |  |  | 1.00 |  |  |  | 1.00 |  |  |  |
|  | Peak | 2.33 | 1.13 | 4.79 | 0.021 | 0.71 | 0.28 | 1.78 | 0.465 | 0.29 | 0.09 | 0.95 | 0.041 |
| **Health card** | No (never had or lost) | 1.00 |  |  |  | 1.00 |  |  |  | 1.00 |  |  |  |
|  | Yes (seen or reported) | 0.70 | 0.35 | 1.40 | 0.312 | 0.86 | 0.39 | 1.89 | 0.712 | 2.48 | 0.68 | 9.00 | 0.168 |

CI refers to confidence interval. AOR refers to adjusted odds ratio. AORs based on mixed-effects logistic regression models adjusted for data clustering and all listed covariates. Some covariates may have been included in model with collapsed response categories, and if so, this is noted in the above table.

**Rwanda DHS 2010-2011**

|  |  | **Any anti-malarial use** | | | | **ACT Use** | | | | **Any antibiotic use** | | | |
| --- | --- | --- | --- | --- | --- | --- | --- | --- | --- | --- | --- | --- | --- |
|  |  | **AOR** | **95% CI** | | **pvalue** | **AOR** | **95% CI** | | **pvalue** | **AOR** | **95% CI** | | **pvalue** |
| **Diagnostic test use** | No | 1.00 |  |  |  | 1.00 |  |  |  | 1.00 |  |  |  |
|  | Yes | 0.83 | 0.48 | 1.44 | 0.506 | 0.88 | 0.51 | 1.51 | 0.633 | 2.95 | 1.82 | 4.79 | <0.001 |
| **Malaria endemicity** | Malaria-free | 0.09 | 0.02 | 0.34 | <0.001 | 0.10 | 0.03 | 0.36 | 0.001 | 2.57 | 1.37 | 4.81 | 0.003 |
|  | Low risk (*Pf*PR_2–10_ <5%) | 1.00 |  |  |  | 1.00 |  |  |  | 1.00 |  |  |  |
|  | Moderate risk (*Pf*PR_2–10_ 5-40%) | 2.43 | 1.24 | 4.77 | 0.010 | 2.17 | 1.12 | 4.21 | 0.022 | 0.40 | 0.22 | 0.73 | 0.003 |
| **Source (public/private)** | Public | 1.00 |  |  |  | 1.00 |  |  |  | 1.00 |  |  |  |
|  | Private | 0.13 | 0.05 | 0.35 | <0.001 | 0.12 | 0.04 | 0.33 | <0.001 | 0.43 | 0.24 | 0.75 | 0.003 |
| **Source (level)** | Hospital |  |  |  |  |  |  |  |  |  |  |  |  |
|  | Non-hospital formal medical |  |  |  |  |  |  |  |  |  |  |  |  |
|  | Community health worker |  |  |  |  |  |  |  |  |  |  |  |  |
|  | Pharmacy |  |  |  |  |  |  |  |  |  |  |  |  |
|  | Other |  |  |  |  |  |  |  |  |  |  |  |  |
| **Child's age (months)** | 0 - 5 | 0.54 | 0.10 | 3.02 | 0.483 | 0.29 | 0.03 | 2.68 | 0.277 | 0.69 | 0.26 | 1.87 | 0.471 |
|  | 6 - 11 | 1.00 |  |  |  | 1.00 |  |  |  | 1.00 |  |  |  |
|  | 12 - 23 | 1.95 | 0.83 | 4.60 | 0.126 | 2.00 | 0.85 | 4.72 | 0.112 | 0.70 | 0.37 | 1.31 | 0.266 |
|  | 24 - 35 | 1.60 | 0.66 | 3.86 | 0.295 | 1.54 | 0.63 | 3.72 | 0.341 | 0.67 | 0.35 | 1.30 | 0.237 |
|  | 36 - 47 | 1.93 | 0.74 | 5.05 | 0.178 | 2.07 | 0.80 | 5.39 | 0.136 | 0.44 | 0.21 | 0.92 | 0.030 |
|  | 48 - 59 | 3.48 | 1.22 | 9.94 | 0.020 | 3.14 | 1.12 | 8.80 | 0.030 | 0.50 | 0.23 | 1.10 | 0.087 |
| **Child's sex** | Male | 1.00 |  |  |  | 1.00 |  |  |  | 1.00 |  |  |  |
|  | Female | 0.92 | 0.55 | 1.55 | 0.765 | 0.88 | 0.53 | 1.47 | 0.623 | 0.98 | 0.65 | 1.48 | 0.913 |
| **Maternal age (years)** | 15 -24 | 1.00 |  |  |  | 1.00 |  |  |  | 1.00 |  |  |  |
|  | 25 - 29 | 1.97 | 0.92 | 4.21 | 0.082 | 2.05 | 0.94 | 4.44 | 0.070 | 1.14 | 0.66 | 1.96 | 0.649 |
|  | 30 - 34 | 1.49 | 0.63 | 3.50 | 0.366 | 1.35 | 0.57 | 3.20 | 0.488 | 1.33 | 0.71 | 2.49 | 0.366 |
|  | 35 - 39 | 2.98 | 1.11 | 8.03 | 0.031 | 2.89 | 1.09 | 7.70 | 0.033 | 0.60 | 0.28 | 1.26 | 0.176 |
|  | 40 - 49 | 1.84 | 0.63 | 5.44 | 0.267 | 2.02 | 0.68 | 5.98 | 0.205 | 1.40 | 0.59 | 3.31 | 0.447 |
| **Maternal education** | No education | 1.00 |  |  |  | 1.00 |  |  |  | 1.00 |  |  |  |
|  | Primary | 0.66 | 0.31 | 1.40 | 0.274 | 0.57 | 0.27 | 1.20 | 0.141 | 1.28 | 0.68 | 2.42 | 0.440 |
|  | Secondary or higher | 0.48 | 0.14 | 1.62 | 0.235 | 0.45 | 0.13 | 1.50 | 0.193 | 3.51 | 1.31 | 9.44 | 0.013 |
| **Residence** | Urban | 1.00 |  |  |  | 1.00 |  |  |  | 1.00 |  |  |  |
|  | Rural | 1.80 | 0.71 | 4.58 | 0.220 | 1.69 | 0.67 | 4.23 | 0.266 | 0.81 | 0.39 | 1.71 | 0.584 |
| **Household wealth** | Poorest | 1.00 |  |  |  | 1.00 |  |  |  | 1.00 |  |  |  |
|  | Second | 0.55 | 0.24 | 1.23 | 0.142 | 0.45 | 0.20 | 1.02 | 0.057 | 1.96 | 1.02 | 3.77 | 0.042 |
|  | Middle | 0.59 | 0.26 | 1.32 | 0.196 | 0.57 | 0.25 | 1.27 | 0.171 | 2.05 | 1.06 | 3.97 | 0.034 |
|  | Fourth | 0.34 | 0.14 | 0.83 | 0.018 | 0.35 | 0.14 | 0.85 | 0.020 | 1.25 | 0.63 | 2.48 | 0.521 |
|  | Least poor | 0.56 | 0.21 | 1.50 | 0.251 | 0.52 | 0.19 | 1.37 | 0.185 | 4.42 | 1.96 | 9.99 | <0.001 |
| **Household members** | 1-4 members | 1.00 |  |  |  | 1.00 |  |  |  | 1.00 |  |  |  |
|  | 5-8 members | 0.65 | 0.36 | 1.20 | 0.167 | 0.68 | 0.38 | 1.24 | 0.212 | 0.96 | 0.61 | 1.52 | 0.861 |
|  | 9 or more members | 0.47 | 0.13 | 1.75 | 0.264 | 0.54 | 0.15 | 1.91 | 0.335 | 1.90 | 0.77 | 4.67 | 0.161 |
| **Health care access (money)** | Big problem | 1.00 |  |  |  | 1.00 |  |  |  | 1.00 |  |  |  |
|  | Not a big problem | 1.33 | 0.75 | 2.36 | 0.328 | 1.35 | 0.76 | 2.38 | 0.307 | 0.73 | 0.47 | 1.15 | 0.175 |
| **Health care access (distance)** | Big problem | 1.00 |  |  |  | 1.00 |  |  |  | 1.00 |  |  |  |
|  | Not a big problem | 0.86 | 0.48 | 1.54 | 0.613 | 0.88 | 0.49 | 1.57 | 0.662 | 1.01 | 0.63 | 1.63 | 0.957 |
| **Symptoms** | Fever alone | 1.00 |  |  |  | 1.00 |  |  |  | 1.00 |  |  |  |
|  | Fever, cough | 0.51 | 0.27 | 0.96 | 0.038 | 0.50 | 0.26 | 0.94 | 0.032 | 2.47 | 1.42 | 4.32 | 0.001 |
|  | Fever, cough, rapid breaths | 0.35 | 0.18 | 0.66 | 0.001 | 0.32 | 0.17 | 0.62 | 0.001 | 4.35 | 2.50 | 7.56 | <0.001 |
| **Malaria transmission season** | Off-peak | 1.00 |  |  |  | 1.00 |  |  |  | 1.00 |  |  |  |
|  | Peak | 1.90 | 1.02 | 3.55 | 0.045 | 1.94 | 1.05 | 3.58 | 0.035 | 1.05 | 0.62 | 1.79 | 0.860 |
| **Health card** | No (never had or lost) | 1.00 |  |  |  | 1.00 |  |  |  | 1.00 |  |  |  |
|  | Yes (seen or reported) | 0.86 | 0.41 | 1.78 | 0.678 | 0.78 | 0.38 | 1.60 | 0.499 | 1.36 | 0.72 | 2.58 | 0.349 |

CI refers to confidence interval. AOR refers to adjusted odds ratio. AORs based on mixed-effects logistic regression models adjusted for data clustering and all listed covariates. Some covariates may have been included in model with collapsed response categories, and if so, this is noted in the above table.

**Senegal DHS 2010-2011**

|  |  | **Any anti-malarial use** | | | | **ACT Use** | | | | **Any antibiotic use** | | | |
| --- | --- | --- | --- | --- | --- | --- | --- | --- | --- | --- | --- | --- | --- |
|  |  | **AOR** | **95% CI** | | **pvalue** | **AOR** | **95% CI** | | **pvalue** | **AOR** | **95% CI** | | **pvalue** |
| **Diagnostic test use** | No | 1.00 |  |  |  | 1.00 |  |  |  | 1.00 |  |  |  |
|  | Yes | 1.69 | 1.04 | 2.76 | 0.036 | 2.99 | 1.32 | 6.79 | 0.009 | 1.50 | 0.97 | 2.31 | 0.070 |
| **Malaria endemicity** | Low risk (*Pf*PR_2–10_ <5%) | 1.00 |  |  |  | 1.00 |  |  |  | 1.00 |  |  |  |
|  | Moderate risk (*Pf*PR_2–10_ 5-40%) | 1.03 | 0.60 | 1.77 | 0.904 | 1.06 | 0.39 | 2.91 | 0.904 | 0.80 | 0.47 | 1.35 | 0.396 |
|  | High risk (*Pf*PR_2–10_ >40%) | 1.60 | 0.30 | 8.50 | 0.583 | 1.60 | 0.07 | 34.80 | 0.765 | 0.28 | 0.05 | 1.72 | 0.169 |
| **Source (public/private)** | Public | 1.00 |  |  |  | 1.00 |  |  |  | 1.00 |  |  |  |
|  | Private | 0.11 | 0.01 | 0.88 | 0.038 | 0.68 | 0.13 | 3.58 | 0.649 | 0.51 | 0.19 | 1.33 | 0.167 |
| **Source (level)** | Hospital | 1.00 |  |  |  | 1.00 |  |  |  | 1.00 |  |  |  |
|  | Non-hospital formal medical | 1.30 | 0.68 | 2.49 | 0.423 | 2.11 | 0.64 | 6.94 | 0.217 | 1.44 | 0.86 | 2.41 | 0.162 |
|  | Community health worker | 1.52 | 0.44 | 5.23 | 0.509 |  |  |  |  | 0.74 | 0.27 | 2.01 | 0.558 |
|  | Pharmacy | 13.30 | 1.43 | 123.83 | 0.023 |  |  |  |  | 0.67 | 0.22 | 2.01 | 0.471 |
|  | Other | 5.64 | 0.59 | 53.58 | 0.132 | 2.38 | 0.39 | 14.49 | 0.348 | 0.36 | 0.12 | 1.09 | 0.071 |
| **Child's age (months)** | 0 - 5 | 0.71 | 0.31 | 1.67 | 0.436 | 1.38 | 0.29 | 6.55 | 0.682 | 0.95 | 0.50 | 1.79 | 0.863 |
|  | 6 - 11 | 1.00 |  |  |  | 1.00 |  |  |  | 1.00 |  |  |  |
|  | 12 - 23 | 0.87 | 0.45 | 1.68 | 0.675 | 1.40 | 0.40 | 4.99 | 0.600 | 1.40 | 0.84 | 2.35 | 0.197 |
|  | 24 - 35 | 1.63 | 0.86 | 3.10 | 0.136 | 2.62 | 0.77 | 8.88 | 0.122 | 1.40 | 0.82 | 2.38 | 0.219 |
|  | 36 - 47 | 1.55 | 0.80 | 3.02 | 0.196 | 2.44 | 0.71 | 8.43 | 0.159 | 1.24 | 0.71 | 2.14 | 0.451 |
|  | 48 - 59 | 1.42 | 0.65 | 3.08 | 0.377 | 2.06 | 0.48 | 8.81 | 0.331 | 1.62 | 0.86 | 3.05 | 0.136 |
| **Child's sex** | Male | 1.00 |  |  |  | 1.00 |  |  |  | 1.00 |  |  |  |
|  | Female | 0.71 | 0.48 | 1.05 | 0.088 | 1.21 | 0.63 | 2.33 | 0.566 | 1.08 | 0.79 | 1.48 | 0.643 |
| **Maternal age (years)** | 15 -24 | 1.00 |  |  |  | 1.00 |  |  |  | 1.00 |  |  |  |
|  | 25 - 29 | 1.07 | 0.62 | 1.87 | 0.801 | 0.56 | 0.19 | 1.67 | 0.297 | 0.79 | 0.52 | 1.21 | 0.282 |
|  | 30 - 34 | 1.54 | 0.89 | 2.67 | 0.122 | 1.90 | 0.76 | 4.79 | 0.171 | 0.65 | 0.41 | 1.03 | 0.065 |
|  | 35 - 39 | 1.21 | 0.65 | 2.27 | 0.548 | 1.96 | 0.71 | 5.37 | 0.192 | 0.80 | 0.48 | 1.32 | 0.381 |
|  | 40 - 49 | 1.47 | 0.67 | 3.23 | 0.335 | 1.67 | 0.44 | 6.24 | 0.449 | 0.96 | 0.50 | 1.84 | 0.907 |
| **Maternal education** | No education | 1.00 |  |  |  | 1.00 |  |  |  | 1.00 |  |  |  |
|  | Primary | 1.40 | 0.87 | 2.25 | 0.167 | 1.86 | 0.83 | 4.18 | 0.133 | 0.96 | 0.63 | 1.44 | 0.831 |
|  | Secondary or higher | 0.93 | 0.47 | 1.86 | 0.846 | 1.27 | 0.39 | 4.10 | 0.687 | 0.78 | 0.44 | 1.38 | 0.390 |
| **Residence** | Urban | 1.00 |  |  |  | 1.00 |  |  |  | 1.00 |  |  |  |
|  | Rural | 0.98 | 0.53 | 1.79 | 0.936 | 0.73 | 0.25 | 2.17 | 0.573 | 1.02 | 0.57 | 1.81 | 0.953 |
| **Household wealth** | Poorest | 1.00 |  |  |  | 1.00 |  |  |  | 1.00 |  |  |  |
|  | Second | 1.09 | 0.59 | 2.03 | 0.785 | 0.84 | 0.26 | 2.74 | 0.768 | 1.39 | 0.82 | 2.37 | 0.225 |
|  | Middle | 0.78 | 0.37 | 1.61 | 0.496 | 1.04 | 0.28 | 3.84 | 0.952 | 1.24 | 0.66 | 2.30 | 0.503 |
|  | Fourth | 1.03 | 0.45 | 2.37 | 0.945 | 1.02 | 0.23 | 4.54 | 0.980 | 2.23 | 1.09 | 4.54 | 0.028 |
|  | Least poor | 1.53 | 0.62 | 3.79 | 0.357 | 1.64 | 0.32 | 8.35 | 0.551 | 2.37 | 1.06 | 5.33 | 0.036 |
| **Household members** | 1-4 members | 1.00 |  |  |  | 1.00 |  |  |  | 1.00 |  |  |  |
|  | 5-8 members | 0.50 | 0.18 | 1.41 | 0.192 | 0.20 | 0.04 | 0.99 | 0.049 | 0.96 | 0.39 | 2.35 | 0.930 |
|  | 9-12 members | 0.81 | 0.29 | 2.20 | 0.673 | 0.48 | 0.11 | 2.08 | 0.328 | 0.70 | 0.29 | 1.71 | 0.430 |
|  | 13 or more members | 0.91 | 0.35 | 2.41 | 0.855 | 0.61 | 0.15 | 2.45 | 0.489 | 1.06 | 0.45 | 2.51 | 0.895 |
| **Health care access (money)** | Big problem | 1.00 |  |  |  | 1.00 |  |  |  | 1.00 |  |  |  |
|  | Not a big problem | 0.78 | 0.51 | 1.19 | 0.246 | 0.46 | 0.22 | 0.99 | 0.048 | 1.10 | 0.77 | 1.57 | 0.597 |
| **Health care access (distance)** | Big problem | 1.00 |  |  |  | 1.00 |  |  |  | 1.00 |  |  |  |
|  | Not a big problem | 1.00 | 0.62 | 1.64 | 0.985 | 1.10 | 0.47 | 2.58 | 0.830 | 0.77 | 0.51 | 1.17 | 0.219 |
| **Symptoms** | Fever alone | 1.00 |  |  |  | 1.00 |  |  |  | 1.00 |  |  |  |
|  | Fever, cough | 0.81 | 0.47 | 1.38 | 0.430 | 0.79 | 0.32 | 1.99 | 0.624 | 1.32 | 0.86 | 2.03 | 0.210 |
|  | Fever, cough, rapid breaths | 0.83 | 0.54 | 1.28 | 0.395 | 0.73 | 0.35 | 1.54 | 0.415 | 1.74 | 1.22 | 2.50 | 0.002 |
| **Malaria transmission season** | Off-peak | 1.00 |  |  |  | 1.00 |  |  |  | 1.00 |  |  |  |
|  | Peak | 1.69 | 0.84 | 3.39 | 0.142 | 2.21 | 0.62 | 7.82 | 0.220 | 0.71 | 0.34 | 1.48 | 0.364 |
| **Health card** | No (never had or lost) | 1.00 |  |  |  | 1.00 |  |  |  | 1.00 |  |  |  |
|  | Yes (seen or reported) | 0.74 | 0.28 | 1.96 | 0.549 | 0.34 | 0.08 | 1.49 | 0.152 | 0.94 | 0.39 | 2.28 | 0.893 |

CI refers to confidence interval. AOR refers to adjusted odds ratio. AORs based on mixed-effects logistic regression models adjusted for data clustering and all listed covariates. Some covariates may have been included in model with collapsed response categories, and if so, this is noted in the above table.

**Uganda DHS 2011**

|  |  | **Any anti-malarial use** | | | | **ACT Use** | | | | **Any antibiotic use** | | | |
| --- | --- | --- | --- | --- | --- | --- | --- | --- | --- | --- | --- | --- | --- |
|  |  | **AOR** | **95% CI** | | **pvalue** | **AOR** | **95% CI** | | **pvalue** | **AOR** | **95% CI** | | **pvalue** |
| **Diagnostic test use** | No | 1.00 |  |  |  | 1.00 |  |  |  | 1.00 |  |  |  |
|  | Yes | 1.24 | 0.96 | 1.61 | 0.097 | 0.84 | 0.66 | 1.06 | 0.133 | 1.37 | 1.09 | 1.72 | 0.007 |
| **Malaria endemicity** | Malaria-free or low risk (*Pf*PR_2–10_ <5%) | 0.10 | 0.02 | 0.42 | 0.002 | 0.06 | 0.01 | 0.35 | 0.002 | 1.00 | 0.28 | 3.55 | 0.998 |
|  | Moderate risk (*Pf*PR_2–10_ 5-40%) | 1.00 |  |  |  | 1.00 |  |  |  | 1.00 |  |  |  |
|  | High risk (*Pf*PR_2–10_ >40%) | 0.75 | 0.53 | 1.07 | 0.117 | 0.84 | 0.61 | 1.15 | 0.270 | 1.35 | 1.01 | 1.81 | 0.042 |
| **Source (public/private)** | Public | 1.00 |  |  |  | 1.00 |  |  |  | 1.00 |  |  |  |
|  | Private | 0.30 | 0.19 | 0.49 | <0.001 | 0.38 | 0.26 | 0.57 | <0.001 | 1.02 | 0.69 | 1.50 | 0.935 |
| **Source (level)** | Hospital | 1.00 |  |  |  | 1.00 |  |  |  | 1.00 |  |  |  |
|  | Non-hospital formal medical | 0.82 | 0.50 | 1.34 | 0.429 | 1.49 | 0.99 | 2.25 | 0.057 | 0.75 | 0.50 | 1.12 | 0.157 |
|  | Community health worker | 3.62 | 1.26 | 10.39 | 0.017 | 7.31 | 2.89 | 18.47 | <0.001 | 0.49 | 0.21 | 1.12 | 0.090 |
|  | Pharmacy | 1.14 | 0.54 | 2.39 | 0.731 | 0.44 | 0.20 | 0.97 | 0.042 | 0.35 | 0.16 | 0.75 | 0.007 |
|  | Other | 0.46 | 0.28 | 0.76 | 0.002 | 0.67 | 0.40 | 1.10 | 0.111 | 0.80 | 0.48 | 1.32 | 0.381 |
| **Child's age (months)** | 0 - 5 | 0.24 | 0.15 | 0.39 | <0.001 | 0.20 | 0.12 | 0.35 | <0.001 | 1.85 | 1.20 | 2.87 | 0.006 |
|  | 6 - 11 | 1.00 |  |  |  | 1.00 |  |  |  | 1.00 |  |  |  |
|  | 12 - 23 | 1.41 | 0.97 | 2.03 | 0.068 | 1.44 | 1.02 | 2.03 | 0.036 | 0.77 | 0.55 | 1.07 | 0.121 |
|  | 24 - 35 | 1.44 | 0.99 | 2.10 | 0.053 | 1.41 | 1.00 | 2.00 | 0.051 | 0.73 | 0.52 | 1.03 | 0.071 |
|  | 36 - 47 | 1.34 | 0.92 | 1.97 | 0.131 | 1.41 | 0.99 | 2.02 | 0.060 | 0.66 | 0.46 | 0.94 | 0.021 |
|  | 48 - 59 | 1.61 | 1.08 | 2.40 | 0.020 | 1.48 | 1.02 | 2.14 | 0.038 | 0.62 | 0.43 | 0.89 | 0.010 |
| **Child's sex** | Male | 1.00 |  |  |  | 1.00 |  |  |  | 1.00 |  |  |  |
|  | Female | 1.13 | 0.91 | 1.40 | 0.263 | 0.95 | 0.78 | 1.15 | 0.576 | 1.20 | 0.98 | 1.46 | 0.074 |
| **Maternal age (years)** | 15 -24 | 1.00 |  |  |  | 1.00 |  |  |  | 1.00 |  |  |  |
|  | 25 - 29 | 0.92 | 0.69 | 1.23 | 0.586 | 0.89 | 0.69 | 1.17 | 0.412 | 1.24 | 0.95 | 1.61 | 0.108 |
|  | 30 - 34 | 0.92 | 0.65 | 1.29 | 0.625 | 1.18 | 0.86 | 1.62 | 0.297 | 1.03 | 0.75 | 1.41 | 0.847 |
|  | 35 - 39 | 0.80 | 0.55 | 1.17 | 0.256 | 1.09 | 0.77 | 1.54 | 0.640 | 1.00 | 0.71 | 1.41 | 0.998 |
|  | 40 - 49 | 1.25 | 0.76 | 2.06 | 0.380 | 1.03 | 0.67 | 1.58 | 0.903 | 0.83 | 0.53 | 1.29 | 0.401 |
| **Maternal education** | No education | 1.00 |  |  |  | 1.00 |  |  |  | 1.00 |  |  |  |
|  | Primary | 1.72 | 1.23 | 2.41 | 0.002 | 1.20 | 0.88 | 1.65 | 0.252 | 0.90 | 0.66 | 1.24 | 0.522 |
|  | Secondary or higher | 1.21 | 0.79 | 1.87 | 0.383 | 1.28 | 0.86 | 1.93 | 0.228 | 1.17 | 0.79 | 1.74 | 0.435 |
| **Residence** | Urban | 1.00 |  |  |  | 1.00 |  |  |  | 1.00 |  |  |  |
|  | Rural | 1.57 | 0.98 | 2.50 | 0.058 | 1.12 | 0.74 | 1.70 | 0.585 | 0.92 | 0.62 | 1.36 | 0.669 |
| **Household wealth** | Poorest | 1.00 |  |  |  | 1.00 |  |  |  | 1.00 |  |  |  |
|  | Second | 1.28 | 0.90 | 1.82 | 0.163 | 1.30 | 0.95 | 1.78 | 0.100 | 1.08 | 0.79 | 1.48 | 0.623 |
|  | Middle | 1.18 | 0.81 | 1.72 | 0.390 | 1.23 | 0.87 | 1.73 | 0.241 | 1.35 | 0.96 | 1.90 | 0.080 |
|  | Fourth | 1.15 | 0.77 | 1.71 | 0.508 | 1.17 | 0.81 | 1.71 | 0.400 | 1.13 | 0.78 | 1.62 | 0.515 |
|  | Least poor | 1.69 | 1.01 | 2.81 | 0.045 | 1.75 | 1.10 | 2.76 | 0.017 | 1.90 | 1.22 | 2.95 | 0.004 |
| **Household members** | 1-4 members | 1.00 |  |  |  | 1.00 |  |  |  | 1.00 |  |  |  |
|  | 5-8 members | 1.11 | 0.83 | 1.48 | 0.499 | 1.02 | 0.78 | 1.33 | 0.906 | 0.79 | 0.61 | 1.03 | 0.077 |
|  | 9-12 members | 1.04 | 0.71 | 1.51 | 0.846 | 1.30 | 0.93 | 1.83 | 0.127 | 0.90 | 0.65 | 1.25 | 0.523 |
|  | 13 or more members | 2.00 | 0.99 | 4.02 | 0.053 | 0.83 | 0.41 | 1.68 | 0.606 | 1.54 | 0.83 | 2.85 | 0.171 |
| **Health care access (money)** | Big problem | 1.00 |  |  |  | 1.00 |  |  |  | 1.00 |  |  |  |
|  | Not a big problem | 0.97 | 0.76 | 1.24 | 0.789 | 0.88 | 0.70 | 1.10 | 0.249 | 0.90 | 0.72 | 1.12 | 0.347 |
| **Health care access (distance)** | Big problem | 1.00 |  |  |  | 1.00 |  |  |  | 1.00 |  |  |  |
|  | Not a big problem | 1.04 | 0.82 | 1.34 | 0.732 | 1.05 | 0.84 | 1.32 | 0.672 | 1.07 | 0.86 | 1.34 | 0.546 |
| **Symptoms** | Fever alone | 1.00 |  |  |  | 1.00 |  |  |  | 1.00 |  |  |  |
|  | Fever, cough | 0.88 | 0.67 | 1.16 | 0.374 | 0.96 | 0.75 | 1.24 | 0.781 | 2.38 | 1.84 | 3.09 | <0.001 |
|  | Fever, cough, rapid breaths | 0.73 | 0.56 | 0.95 | 0.021 | 0.74 | 0.58 | 0.94 | 0.015 | 2.94 | 2.28 | 3.77 | <0.001 |
| **Malaria transmission season** | Off-peak | 1.00 |  |  |  | 1.00 |  |  |  | 1.00 |  |  |  |
|  | Peak | 1.69 | 1.05 | 2.72 | 0.031 | 1.30 | 0.84 | 2.01 | 0.239 | 1.00 | 0.67 | 1.50 | 0.999 |
| **Health card** | No (never had or lost) | 1.00 |  |  |  | 1.00 |  |  |  | 1.00 |  |  |  |
|  | Yes (seen or reported) | 1.05 | 0.77 | 1.44 | 0.748 | 1.09 | 0.82 | 1.44 | 0.574 | 0.86 | 0.65 | 1.14 | 0.288 |

CI refers to confidence interval. AOR refers to adjusted odds ratio. AORs based on mixed-effects logistic regression models adjusted for data clustering and all listed covariates. Some covariates may have been included in model with collapsed response categories, and if so, this is noted in the above table.

**Zimbabwe DHS 2010-2011**

|  |  | **Any anti-malarial use** | | | | **ACT Use** | | | | **Any antibiotic use** | | | |
| --- | --- | --- | --- | --- | --- | --- | --- | --- | --- | --- | --- | --- | --- |
|  |  | **AOR** | **95% CI** | | **pvalue** | **AOR** | **95% CI** | | **pvalue** | **AOR** | **95% CI** | | **pvalue** |
| **Diagnostic test use** | No | 1.00 |  |  |  | 1.00 |  |  |  | 1.00 |  |  |  |
|  | Yes | 170.98 | 0.30 | 98480.04 | 0.113 | 25.55 | 1.69 | 385.68 | 0.019 | 0.55 | 0.20 | 1.51 | 0.244 |
| **Malaria endemicity** | Malaria-free or low risk (*Pf*PR_2–10_ <5%) | 1.00 |  |  |  |  |  |  |  |  |  |  |  |
|  | Moderate or high risk (*Pf*PR_2–10_ >5%) | 21.65 | 0.06 | 7723.58 | 0.305 | 1.77 | 0.12 | 25.12 | 0.675 | 1.63 | 0.77 | 3.48 | 0.203 |
| **Source (public/private)** | Public | 1.00 |  |  |  | 1.00 |  |  |  | 1.00 |  |  |  |
|  | Private | 0.04 | 0.00 | 11.52 | 0.268 | 5.30 | 0.48 | 58.77 | 0.174 | 0.89 | 0.26 | 3.07 | 0.857 |
| **Source (level)** | Hospital | 1.00 |  |  |  |  |  |  |  | 1.00 |  |  |  |
|  | Non-hospital formal medical | 0.02 | 0.00 | 10.00 | 0.222 |  |  |  |  | 1.00 | 0.40 | 2.49 | 0.993 |
|  | Other or pharmacy or CHW | 16.78 | 0.03 | 9612.84 | 0.384 |  |  |  |  | 0.51 | 0.14 | 1.84 | 0.304 |
| **Child's age (months)** | 0 - 11 | 1.00 |  |  |  | 1.00 |  |  |  | 1.00 |  |  |  |
|  | 12 - 59 | 18.64 | 0.06 | 5407.63 | 0.312 | 0.70 | 0.05 | 10.29 | 0.797 | 1.12 | 0.56 | 2.24 | 0.745 |
| **Child's sex** | Male | 1.00 |  |  |  | 1.00 |  |  |  | 1.00 |  |  |  |
|  | Female | 18.05 | 0.23 | 1440.52 | 0.195 | 1.69 | 0.22 | 12.95 | 0.611 | 1.04 | 0.55 | 1.96 | 0.915 |
| **Maternal age (years)** | 15 -24 | 1.00 |  |  |  | 1.00 |  |  |  | 1.00 |  |  |  |
|  | 25 - 29 | 31.10 | 0.11 | 8581.79 | 0.231 | 0.40 | 0.02 | 8.77 | 0.563 | 1.04 | 0.47 | 2.30 | 0.928 |
|  | 30 - 34 | 6.78 | 0.03 | 1798.13 | 0.501 | 5.59 | 0.49 | 63.79 | 0.166 | 1.45 | 0.54 | 3.86 | 0.463 |
|  | 35 - 49 | 4.67 | 0.00 | 5150.13 | 0.666 | 0.00 | 0.00 | . | 0.995 | 0.71 | 0.25 | 2.02 | 0.526 |
| **Maternal education** | None or primary education | 1.00 |  |  |  |  |  |  |  | 1.00 |  |  |  |
|  | Secondary or higher | 0.07 | 0.00 | 13.21 | 0.313 |  |  |  |  | 1.20 | 0.56 | 2.57 | 0.643 |
| **Residence** | Urban | 1.00 |  |  |  | 1.00 |  |  |  | 1.00 |  |  |  |
|  | Rural | 0.02 | 0.00 | 18.01 | 0.259 | 0.28 | 0.01 | 7.01 | 0.435 | 1.45 | 0.56 | 3.78 | 0.447 |
| **Household wealth** | Poorest or second | 1.00 |  |  |  | 1.00 |  |  |  | 1.00 |  |  |  |
|  | Middle or fourth or least poor | 0.81 | 0.00 | 191.69 | 0.938 | 1.50 | 0.06 | 36.36 | 0.803 | 1.80 | 0.81 | 4.00 | 0.151 |
| **Household members** | 1-4 members |  |  |  |  |  |  |  |  |  |  |  |  |
|  | 5-8 members |  |  |  |  |  |  |  |  |  |  |  |  |
|  | 9-12 members |  |  |  |  |  |  |  |  |  |  |  |  |
|  | 13 or more members |  |  |  |  |  |  |  |  |  |  |  |  |
| **Health care access (money)** | Big problem |  |  |  |  |  |  |  |  |  |  |  |  |
|  | Not a big problem |  |  |  |  |  |  |  |  |  |  |  |  |
| **Health care access (distance)** | Big problem |  |  |  |  |  |  |  |  |  |  |  |  |
|  | Not a big problem |  |  |  |  |  |  |  |  |  |  |  |  |
| **Symptoms** | Fever alone | 1.00 |  |  |  |  |  |  |  | 1.00 |  |  |  |
|  | Fever, cough | 0.89 | 0.01 | 69.86 | 0.958 |  |  |  |  | 2.30 | 0.97 | 5.42 | 0.058 |
|  | Fever, cough, rapid breaths | 0.06 | 0.00 | 18.53 | 0.331 |  |  |  |  | 1.93 | 0.88 | 4.23 | 0.101 |
| **Malaria transmission season** | Off-peak | 1.00 |  |  |  | 1.00 |  |  |  | 1.00 |  |  |  |
|  | Peak | 1.68 | 0.01 | 304.28 | 0.844 | 0.48 | 0.03 | 7.72 | 0.608 | 0.88 | 0.39 | 1.95 | 0.744 |
| **Health card** | No (never had or lost) | 1.00 |  |  |  | 1.00 |  |  |  | 1.00 |  |  |  |
|  | Yes (seen or reported) | 0.23 | 0.00 | 48.29 | 0.590 | 0.14 | 0.01 | 3.74 | 0.242 | 1.44 | 0.44 | 4.71 | 0.547 |

CI refers to confidence interval. AOR refers to adjusted odds ratio. AORs based on mixed-effects logistic regression models adjusted for data clustering and all listed covariates. Zimbabwe results should be interpreted with caution given few observations and positive outcomes for malaria treatment. Some covariates were included in model with collapsed response categories as noted above. Blank cells in the results table indicate the covariate could not be included in the final model.
