# Supplementary material for: Effect of diagnostic testing on medicines used by febrile children less than five years in 12 malaria-endemic African countries: a mixed-methods study
Source: Malar J. 2015 May 10;14:194. doi: 10.1186/s12936-015-0709-0 (PMC4432948; doi:10.1186/s12936-015-0709-0)
Supplement: Additional file 3: — Country case studies for Benin, Burundi, Malawi, Mozambique, Rwanda, Uganda. [file 12936_2015_709_MOESM3_ESM.docx]

**Additional File 3: Case study summaries for Benin, Burundi, Malawi, Mozambique, Rwanda, Uganda**

**Benin**

In Benin, there was slightly higher odds of any anti-malarial or ACT use for tested pediatric fevers compared to untested cases, and no significant difference was found in antibiotic treatment across these groups (Table 2). At the sub-national level, data suggest differences in the effect of diagnostic testing on any anti-malarial use by malaria risk and symptoms (Table 3).

Wide-scale mRDT deployment began around 2009 and data in our analysis largely reflects testing by mRDT. Importantly, however, the National Malaria Control Program only updated its treatment guidance in 2011 to recommend test-based treatment of children less than age five, which is just prior to the time of survey fieldwork for this analysis [44]. Moreover, in May 2011, a new policy was announced to make all malaria treatment free-of-charge to children under five years and pregnant women.

A recent national facility study that found malaria diagnostic services (either mRDT or microscopy) were available throughout sampled facilities, with mRDT constituting nearly two-thirds of testing available in the sample [9]. Community health workers did not use mRDT at the time of our analysis [44]. The national facility survey, however, may have disproportionately sampled facilities with higher attendance that may also have better commodity stocks than more remote facilities [9]. Diagnostic test availability, therefore, could be lower than reported in that study. Program documents also indicate more common mRDT stock-outs or expired supplies around the time our data was collected [44]. If the latter is the case, this suggests that tested febrile children in Benin may attend locations that have better access to medicines, which could confound the relationship between diagnostic testing and treatment. Moreover, stock-outs could further concentrate diagnostic services at higher-level facilities with microscopy such that children reaching these locations have more severe illness, which could also increase their likelihood of treatment.

It is also not clear how the new national guidelines of test-based treatment for under-fives were being implemented at this time, particularly since these guidelines were announced in 2011 around the time of survey fieldwork. In addition, the policy of free malaria treatment for children announced in May 2011 could also increase treatment likelihood for this age group, irrespective of whether a diagnostic test was performed or not.

The extensive use of private drug shops in Benin could also affect results in two ways, although we tried to account for this issue in our analysis (see methods section). First, caregivers may self-treat sick children either before or after visiting facilities where testing occurs. Second, such practices delay care seeking to formal providers such that children are more severely ill once they reach locations with diagnostic services, and are more likely to receive treatment. *‘In Benin care-seeking often goes: child has fever, taken to shop, gets medicines, doesn’t get better, goes to facility (where finally tested)’*

In terms of case management practices, the national facility study indicated that nearly 4 in 10 test-negative patients were prescribed ACTs during observed consultations [9] with plausibly worse adherence for young children [20] and in routine program conditions [24]. Program documents also suggest poor case management practices as well [44].

Finally, sub-national differences in the effect of diagnostic testing on any anti-malarial use across malaria risk contexts and by child’s symptoms are difficult to interpret. The issue of illness severity was put forward to potentially explain the different effect of diagnostic testing on malaria treatment by child’s symptoms. Still, this result is difficult to explain and may merit further investigation in facility-based research or qualitative studies. For malaria risk, this result is consistent with other country findings such that tested pediatric fevers in high-risk areas have higher odds of malaria treatment than untested cases, while there is a non-significant reduction in treatment odds associated with testing in moderate-risk settings. This result, too, is difficult to explain and merits attention in future qualitative studies or facility-based research.

**Burundi**

Burundi had relatively high odds of malaria treatment for tested pediatric fevers compared to untested cases among all studied countries (Table 2). At the sub-national level, there were significant differences in antibiotic treatment among tested pediatric fevers and those untested across low- and moderate-risk areas. In moderate risk areas, the odds of receiving antibiotic treatment declined by 66% for tested pediatric fevers compared to untested ones (OR: 0.44, 95% CI: 0.32-0.60). In contrast, there were significantly higher odds of antibiotic treatment associated with testing in low-risk areas (OR: 6.75, 95% CI: 1.30-35.00), although the strong effect should be interpreted with caution due to few observations and positive outcomes. There is limited published evidence to explain these results. Overall, study findings were perceived as plausible since data reflect a very early assessment within a broader malaria control program that has greatly expanded its work since that time. *“2010 is really a baseline year for a lot of malaria program work, and even the first time there was a DHS since 1987. Burundi just rolled out IPTp in 2014 after the policy change in 2013.”*

Burundi has quite variable malaria epidemiology within its borders where malaria is highly endemic in the north and west, but mostly malaria-free in the southern areas [44]. Wide-scale mRDT deployment started in 2012, and data in our analysis largely reflects testing by microscopy that is concentrated at referral hospitals. Therefore, febrile children tested for malaria likely reflect referred cases with more severe illness, which confounds the relationship between testing and treatment. While there is no published evidence, adherence to test-negative results using microscopy was plausibly poor and in line with findings from other countries [20,39]. In 2010, supply chains were also quite weak with disproportionate stock outages at peripheral clinics since hospitals maintain a separate supply system (although stock-outs at hospitals could also occur). This further suggests that febrile children attending hospitals are generally in locations with both ACTs and diagnostic services while untested children may generally visit locations with neither. Finally, there is generally quite poor access to health services in Burundi with communities living in remote, hilly areas coupled with poor roads and transportation networks. Despite this poor access to formal care, there were no community-based treatment programs in 2010, and drug shops are not generally used as an alternative to formal health care [44]. This may further reinforce the conclusion that febrile children reaching hospitals with diagnostic services are more severely ill and are more likely to be treated as well. ‘*(In Burundi) microscopy is basically in hospitals and healthcare access is difficult - remote, hilly communities. These results really show poor access to care’*

**Malawi**

In Malawi, tested pediatric fevers had significantly higher odds of receiving any anti-malarial drug compared to untested cases, while there was no significant difference for ACT or antibiotic treatment outcomes (Table 2). At the sub-national level, there were differences in the effect of diagnostic testing on malaria treatment by malaria risk and child’s symptoms, and for antibiotic treatment by source of care and child’s symptoms (Table 3). Overall, findings were perceived as plausible at the population level but difficult to explain, and may merit investigation in future qualitative or facility-based research *“I think antibiotic prescriptions could be something to examine more systematically in the next facility study and how testing is done for children with fever and other symptoms”*

Malaria transmission occurs throughout the year in most parts of Malawi with particularly high transmission in areas along Lake Malawi and the lowland Shire River Valley [41,44]. Wide-scale mRDT provision started in end-2011, and data in our study largely reflects testing by microscopy that is generally concentrated at district or rural hospitals. ACT stocks were also more frequently available at these hospitals compared to peripheral facilities [20], and it is therefore likely that tested cases had better access to medicines than untested cases. In addition, febrile children referred to district or rural hospitals may also have more severe illness, which could confound the relationship between testing and treatment. Finally, a national facility study documented poor adherence to test-negative results for facility blood smears that could also explain results in our study [20].

For malaria risk, this sub-national result is consistent with other country findings such that tested pediatric fevers in high-risk areas have higher odds of malaria treatment than untested cases, while there is a non-significant reduction in treatment odds associated with testing in moderate-risk settings. Still, this result is difficult to explain and may merit further investigation in future qualitative studies or facility-based research.

For source of care, there was slightly higher odds of antibiotic treatment (OR: 1.36, 95% CI: 0.95-1.96) among tested pediatric fevers compared to untested at private sources, while there was non-significant lower odds of antibiotic treatment associated with testing at public sources (OR: 0.85, 95% CI: 0.67-1.09). There is a small private sector in Malawi that is largely comprised of faith-based hospitals (Christian Health Association of Malawi, CHAM) that function almost as an extension of the public health sector, although with small fees for services and medicines [41,44]. The reason for this finding is unclear, but again could merit investigation in future facility-based research.

For child’s symptoms, both our analysis and the recent national facility study indicate reduced odds of ACT treatment when febrile children have reported respiratory symptoms. Our study further shows differences in the effect of diagnostic testing when introduced for children with fever alone versus fever with respiratory symptoms. Current results indicate significantly higher ACT treatment odds among tested pediatric fevers compared to untested when respiratory symptoms are also reported, while there was no significant difference in ACT use among tested and untested children with fever alone (Table 3). This result is difficult to explain, but does capture a phenomena documented in facility-based research and reinforces the influence of respiratory symptoms on testing and treatment practices in Malawi during this time period. This result could reflect the issue of illness severity whereas tested cases are more severely ill and more likely to receive treatment. Alternatively, it is also possible that to even perform a malaria diagnostic test on a child with fever and respiratory symptoms (often indicative of bacterial respiratory infections) may mean there is a higher malaria suspicion that also leads to malaria treatment. This finding may merit further investigation in qualitative studies or facility-based research.

**Mozambique**

Mozambique had relatively high malaria treatment odds for tested pediatric fevers compared to untested cases among all studied countries. There was no evidence of an interaction between diagnostic testing and investigated variables in this country dataset, which may in part be due to insufficient power to detect such differences. There are few published studies from Mozambique to help explain results [16] but findings were viewed as plausible given the program context and malaria epidemiology. *“There are three main reasons I can think of that could explains these results: (1) high malaria prevalence in certain areas even in the dry season (2) poor case management practices (3) access to testing and care (rural, bad infrastructure, hard to get tested and treated)”*

Malaria is highly endemic throughout most of Mozambique with transmission occurring throughout the year [42,44]. Diagnosis was adopted as part of the national strategy in 2006. However, widespread mRDT availability and use was not expected at the time of survey fieldwork with reported ongoing distribution delays and widespread stock outs [42,44]. In terms of malaria confirmation by microscopy at hospitals, there is documented low quality facility blood smears and common stock-outs of laboratory equipment.

Given weak supply systems, it is probable that febrile children reaching sources with diagnostic services may also have better access to medicines than untested cases. These children may also have more severe illness if diagnostic services are then concentrated at referral hospitals. Moreover, it is further probable that children reaching these sources of care are more severely ill given the general difficult access to facilities in Mozambique. Community-based treatment programs to improve access to diagnosis and care started only after survey fieldwork. Finally, diagnostic test adherence was perceived as poor despite limited evidence to date [16]. *‘(In Mozambique) there was little experience with testing at peripheral facilities at this time (with the lack of widely available mRDT stocks), and poor case management practices in general’*

**Rwanda**

In Rwanda, there was a non-significant reduction in malaria treatment for tested pediatric fevers compared to untested cases, along with significantly higher odds of antibiotic treatment associated with testing (Table 2). At the sub-national level, in the private sector, there were significantly higher anti-malarial use odds among tested pediatric fevers compared to untested cases, while there was a non-significant decline associated with testing at public sources (Table 3).*“The private sector is small and more developed in Kigali. Clients attending these facilities often expect or demand certain medicines and some health providers want to maintain client satisfaction.”*

Rwanda has experienced major declines in malaria transmission since around 2008 due to wide-scale deployment of malaria control interventions throughout the country [61]. Around this time, nationwide mRDT scale-up also began and Rwanda’s health system therefore had extensive experience with malaria diagnostic testing, including at peripheral facilities and by community health workers, at the time of our study (September 2010-March 2011). Community health workers generally use point-of-care diagnostic tests while health facilities use microscopy to diagnose malaria. While there is limited evidence on case management practices from Rwanda, adherence to test results was perceived as relatively good compared to other countries. USAID also reported frequent trainings and routine supervisory visits to help support quality case management practices [44]. Finally, widespread stock-outs of essential commodities (including mRDT or ACT) were seen as very unusual occurrences.

It is also well established that Rwanda has a dynamic, disciplined health system that is able to deliver essential services to much of its population, including poor and marginalized families [58]. This is credited to a combination of factors, including national health insurance, performance-based financing approaches, community health worker networks and mobile technologies. Widespread access to testing and care, coupled with low malaria transmission and plausibly good test adherence practices, could explain Rwanda’s results at the population level.

**Uganda**

Uganda had non-significant reductions in ACT use among tested pediatric fevers compared to untested cases, although higher use of any anti-malarial or antibiotic drugs associated with diagnostic testing (Table 2). At the sub-national level, there were differences in the effect of diagnostic testing on ACT and antibiotic treatment across malaria risk contexts. In moderate-risk settings, ACT treatment odds significantly declined by 33% for tested pediatric fevers compared to untested cases, while this effect was negligible in high-risk settings. Similarly in moderate-risk settings, antibiotic treatment odds was 1.68 times higher for tested pediatric fevers compared to untested cases, while this effect was negligible in high-risk areas (Table 3).

Uganda has variable malaria transmission ranging from highly endemic in the northern regions to low or unstable transmission in the south, west and eastern portions of the country. It is plausible that diagnostic testing could have a different association with ACT use across different risk contexts within Uganda, which is supported by other published studies as well [43-44].

At the time of survey fieldwork (June – December 2011), wide-scale mRDT deployment had not yet begun. Therefore, data in our analysis mostly reflects microscopy that was available at health centers (HC-III) and higher-level facilities. Poor adherence practices with both microscopy and mRDT have been documented in published research [12,39], although these findings are not based on national-level facility surveys like in Benin and Malawi. It should be noted that microscopy is considered the ‘gold standard’ diagnostic tool for malaria even post-mRDT scale-up in 2012 [43-44].

In terms of access to testing and care, public health facilities have been free-of-charge to clients since 2001, which as increased their overall utilization [43,62]. However, inconsistent ACT supplies have been commonly reported at peripheral facilities around the time of survey fieldwork [44]. This could affect results such that there could be slightly reduced ACT use odds among tested cases at facilities with microscopy services but without medicine stocks (compared to untested cases potentially presenting in the informal sector where ACTs have still been available but without diagnostics). Moreover the issue of stock-outs, coupled with other barriers to service utilization [62], leads many families to still seek initial care at drug shops where diagnostic tests are near absent. While our analysis accounted for this issue (see methods section), the use of multiple sources is likely underreported in survey interviews and could still affect results as described.
